# 25 Years of Cardiac Ion Channel QSAR: From hERG Dominance to Multi-Channel Modeling

**DOI:** 10.1021/acs.jcim.6c03072

**Published:** 2026-09-17

**Authors:** Mateusz Iwan, Francesca Grisoni, Anastasia Pentina, Marina Garcia de Lomana, Alessandra Roncaglioni

**Affiliations:** † Institute for Complex Molecular Systems (ICMS), Department of Biomedical Engineering, 3169 Eindhoven University of Technology, P.O. Box 513, Eindhoven 5600 MB, The Netherlands; ‡ 1569 Machine Learning Research, Research and Development, Bayer AG, Müllerstr. 178, Berlin 13353, Germany; § Department of Environmental Health Sciences, Istituto di Ricerche Farmacologiche Mario Negri IRCCS, Via Mario Negri 2, Milan 20156, Italy

**Keywords:** hERG, cardiac ion channels, multi-channel modeling, proarrhythmic risk, CiPA, QSAR, machine learning, deep learning

## Abstract

In 2013 the Comprehensive *In Vitro* Proarrhythmia Assay (CiPA) initiative proposed multi-channel assessment for proarrhythmic risk evaluation, yet ligand-based predictive modeling has remained largely single-channel. To quantify and explain this mismatch, we survey 155 ligand-based modeling studies on cardiac ion channels (including conventional QSAR, machine learning, and pharmacophore- and other 3D ligand-based approaches) published between 2001 and mid-2026. Across this period, 88.4% of studies target *hERG* as their sole ion channel endpoint, a proportion that reflects data availability, regulatory incentives, and research inertia rather than scientific necessity. Although public data for *Na*_*v*_ *1.5* and *Ca*_*v*_ *1.2* were sufficient for modeling years earlier, both channels received only isolated studies until 2022. Since then, multi-channel modeling has expanded substantially, with multiple groups now addressing the primary channels (*hERG*, *Na*_*v*_ *1.5*, and *Ca*_*v*_ *1.2*) jointly. In contrast, the secondary channels (*K*_*v*_ *7.1*, *K*_*v*_ *4.3*, and *Kir2.1*) remain largely understudied: *K*_*v*_ *4.3* and *Kir2.1* are entirely unmodeled in the surveyed literature, and models for *K*_*v*_ *7.1* remain scarce, likely due to the sparsity and heterogeneity of public data. A retrospective analysis of the reported model performance found no discernible improvements attributable to methodological advances. Reliability-related aspects, such as applicability domain and uncertainty quantification, were also rarely addressed. The field should treat joint modeling of the primary channels as the expected standard rather than a novel contribution. Other important aspects include the development of standardized benchmarks for multi-channel evaluation, integration with physiological models, and greater emphasis on prediction reliability. For the secondary channels, progress would additionally require coordinated data release or federated learning, methods suited to data-scarce targets, and community coordination, none of which individual research groups can establish alone.

## Graphical abstract

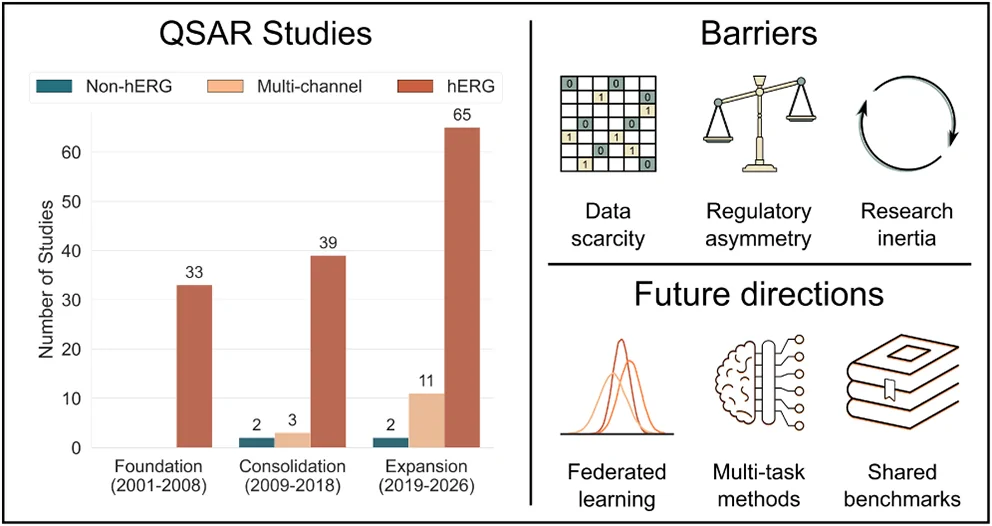

## 1 Introduction

Between 1997 and 2001, at least four marketed drugs (terfenadine, cisapride, astemizole, and grepafloxacin) were withdrawn from the market because of their association with Torsades de Pointes (TdP), a potentially fatal cardiac arrhythmia.^1,2^ Later work identified a common mechanism: block of the human ether-à-go-go-related gene (*hERG*) potassium channel, which conducts the rapid delayed rectifier current (*I*_*Kr*_), essential for cardiac repolarization.^3,4^

In response, the International Council for Harmonization (ICH) issued guidelines S7B and E14, mandating *in vitro* and *in vivo* non-clinical assessment of cardiac repolarization effects (including *hERG* channel block) and clinical monitoring of QT interval changes for new drug candidates.^5,6^ In the period since their implementation in 2005, and through at least 2021, no new drugs were withdrawn because of delayed repolarization.^7^

Assessing *hERG* block alone cannot explain why some potent *hERG* blockers, such as verapamil, do not cause TdP.^8^ Subsequent research revealed that the effects of *hERG* block could be offset by concurrent block of other cardiac ion channels, a compensation mechanism that single-channel assessment cannot capture.^9–11^

To investigate these multi-channel effects, the Comprehensive *In Vitro* Proarrhythmia Assay (CiPA) initiative was launched in 2013 as an international collaborative effort, following an FDA-organized workshop. The initiative involved regulatory agencies, pharmaceutical companies, and academic institutions across multiple countries.^12^ The CiPA framework proposed evaluating proarrhythmic risk through three integrated components: (1) *in vitro* patch-clamp assessment of effects on multiple cardiac ion channels, (2) *in silico* action potential modeling integrating these effects, and (3) confirmatory assays using human induced pluripotent stem cell-derived cardiomyocytes.^13^

The proposed framework has been progressively adopted in pharmaceutical non-clinical assessment; industry surveys by Wallis et al. indicate increasing evaluation of drug candidates against broader cardiac ion channel panels beyond *hERG*.^14^ In parallel, regulatory pathways now allow companies to waive certain clinical QT interval studies when comprehensive non-clinical data (including multi-channel *in vitro* and *in vivo* assessments) demonstrate low torsadogenic risk.^15^ The adoption of *in silico* modeling, however, has lagged behind experimental approaches.^14^ The same pattern appears in the literature: until approximately 2022, computational models remained overwhelmingly focused on *hERG*.

This review surveys quantitative structure-activity relationship (QSAR) and machine learning (ML) models developed to predict drug activity at cardiac ion channels relevant to proarrhythmic risk. We focus on the six channels central to CiPA: *hERG* (*K*_*v*_ *11.1*), *Na*_*v*_ *1.5*, *Ca*_*v*_ *1.2*, *K*_*v*_ *7.1*, *K*_*v*_ *4.3*, and *Kir2.1*. Throughout this review, we refer to *hERG*, *Na*_*v*_ *1.5*, and *Ca*_*v*_ *1.2* as *primary channels*, as they are the most frequently blocked at clinically relevant concentrations and account for the majority of drug-induced TdP risk.^11^ We refer to *K*_*v*_ *7.1*, *K*_*v*_ *4.3*, and *Kir2.1* as *secondary channels*, as they are rarely blocked under therapeutic conditions but may become relevant under specific circumstances.^16^ Our scope included ligand-based predictive modeling studies, including conventional QSAR and ML methods, as well as relevant pharmacophore and other 3D ligand-based approaches. Detailed criteria are described in Section 3.

Against this background, we trace the historical development of cardiac ion channel QSAR, examine the methodological and infrastructural factors that shaped the field, assess the limits of cross-study performance comparisons, and identify the data, benchmarking, and modeling priorities needed to support multi-channel frameworks such as CiPA. We find that the near-exclusive focus on *hERG* persisted far longer than data availability justified, that reported model performances show no discernible improvement attributable to methodological advances, and that aspects critical for regulatory use (applicability domain assessment and uncertainty quantification) remain largely unaddressed.

## 2 Ion Channels and Proarrhythmic Risk

### 2.1 Electrophysiological Basis

The cardiac action potential (AP) is a transient change in the membrane voltage that coordinates the electrical activity of the heart. In ventricular myocytes, the AP comprises five phases (Figure 1), each governed by distinct ion currents that flow through specific ion channels.

**Figure 1:**
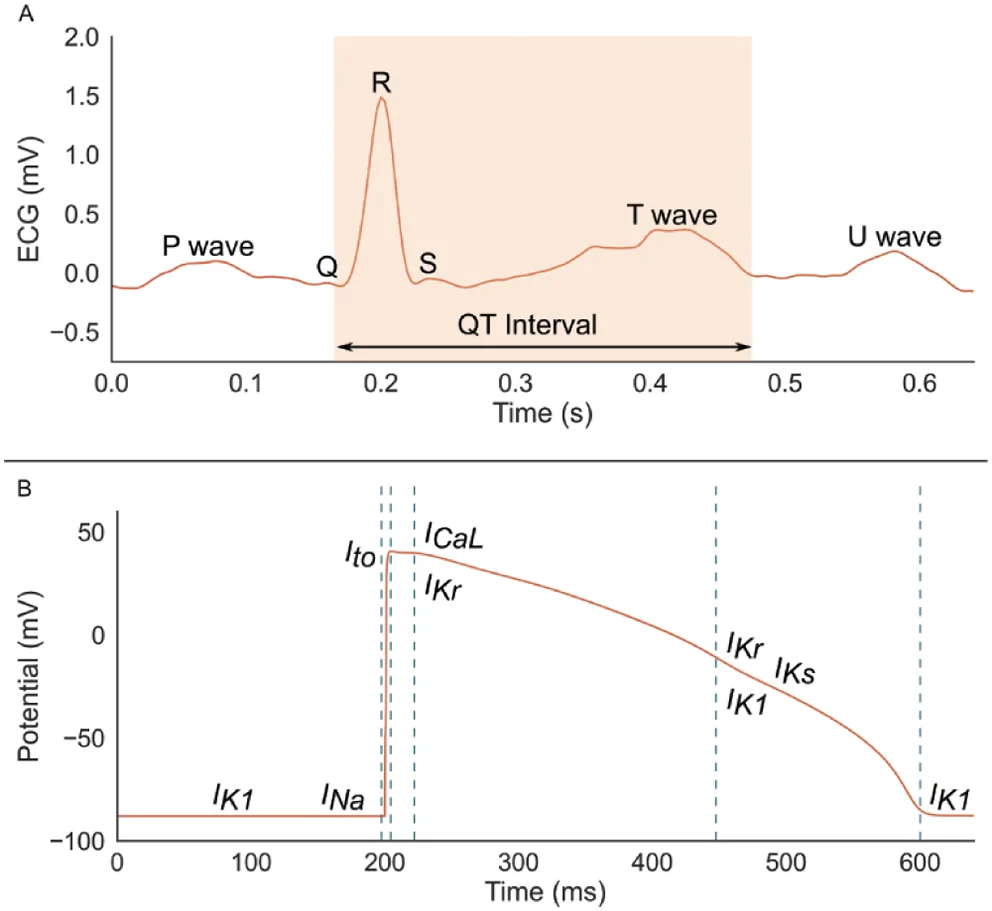
Electrocardiogram (ECG) and ventricular action potential (AP). Panel A: single cardiac cycle showing P wave (atrial depolarization), QRS complex (ventricular depolarization), and T wave (ventricular repolarization).^17^ Panel B: simulated human ventricular AP using the CiPA-modified O’Hara–Rudy model (aligned with the ECG time scale), with phases 0–4 and contributing ion currents indicated.^18^

The shape and duration of the AP depend on the balance between depolarizing (inward) and repolarizing (outward) currents (Table 1). Because this balance involves multiple channels, *hERG* block alone cannot be reliably used to predict the proarrhythmic risk. If a compound blocks only the *hERG* channel, the repolarizing current is reduced, which prolongs the AP and increases the QT interval. If the same compound additionally blocks inward currents such as *I*_*CaL*_ or *I*_*Na*_, the reduced depolarizing drive may partially or fully offset the effect of the *hERG* block.^9^

**Table 1:** Major Cardiac Ion Channels Relevant to Drug Safety Assessment^a^

Phase	Current	Channel	Gene	Role	Effect of Block
0	*I*_*Na*_	*Na*_*v*_1.5	*SCN5A*	Depolarization	Slowed conduction
1	*I*_*to*_	*K*_*v*_4.3	*KCND3*	Early repolarization	Altered notch
2	*I*_*CaL*_	*Ca*_*v*_1.2	*CACNA1C*	Plateau	Shortened APD
2–3	*I*_*Kr*_	*hERG*	*KCNH2*	Repolarization	Prolonged APD, TdP risk
2–3	*I*_*Ks*_	*K*_*v*_7.1	*KCNQ1*	Repolarization	Prolonged APD
3–4	*I*_*K*1_	*Kir2.1*	*KCNJ2*	Resting potential	Altered resting potential

a APD—Action potential duration. TdP—Torsades de pointes. For detailed channel physiology, see, for example, Lei et al.^19^

Even a perfectly accurate *hERG* model will overestimate TdP risk when compensatory multi-channel effects are present, because single-channel prediction cannot capture integrated system-level effects. The scale of this problem is substantial: one estimate from the early post-ICH S7B/E14 era suggests that up to 60% of new molecular entities that tested positive for *hERG* block were abandoned in early development,^20^ some of which may have been safe *in vivo* due to compensatory multi-channel effects. Although the potential for *hERG*-based screening to discard otherwise viable drug candidates is well recognized,^13,14,21^ its exact extent has not, to our knowledge, been formally investigated.

### 2.2 Structural Basis of Cardiac Ion Channel Pharmacology

Cardiac ion channels share common structural features but exhibit subtle differences that determine their ion selectivity, voltage sensitivity, and drug binding properties.^22^ They typically consist of pore-forming α subunits, some of which are accompanied by regulatory β and/or auxiliary subunits that modulate channel function. Structurally, the following key regions can be distinguished:

- A pore-forming domain consisting of transmembrane segments and connecting loops that determine ion selectivity and conductance.
- A selectivity filter located in the narrowest part of the channel, consisting of amino acids that ensure only selected ions can pass through.
- Voltage-sensing domains (in voltage-gated channels) that detect membrane potential changes due to the presence of charged residues.
- Phosphorylation and binding sites for auxiliary subunits and regulatory proteins.

Among these channels, *hERG* is structurally distinctive in ways that help explain its frequent off-target inhibition. Unlike most other voltage-gated potassium channels, it has an unusually small central cavity flanked by four deep hydrophobic pockets.^23^ It also lacks a short amino acid sequence (Pro-Val-Pro) that normally narrows the channel entrance in related channels; this absence leaves the pore more accessible to drug molecules.^24^ Within the cavity, aromatic residues on the inner helix, particularly Tyr652 and Phe656, provide favorable contact points for a wide range of drug molecules,^25^ while a region of negative charge beneath the selectivity filter helps attract and hold positively charged blockers.^23,26^ In addition, the gating of *hERG* is unusual: it inactivates rapidly but deactivates slowly, a behavior partly regulated by interactions between two intracellular domains (the N-terminal PAS domain and the C-terminal CNBHD).^27^ The combination of these features (accessible pore, versatile binding contacts, and prolonged open/inactivated states) explains why many otherwise unrelated compounds bind to and block *hERG*.

## 3 Survey Scope and Methods

We surveyed the literature through searches on Scopus, Web of Science, and Google Scholar, using combinations of (1) *QSAR*, *machine learning*, *deep learning*, *prediction*, *regression*, and *classification* (methodology-related) and (2) *hERG*, *cardiac ion channel*, and individual channel names (target-related). Terms within each category were combined using OR, and the two categories were then combined using an AND. The first author manually screened retrieved records based on titles and abstracts and assessed potentially relevant studies in full text for inclusion. Initial searches were supplemented with backward and forward citation tracking. Searches were last updated at the end of June 2026 and covered publications from 2001 to mid-2026.

We included studies that developed ligand-based predictive models for compound activity at CiPA-relevant cardiac ion channels, encompassing conventional QSAR, ML, pharmacophore-based, and other 3D ligand-based methods. Structure-based approaches (e.g., docking, molecular dynamics, free-energy calculations) were included only when they contained a relevant ligand-based modeling component and were frequently cited within the surveyed literature. Studies relying exclusively on structure-based methods were excluded, as were studies focused on general cardiotoxicity endpoints and noncardiac isoforms.

Our survey identified 155 studies that developed predictive models for cardiac ion channel activity. The distribution of modeled channels is highly imbalanced: 137 studies (88.4%) target *hERG* exclusively, while only 14 (9.0%) address two or more CiPA channels, most of which focus on the primary channels jointly. The remaining four studies model single non-*hERG* channels: two address *Na*_*v*_ *1.5*, and one each addresses *K*_*v*_ *7.1* and *Ca*_*v*_ *1.2*. We found no studies for *K*_*v*_ *4.3* or *Kir2.1*.

## 4 Historical Trajectory

We organize this historical survey into three periods defined by shifts in methodology and data availability: Foundation (2001–2009), Consolidation (2010–2018), and Expansion (2019–mid-2026). Full study-level details are given in Table S1 (methods and targets) and Table S2 (datasets). Table 2 summarizes the methodological evolution across the three periods.

**Table 2:** Evolution of Cardiac Ion Channel QSAR by Period^a^

Period	Models	Descriptors	Data (n)	Channels
2001–2009	PLS, DT, SVM	Physicochemical, Pharmacophores	<500	*hERG*
2010–2018	SVM, RF, GBM	Circular, Field-based, Path-based	500–6700	*hERG*, *Na*_*v*_ *1.5*, *Ca*_*v*_ *1.2*, *K*_*v*_ *7.1*
2019–mid-2026	DNN, GCN, XGB	Graphs, Learned embeddings	5000–34000	*hERG*, *Na*_*v*_ *1.5*, *Ca*_*v*_ *1.2*, *K*_*v*_ *7.1*

a Columns indicate methods and representations that emerged or achieved widespread adoption; established approaches from prior periods typically remained in use. Data (n) indicates the approximate size of hERG datasets.

A recurring observation across all three periods is that methodological transitions in cardiac ion channel modeling have largely mirrored trends in the cheminformatics and machine learning communities. The advances (from kernel machines through ensemble learners and boosting algorithms to deep learning approaches) were applied in the field without substantial modifications. Representations motivated by known *hERG* binding characteristics were present throughout but never dominant, and their decline relative to scalable 2D approaches was driven by practical rather than scientific considerations.

The boundaries between periods are not sharp: approaches introduced in one often persisted into the next, and newer methods typically supplemented rather than replaced older ones. The periodization instead captures how the dominant data sources, molecular representations, and modeling frameworks shifted over time.

### 4.1 Foundation (2001–2009)

The first QSAR models for the *hERG* channel appeared in 2002. Two independent groups developed three-dimensional pharmacophore models to predict *hERG* block.^28,29^ In the same year, Roche et al. applied methods such as partial least squares (PLS) regression and multilayer perceptron (MLP) to a proprietary dataset.^30^

Subsequent studies adopted diverse algorithms, particularly decision trees (DTs)^31–34^ and support vector machines (SVMs).^35–37^ Bayesian modeling approaches were also used relatively frequently,^38–40^ while PLS remained relevant throughout the period.^41–43^

Molecular representations were also diverse. Physicochemical descriptors calculated using software packages such as MOE, DRAGON, and VolSurf were the most widely adopted.^30,34,44–46^ Other popular approaches involved grid-based and pharmacophoric features^42,47–50^ or quantum-chemical properties^36,51–54^ derived from 3D structures. In addition, some studies relied on circular or fragment-based features.^33,46,55^

This methodological diversity occurred despite limited public data. Most studies relied on fewer than 200 compounds, usually collated from primary experimental literature or reused from previous publications.^1,28,44^ The largest public dataset of the period contained 414 compounds;^31^ the largest proprietary set included 1979 compounds.^56^ Data scarcity encouraged methodological experimentation but limited opportunities for a controlled comparison between approaches.

The coexistence of pharmacophore-based, quantum-chemical, and conventional physicochemical representations was not incidental. *hERG* blockers are known to share broad structural features, with lipophilicity and a basic nitrogen in an appropriate spatial arrangement being among the most consistently cited binding indicators.^25^ The prevailing assumption in the early computer-aided drug design (CADD) field was that representations encoding three-dimensional and electronic properties would be more informative for a target with known structural selectivity requirements. This was a hypothesis carried over from the broader field rather than a conclusion drawn from ligand-based evidence for *hERG* specifically, and data scarcity left it unresolved.

### 4.2 Consolidation (2010–2018)

From 2010 to 2018, the field changed primarily through data growth rather than through methodological innovations. The expansion of public chemical databases (such as ChEMBL^57^ and PubChem^58^) transformed dataset assembly from laborious literature curation to systematic database queries. Public datasets for *hERG* grew from hundreds to thousands of compounds: 2644 in 2010,^59^ 4415 in 2013,^60^ and 6690 by 2016.^61^

Algorithmic approaches consolidated around methods established in the previous period. Support vector machines remained dominant,^62–65^ with random forests^60,66,67^ and similarity-based methods such as k-nearest neighbors^68–70^ gaining popularity. Gradient boosting machines^61,71,72^ and the first deep neural networks^71^ appeared late in the period but did not yet overtake classical approaches in popularity.

Molecular representations followed a similar pattern. Physicochemical descriptors and topological indices established in the previous period remained dominant.^60,63,73,74^ Extended-connectivity fingerprints (ECFPs) gained widespread adoption,^75–77^ while three-dimensional approaches, including pharmacophore-based features, declined in relative frequency.

Despite larger datasets and more mature methods, the field remained narrowly focused on *hERG*. Single-channel studies of *K*_*v*_ *7.1* ^78^ and *Ca*_*v*_ *1.2* ^79^ were rare exceptions. Multi-channel efforts were equally sparse: Obiol-Pardo et al. modeled *hERG* and *K*_*v*_ *7.1* jointly,^80^ and two groups developed models spanning *hERG*, *Na*_*v*_ *1.5*, and *Ca*_*v*_ *1.2*.^71,81^ The regulatory shift toward multi-channel assessment was not yet reflected in the broader modeling literature.

Model dissemination practices have changed during this period. Earlier studies typically published model specifications within papers as regression coefficients, decision-tree rules, or pharmacophore definitions. From 2013 onward, studies released executable code and public platforms: to our knowledge, Czodrowski et al. published the first open-source *hERG* modeling code,^60^ and Braga et al. launched the first public prediction platform in 2014.^64^

The shift toward scalable 2D representations was not preceded by evidence that they outperformed 3D approaches on comparable data; it reflected the practical incompatibility of conformer-dependent methods with large, heterogeneous ChEMBL-derived datasets. Whether the predictive signal in such datasets is predominantly carried by gross physicochemical features or by finer pharmacophoric geometry remains unexamined, so does the question first raised in the Foundation period: whether 3D and electronic representations are more informative than 2D descriptors for *hERG*. That the CiPA initiative (2013) produced no broader computational response in this period points to a separate conclusion: the absence of a mandatory requirement, rather than any methodological obstacle, kept the field mostly focused on a single ion channel.

### 4.3 Expansion (2019–Mid-2026)

Two developments stand out in the 2019–mid-2026 period: the adoption of deep learning architectures and the expansion of modeling beyond *hERG*, particularly toward three-channel CiPA-relevant frameworks.

Regarding the first of these, graph neural networks (GNNs) have been the predominant deep learning approach, which reflects the natural correspondence between molecular graphs and GNN architectures. GNN variants, including graph convolutional networks,^82–84^ graph attention networks,^85,86^ and message-passing neural networks,^87,88^ have been widely explored. Various groups also adapted architectures from computer vision (CNNs operating on molecular images^89,90^ ) and natural language processing (RNNs processing SMILES strings).^88,91^ More recently, several groups have combined heterogeneous representations (graphs, fingerprints, and SMILES) in multimodal architectures.^92–97^

Alongside conventional fingerprints and descriptors, recent studies have incorporated learned molecular representations as model inputs. These include latent embeddings from variational autoencoders (VAEs)^94,96,98^ and representations derived from large language models (LLMs) trained on SMILES or protein sequences.^99,100^

Modeling beyond *hERG* has expanded during this period, from isolated single-channel studies to integrated frameworks more aligned with the CiPA multi-channel paradigm. The first models targeting *Na*_*v*_ *1.5* as a primary endpoint appeared in 2020 and 2022, both framed as classification tools motivated by the cost and throughput limitations of patch-clamp screening.^101,102^ The broader shift toward three-channel modeling began with studies by Arab et al. Across three successive publications, they first modeled *hERG* and *Na*_*v*_ *1.5*, then expanded to include *Ca*_*v*_ *1.2* in the subsequent two. They also benchmarked molecular representations across all three channels and explored semi-supervised learning to exploit large unlabeled chemical datasets.^93,103,104^ Each study released curated datasets and open-source code; this likely lowered the barrier for subsequent groups to adopt the same three-channel scope.

Several groups have since developed integrated models for the primary channels, predominantly employing multimodal architectures that combine SMILES, molecular graphs, and fingerprints.^94,95,105,106^ One study took a distinct approach: it used *Na*_*v*_ *1.5* and *Ca*_*v*_ *1.2* models as components of a generative framework for redesigning compounds with reduced *hERG* liability.^96^ The primary-channel scope has become more commonplace, reflecting data availability and the compensation mechanism central to CiPA’s rationale. One of the most recent developments was the inclusion of *K*_*v*_ *7.1* in a multi-task scenario, treated as exploratory due to small dataset size.^107^

Datasets have continued to grow, though proportional gains are smaller than in 2010–2018. ChEMBL-derived compilations have become a new standard.^108–110^ Agarwal et al., for instance, assembled a three-channel dataset comprising over 34000 *hERG*, approximately 3200 *Na*_*v*_ *1.5*, and roughly 1600 *Ca*_*v*_ *1.2* entries.^111^ Previously curated datasets were frequently repurposed as external test sets, and open-source code, trained models, or prediction platforms now accompany the majority of recent studies.

The deep learning adoption sequence here mirrors molecular property prediction broadly, with no cardiac-specific rationale driving the shift to GNNs or multimodal fusion. Pharmacophoric features reappear in a handful of recent studies,^90,94,95^ but as one component among several in multimodal inputs rather than a deliberate primary choice—a role qualitatively different from their prominence in the Foundation period. Beyond these methodological changes, the expansion from single-channel to three-channel modeling appears to have been facilitated by the release of curated datasets and open-source code, though their relative contributions cannot be disentangled from the survey data.

## 5 Retrospective Analysis

The survey presented in Section 4 documents what methods were used and when. In this section, we investigate whether the methodological transitions produced measurable improvements in predictive performance and how reliably cross-study comparisons can be drawn given the evaluation practices of the field. The practical motivation for this assessment is that selecting the best-performing models for each channel is a necessary step toward building reliable multi-channel pipelines for TdP risk prediction.

### 5.1 Statistical Analysis of Performance

A persistent limitation is the absence of controlled comparisons among methodological approaches. Studies differ in modeling approaches (classification vs regression), dataset composition, activity thresholds used (for classification), and evaluation strategies. To examine performance patterns across this heterogeneous literature, we collected information on model performance alongside selected design choices (Supporting Information, Tables S3 and S4). From each study, we included the reported performance on at least one test set. We used the model designated by the authors as the best; where no such designation was made, the first author selected the best model based on the reported metrics. Figure 2 shows the reported performance over time, with LOWESS trend lines included as a descriptive aid where at least five data points were available.

**Figure 2:**
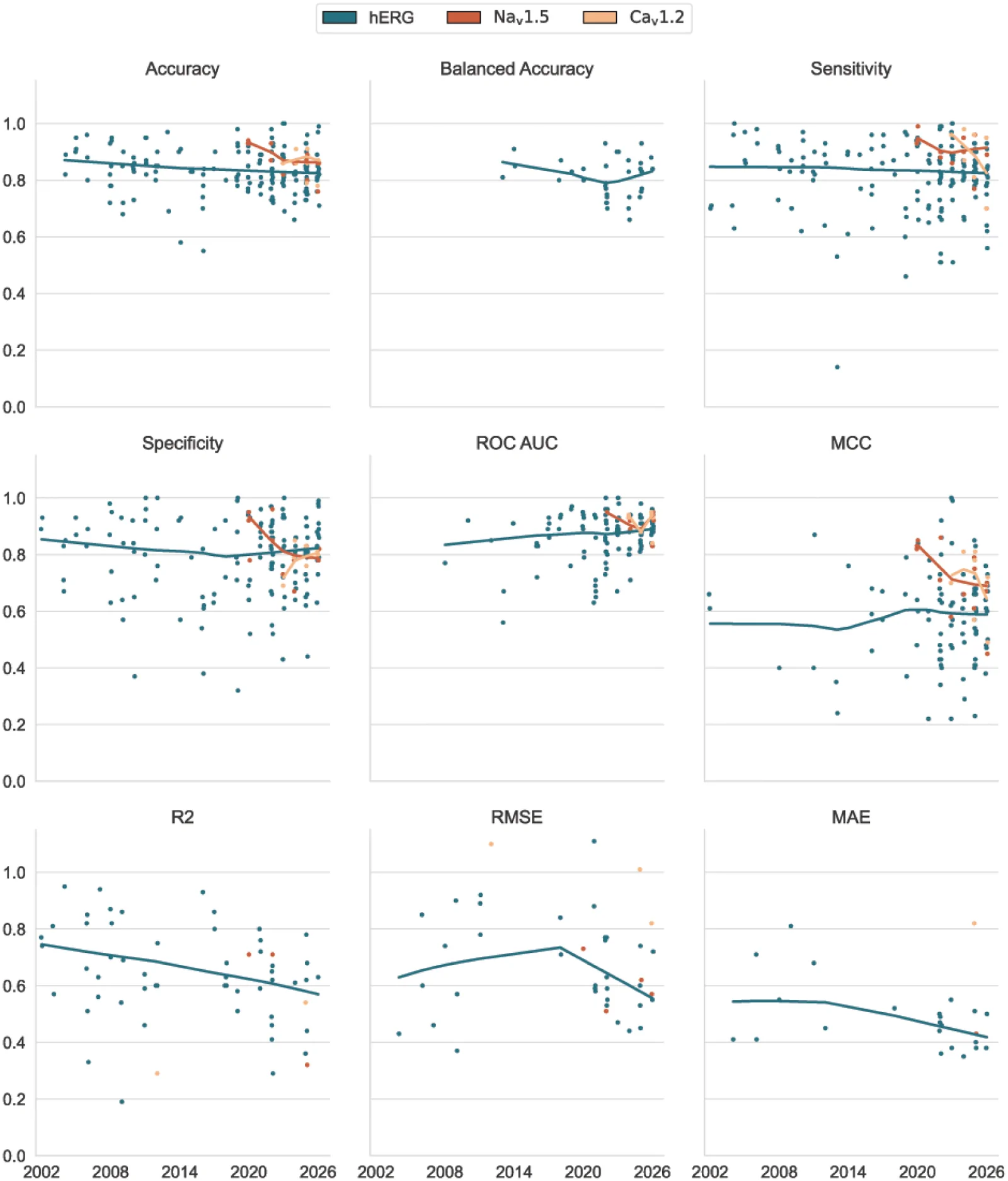
Reported test-set performance of the best model per study over time, across the primary channels. Each point represents a single evaluation; multiple evaluations per study are included where authors reported results on more than one test set. Trend lines are LOWESS smooths fitted where at least five data points were available.

To investigate whether statistically discernible differences were associated with specific study design choices or modeling era, we performed pairwise comparisons of reported performance across related comparison groups, such as machine learning vs deep learning or random vs distance-based splitting. For each such pair, we used a two-sided Mann–Whitney *U* test^112^ to check for differences between distributions of reported metrics and rank-biserial correlation^113^ to quantify effect sizes. Resulting *p*-values were adjusted using Benjamini–Hochberg correction,^114^ with each performance metric treated as a family of related tests. A discernibility threshold of 0.05 was used for the adjusted *p*-values. The full results of statistical comparisons are given in Supporting Information, Tables S5 and S6.

Of 99 pairwise comparisons considered, 24 were excluded because of insufficient group size (*N* < 3). No statistically discernible differences were found between eras or between model classes for any reported metric. After multiple-testing correction, only three comparisons remained statistically discernible, all involving evaluation strategy or activity threshold. For classification, reported accuracy was higher for studies using random splits than external test sets (*r*_*rb*_ = 0.34, *p*_*adj*_ = 0.038), and for studies employing a 1 μM threshold rather than 10 μM (*r*_*rb*_ = 0.71, *p*_*adj*_ = 0.026). For regression, reported RMSE was lower for studies using random splits than external test sets (*r*_*rb*_ = 0.88, *p*_*adj*_ = 0.013).

These findings are better interpreted as a limitation of this analysis than as substantive conclusions. The absence of discernible differences between the investigated design choices does not imply that methodological advances produced no improvement. For instance, models trained on tens of thousands of compounds would be expected to outperform older ones trained on a few dozen, simply by virtue of having seen a larger portion of chemical space. This improvement is obscured by heterogeneous evaluation protocols, and the available data are insufficient to objectively disentangle it from genuine algorithmic differences. This in turn raises a broader question: whether the field’s published record is structured in a way that allows cumulative progress to be measured at all.

### 5.2 Comparability and Reproducibility

This limitation reflects the absence of standardized evaluation infrastructure against which progress could be measured. The closest approximations to standardized benchmarks currently available are previously curated datasets. For *hERG*, these include those of Doddareddy,^59^ Didziapetris,^61^ Cai,^108^ Munawar,^115^ Negami,^116^ Siramshetty,^91^ Ryu,^83^ and Karim.^84^ For *Na*_*v*_ *1.5* and *Ca*_*v*_ *1.2*, they include those of Arab.^93^ These datasets, however, reproduce rather than resolve the lack of standardized evaluation. The test sets of Arab et al., for instance, were constructed by excluding compounds with a Tanimoto similarity above 0.7 with respect to the training data. However, a model trained on a larger or more recent dataset may have training set compounds exceeding this similarity threshold with respect to the test set, which inflates its test set metrics relative to models evaluated under stricter criteria. Identifying a reference dataset that genuinely isolates algorithmic performance from data coverage is itself an unsolved problem.^117^

The field therefore lacks an analogue to MoleculeNet^118^ or CASP,^119^ community resources that transformed molecular property prediction and protein structure prediction, respectively, from collections of isolated results into fields with measurable progress trajectories. The recent emergence of multi-channel modeling offers a rare opportunity to establish such standards before the *Na*_*v*_ *1.5* and *Ca*_*v*_ *1.2* literature accumulates the same fragmentation that makes *hERG* benchmarking so intractable.

## 6 Barriers and Explanations

The historical survey reveals a field that has advanced methodologically while remaining, until recently, largely concentrated on a single channel. Three broad factors help explain this pattern: the unequal distribution of available data across channels, the regulatory and physiological context that made some channels more practically relevant than others, and the resulting research inertia that reinforced continued focus on *hERG*.

### 6.1 Data Landscape

#### 6.1.1 Quantity

The distribution of public data across cardiac ion channels is highly imbalanced. To examine this systematically, we curated bioactivity data from ChEMBL^57^ (version 36) for each CiPA channel through a multi-step filtering pipeline. The pipeline comprised assay-level annotation and classification, measurement-level standardization and outlier removal, and compound-level processing, as described in Supporting Information, Note S1. Figure 3 shows the cumulative number of unique compounds deposited per year for each CiPA channel.

**Figure 3:**
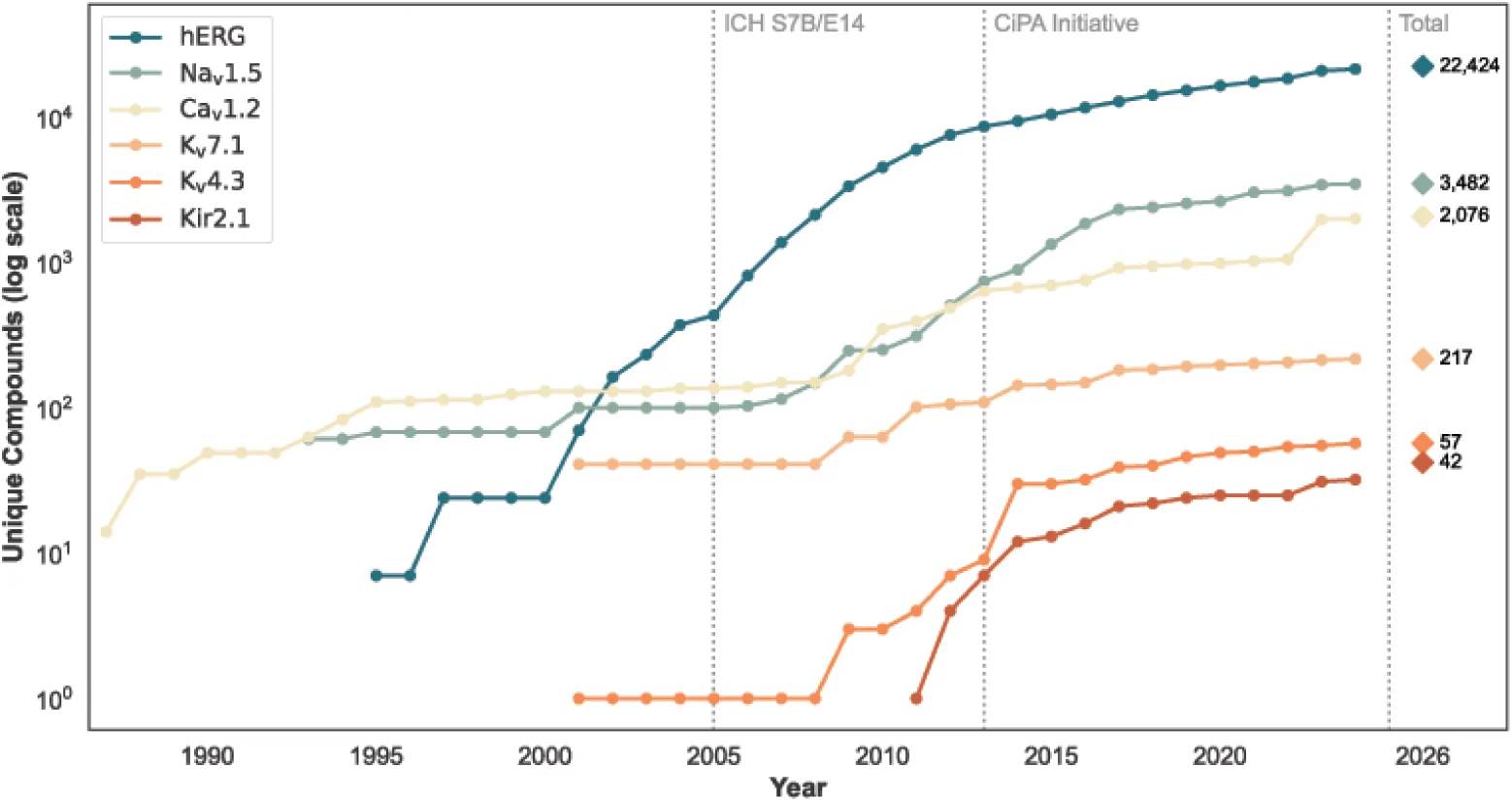
Cumulative unique compounds with at least one valid *IC*_50_, *K_i_, K_d_*, or single-concentration inhibition measurement per CiPA channel, after assay-level and measurement-level quality filtering (Supporting Information, Note S1).^57^ Documents with unknown deposition year were excluded from the time series; full counts including these entries are indicated by diamonds. Counts reflect unique ChEMBL molecule IDs and should be interpreted as upper bounds; additional study-specific filtering (e.g., activity threshold selection, removal of conflicting measurements, structure standardization, and deduplication) will reduce the number of compounds available for model training.

*hERG* dominates with over 22000 compounds, a result of two decades of mandatory screening. *Na*_*v*_ *1.5* and *Ca*_*v*_ *1.2* accumulated substantially fewer compounds (approximately 3500 and 2100, respectively). *Na*_*v*_ *1.5* deposition accelerated after 2013, while *Ca*_*v*_ *1.2* growth remained modest. The secondary channels (*K*_*v*_ *7.1* with 217, *K*_*v*_ *4.3* with 57, and *Kir2.1* with 42) remained at low levels throughout, reflecting both the recency of their systematic assessment and the rarity of deliberate drug development programs targeting these channels.

To better assess the extent of data scarcity for the secondary channels, we expanded the search beyond ChEMBL to include PubChem BioAssay and BindingDB (Supporting Information, Notes S2–S3). This broader search showed that additional data do exist for the secondary channels, but they remain limited and heterogeneous. For human targets, the number of unique compounds with potency-type measurements increased to 197 for *K*_*v*_ *7.1*, 60 for *K*_*v*_ *4.3*, and 22 for *Kir2.1*; including non-human measurements raised these totals to 263, 64, and 101, respectively. A detailed breakdown by source database, organism, and assay type is given in Table S7. While these channels cannot be described as entirely lacking public data, the available evidence is still far less modeling-ready than for the primary channels.

#### 6.1.2 Quality

Raw compound counts overstate the amount of usable data, as assays differ in quality and comparability. Electrophysiology measurements provide the most direct evidence of channel block, with manual whole-cell patch clamp widely accepted as the gold standard.^120^ Multiple electrophysiology formats exist, including manual and automated patch clamp, two-electrode voltage clamp, and related electrophysiological readouts, such as microelectrode arrays. Many datasets also include indirect proxy assays such as ligand displacement, ion flux, intracellular ion concentration changes, or membrane potential assays.^120^ *IC*_50_ or *K*_i_ values derived across these methods are not directly comparable without standardization, and even within patch-clamp measurements, differences in temperature or voltage protocol can substantially affect reported *IC*_50_ values.^121^

Encoding assay context during model training could partially address this issue, but a substantial fraction of ChEMBL’s assays lack sufficient metadata to unambiguously identify the underlying method. Manual curation of individual records is not practical given the sheer number of source papers.^122^ For the secondary channels, the situation is considerably worse. Our survey of medicinal chemistry articles revealed that while inhibition against the full cardiac ion channel panel is frequently assessed, its reporting is often limited to a single sentence noting no meaningful inhibition at the highest tested concentration.

#### 6.1.3 Implications for Modeling

*K*_*v*_ *7.1*, *K*_*v*_ *4.3*, and *Kir2.1* are difficult to model reliably using conventional approaches with current public data. *Na*_*v*_ *1.5* and *Ca*_*v*_ *1.2* have less data than *hERG* but enough for model development, as the counts in Figure 3 show. Still, *Na*_*v*_ *1.5* received minimal attention until 2020, and *Ca*_*v*_ *1.2* models have mostly emerged within three-channel efforts since 2022. This suggests that data availability alone does not determine modeling activity.

### 6.2 Explaining the hERG Focus

Beyond data availability, three additional factors contribute to this pattern. We infer these from the survey data and regulatory history rather than from controlled experiments, but the evidence is consistent.

#### 6.2.1 Regulatory Incentives and Requirements

The ICH S7B/E14 guidelines (2005) mandated *in vitro* repolarization assays (typically including *hERG* assays) and clinical QT studies for most new drug candidates. This resulted in an immediate demand for *hERG* models as early-stage screening tools. The CiPA initiative (2013) recommended multi-channel assessment as a more mechanistic approach but did not result in a mandatory regulatory requirement. The 2022 ICH E14/S7B Q&A document allows companies to waive clinical QT studies if comprehensive non-clinical data, including multi-channel assessment, demonstrate low proarrhythmic risk.^123^ This remains an optional pathway, though; companies can still meet regulatory requirements through *hERG* assessment and clinical QT studies alone. As a result, the regulatory minimum remains single-channel-centered.

The result is an asymmetric incentive structure. *hERG* models address a mandatory requirement and offer a clear regulatory utility. Models for other channels support an optional, albeit scientifically superior, assessment strategy. Until multi-channel assessment becomes a routine regulatory expectation rather than an optional pathway, computational efforts will naturally concentrate where regulatory demand is certain.

#### 6.2.2 Physiological Relevance and Blocking Frequency

Cardiac ion channels differ in both their susceptibility to drug blockade and the resulting proarrhythmic risk. A systematic assessment of 30 drugs from the CiPA reference compound set demonstrated that *hERG* is the channel most frequently blocked at clinically relevant concentrations (due to its structural characteristics as described in Section 2.2), followed by *Na*_*v*_ *1.5* and *Ca*_*v*_ *1.2*.^11^ The same drugs showed minimal or no block at therapeutic concentrations for *K*_*v*_ *7.1*, *K*_*v*_ *4.3*, and *Kir2.1*.

This blocking hierarchy helps explain why modeling has concentrated on *hERG*, *Na*_*v*_ *1.5*, and *Ca*_*v*_ *1.2*. Channels rarely blocked in clinical use generate less screening data and offer less practical utility for early-stage drug discovery. While the primary channels capture most of the drug-induced TdP risk, the secondary channels can become relevant under specific conditions: at supratherapeutic concentrations, under multi-channel block where nonlinear interactions amplify individual effects,^16^ or under physiological states such as β-adrenergic stimulation.^124^ They are also required by current action potential models (e.g., O’Hara–Rudy,^125^ T-World,^126^ which increasingly incorporate coupling between channel activity and downstream processes such as calcium handling and β-adrenergic signaling. Their inclusion in predictive models is therefore not as important as that of the primary three, but still valuable where data permit.

#### 6.2.3 Research Inertia

Two decades of *hERG*-focused research have produced accessible community infrastructure (widely adopted datasets, published baseline models, and broadly agreed performance expectations) that lowers the barrier to conducting additional *hERG* studies. For *Na*_*v*_ *1.5* and *Ca*_*v*_ *1.2*, such infrastructure is nascent: only a handful of curated datasets exist (primarily ChEMBL-derived compilations), which are reused across the limited number of studies. Even when scientific value might favor exploring undermodeled channels, the practical effort required to establish robust modeling pipelines favors incremental contributions to the well-established *hERG* literature.

## 7 Directions for Multi-Channel Modeling

Bringing multi-channel modeling to the same level of maturity as *hERG* prediction requires progress across several connected areas. The most immediate need is to consolidate the primary channel framework. Beyond this core, extending modeling to the secondary channels depends on data generation or federated learning and on methods suited to data-scarce targets. Integration with physiological models and attention to prediction reliability are required across both tiers.

### 7.1 Consolidating the Primary-Channel Framework

The primary channels capture the majority of drug-induced TdP risk and correspond to the CiPA-recommended panel. Sufficient public data are already available to model them reliably, as demonstrated by several groups.^96,104,106,107,111^ Modeling these channels jointly should become the default scope of new studies rather than a notable expansion.

The three-channel context offers an opportunity to establish the infrastructure the field has so far lacked. As the retrospective analysis showed (Section 5), the absence of standardized benchmarks, consistent activity thresholds, and common evaluation protocols has made it impossible to demonstrate cumulative progress even for *hERG*. The emerging three-channel literature, starting from a comparatively clean slate, is well positioned to build these standards from the outset, a possibility the field missed during the fragmented non-*hERG* efforts of 2010–2018.

Joint modeling also opens several methodological avenues that single-channel modeling does not. Because *hERG*, *Na*_*v*_ *1.5*, and *Ca*_*v*_ *1.2* share structural features in their inner cavities that give rise to correlated blocking profiles, information from one channel can potentially inform predictions for another. Approaches that could exploit this include shared molecular representations, multitask and hierarchical model architectures, auxiliary tasks encoding channel-binding-relevant properties (such as electrostatic profiles or membrane permeability), and transfer or pretraining strategies across the voltage-gated channel superfamily. Which of these approaches proves to be most effective, and in what combination, remains an open question.

### 7.2 Data Generation and Sharing for Secondary Channels

If modeling of *K*_*v*_ *7.1*, *K*_*v*_ *4.3*, and *Kir2.1* is to be pursued, it would require either new experimental campaigns or the release of existing proprietary data. The latter may be more immediately tractable: CiPA recommends testing new drug candidates against the full ion channel panel, so pharmaceutical companies now increasingly generate data for these channels. The release of even a fraction of these data, through precompetitive consortia or delayed public deposition, could make modeling feasible without requiring new experiments.

Direct data sharing faces substantial barriers. Proprietary screening data may reveal competitive intelligence about compound libraries, therapeutic programs, or assay protocols. A potential solution lies in federated learning approaches, as recently demonstrated in a collaboration involving multiple pharmaceutical companies.^127^ Federated distillation methods enabled collaborative model development without centralizing raw data and yielded models with improved generalizability across chemical series. Adapting such frameworks to *K*_*v*_ *4.3* and *Kir2.1* prediction could enable model training on proprietary data without requiring their release.

### 7.3 Methods for Data-Scarce Targets

Even with expanded data generation or federated approaches, the secondary channels will likely remain data-poor relative to *hERG*. The inter-channel approaches discussed above (transfer and pretraining strategies, multitask architectures, and auxiliary tasks) are even more relevant here, as predictions for these channels would depend largely on information borrowed from better-studied targets. The specific challenge is that insufficient data may exist for the secondary channels to serve as primary training data; therefore, methods designed for small-data regimes become especially important. Few-shot and meta-learning approaches, which adapt to new tasks from small support sets, have shown promise in molecular property prediction but remain untested for cardiac ion channels, despite the clear motivation that data scarcity for *K*_*v*_ *4.3* and *Kir2.1* provides.^128,129^ Structure-based approaches may also be more suitable for these targets due to lower data requirements, with their outputs used either in place of ligand-based descriptors or alongside them.

### 7.4 Integration with Physiological Models

CiPA’s vision extends beyond channel-level predictions to integrated proarrhythmic risk assessment using action potential models. In principle, QSAR-predicted *IC*_50_ values can be combined with free unbound plasma concentration estimates and Hill coefficients to compute fractional channel block via the Hill equation. These values feed directly into action potential models (e.g., O’Hara–Rudy^125^) to simulate drug effects on cardiac electrophysiology. This integration remains rare in practice.

One barrier is that human *C*_*max*_ is unavailable until the clinical stages. Provisional estimates from animal PK or computational ADME models could bridge this gap at earlier stages, although with additional uncertainty. Developing frameworks that propagate uncertainty from QSAR predictions through physiological simulations, or that provide risk estimates across plausible *C*_*max*_ ranges, could enable earlier integration of multi-channel data into safety assessment.

Integration with physiological models also highlights the complementary roles of the classification and regression models. While classification models (blocker/nonblocker) provide useful early-stage filters, physiological modeling requires quantitative potency estimates (e.g., *IC*_50_) to simulate dose-dependent effects. The field should maintain focus on regression approaches or develop models that output calibrated probability estimates across activity ranges. One approach would be ordinal classification across *IC*_50_ ranges with calibrated class probabilities, which would enable the downstream conversion to approximate quantitative inputs for physiological models.

### 7.5 Prediction Reliability and Regulatory Use

For models intended to support safety-relevant decision-making, predictive performance alone is not sufficient. One must also know whether a query compound falls within the model’s applicability domain and how uncertain the corresponding prediction is. Across the 155 studies surveyed, 29 (18.7%) reported some form of applicability domain assessment, while only 6 (3.9%) included an explicit uncertainty quantification method (Supporting Information, Table S8). The approaches used for applicability domain were mostly standard, based on descriptor ranges, leverage, or distance to the training set. The few uncertainty-aware studies relied on methods such as Gaussian process posterior uncertainty, Monte Carlo dropout, or ensemble-based confidence estimates.

Although these methods are not yet commonplace, their appearance in several recent studies (particularly those presenting prediction platforms) suggests growing recognition of prediction reliability as important for practical use. The rarity of these methods remains an important limitation in the context of regulatory science, where confidence in individual predictions and transparency about when a model is applied outside its reliable domain are directly relevant to interpretability and acceptance. Future work should therefore place greater emphasis on integrating applicability domain assessment and uncertainty quantification into cardiac ion channel models intended for safety-relevant use.

## 8 Conclusions

This review surveyed 155 QSAR and machine learning studies addressing cardiac ion channel prediction over 25 years (2001–mid-2026). The central finding is a historical misalignment between computational practice and multi-channel regulatory science that is now beginning to close.

The near-exclusive focus on *hERG* persisted far longer than data availability justified. This persistence reflects regulatory incentives, research inertia, and community infrastructure rather than a response to the full range of scientific needs. The CiPA initiative articulated the need for multi-channel assessment in 2013; computational research took nearly a decade to respond on a larger scale.

Since 2022, multi-channel modeling has expanded rapidly, with *hERG*, *Na*_*v*_ *1.5*, and *Ca*_*v*_ *1.2* now modeled jointly by multiple groups. Two secondary channels (*K*_*v*_ *4.3* and *Kir2.1*) remain entirely unmodeled in the surveyed literature, while models for *K*_*v*_ *7.1* remain scarce. Broader searches beyond ChEMBL confirm that public data for these channels exist but are too sparse and heterogeneous to support reliable modeling.

The most immediate priority is to consolidate primary-channel modeling by establishing the standardized benchmarks, shared datasets, and evaluation protocols that the field has so far lacked. For the secondary channels, progress will additionally depend on data release or federated learning and on methods suited to small datasets. Across both tiers, greater emphasis on prediction reliability and continued integration with physiological models is needed. The methodological foundations exist, but translating them into a comprehensive multi-channel modeling ecosystem depends on community coordination, particularly around data sharing.

## 9 Limitations

This Review is based on literature searches in Google Scholar, Scopus, and Web of Science, supplemented by backward and forward citation tracking. Despite these efforts, relevant studies may have been missed, particularly those reported in non-English publications, conference proceedings, or proprietary pharmaceutical research. The apparent scarcity of multi-channel modeling may therefore partly reflect publication bias or nonpublic industrial activity rather than a true absence of work.

The scope was limited to ligand-based predictive modeling studies, including QSAR, ML, and relevant pharmacophore- or other 3D ligand-based approaches. Purely structure-based methods, such as molecular docking and molecular dynamics studies not coupled to a predictive ligand- or pose-based model, were excluded. Relevant structure-based studies of cardiac ion channel safety may, therefore, fall outside the present survey.

Although we performed a quantitative analysis of the reported model performance, the conclusions that can be drawn from it are limited by the heterogeneity of the underlying literature. Studies differ in dataset composition, endpoint definition, activity thresholds, modeling setup, and evaluation strategy, and the field lacks standardized benchmarks. As a result, cross-study statistical comparisons are informative mainly at a descriptive level and should not be interpreted as controlled assessments of the methodological superiority.

Finally, the survey necessarily reflects what the authors reported in sufficient detail. Incomplete reporting of dataset construction, assay conditions, validation strategy, or model specification may have affected both study classification and retrospective comparison.

## Supplementary Material



## Data Availability

All data and code required to reproduce the results of this study are publicly available at https://github.com/M-Iwan/CardioQSAR-Review. The repository includes the supporting tables in machine-readable format, the processed datasets and derived results, the source code, and the Jupyter notebooks used for the analyses and figures. The repository is released under the Creative Commons Attribution 4.0 International (CC BY 4.0) license. A citable archived snapshot of the repository is available via Zenodo at 10.5281/zenodo.22272567.

## Notes

Summary of surveyed studies on cardiac ion channels (Table S1); publicly available datasets for cardiac ion channels (Table S2); summary of data splits and performance metrics for the best trained classification and regression models (Tables S3 and S4); Mann–Whitney *U* test results for classification and regression (Tables S5 and S6); data availability for the secondary channels (Table S7); summary of applicability domain and uncertainty quantification methods (Table S8); data, code, model, and prediction platform availability for surveyed studies (Table S9); ChEMBL data extraction and curation (Note S1); PubChem data extraction and curation (Note S2); BindingDB data extraction and curation (Note S3) (PDF)
