# Supplementary material for: 25 Years of Cardiac Ion Channel QSAR: From hERG Dominance to Multi-Channel Modeling
Source: J Chem Inf Model. 2026 Sep 17;66(18):11605–18. doi: 10.1021/acs.jcim.6c03072 (PMC13625950; doi:10.1021/acs.jcim.6c03072)
Supplement: Supplementary file 1 [file ci6c03072_si_001.pdf]

SUPPORTING INFORMATION

25 Years of Cardiac Ion Channel QSAR: From hERG Dominance to Multi-Channel Modelling

Mateusz Iwan[a,b,*], **Francesca Grisoni**[a], **Anastasia Pentina**[b], **Marina Garcia de Lomana**[b], **Alessandra Roncaglioni**[c]

[a] Institute for Complex Molecular Systems (ICMS), Department of Biomedical Engineering, Eindhoven University of Technology, P.O. Box 513, 5600 MB Eindhoven, The Netherlands

[b] Machine Learning Research, Research and Development, Bayer AG, Müllerstr. 178, 13353 Berlin, Germany

[c] Department of Environmental Health Sciences, Istituto di Ricerche Farmacologiche Mario Negri IRCCS, Via Mario Negri 2, Milan 20156, Italy

[*] Corresponding author. Email: m.iwan@tue.nl, ORCID: 0000-0001-5151-4659

Table of Contents

- **Table S1.** Summary of surveyed studies on cardiac ion channels.
- **Table S2.** Publicly available datasets for cardiac ion channels.
- **Table S3.** Summary of data splits and performance metrics obtained for the best trained models for classification.
- **Table S4.** Summary of data splits and performance metrics obtained for the best trained models for regression.
- **Table S5.** Mann-Whitney U tests results for classification.
- **Table S6.** Mann-Whitney U tests results for regression.
- **Table S7.** Data availability for the secondary channels.
- **Table S8.** Overview of applicability domain and uncertainty quantification methods used in the surveyed studies.
- **Table S9.** Data, code, model, and prediction platform availability for surveyed studies.
- **Note S1.** ChEMBL data extraction and curation.
- **Note S2.** PubChem data extraction and curation.
- **Note S3.** BindingDB data extraction and curation.

1 Supporting Tables

1.1 Studies

Table S1. Summary of surveyed studies on cardiac ion channels.

Study (Year)	Study type	Models	Features	Channel
Cavalli (2002) [1]	PH, Reg	PLS	3D Shape-based	*hERG*
Ekins (2002) [2]	PH	Alignment-based	3D Shape-based (CATALYST)	*hERG*
Roche (2002) [3]	BC	SOM, PCA, PLS, NN	MD (VolSurf, DRAGON)	*hERG*
Ekins (2003) [4]	PH	Alignment-based	3D Shape-based (CATALYST)	*hERG*
Keserü (2003) [5]	Reg	LR + hQSAR	MD (VolSurf, Sybyl)	*hERG*
Pearlstein (2003) [6]	PH	3D Shape-based	Pharmacophore	*hERG*
Aptula (2004) [7]	Reg	MLR	MD, Quantum Chemical	*hERG*
Aronov (2004) [8]	BC	DT	Pharmacophore-based	*hERG*
Bains (2004) [9]	BC	DT	MD, Fragment-based	*hERG*
Fioravanzo (2004) [10]	BC	PLS	MD (DRAGON), EVA (from IR and Raman spectra), DRAGON	*hERG*
Cianchetta (2005) [11]	Reg	PLS + hQSAR	3D Shape-based	*hERG*
O'Brien (2005) [12]	BC	NN, NB	Topological, Fragment-based, Circular	*hERG*
Tobita (2005) [13]	BC	SVM	MD (MOE), MACCS	*hERG*
Aronov (2006) [14]	PH	Alignment-based	3D Shape-based (MOE)	*hERG*
Coi (2006) [15]	Reg	LR	MD (CODESSA)	*hERG*
Dubus (2006) [16]	MC	DT	MD (MOE)	*hERG*
Ekins (2006) [17]	Reg	DT, SOM, Sammon Maps	MD (Smart Mining)	*hERG*
Gepp (2006) [18]	BC	DT	MD, QC (VAMP), Custom SMARTS	*hERG*
Seierstad (2006) [19]	Reg	MLP	MD (MOE), Topological (KH, GC), Atom Pairs, Fragment (ISIDA)	*hERG*
Sun (2006) [20]	BC	NB	Atom Types, Circular	*hERG*
Song (2006) [21]	Reg	SVM, PLS, RF	Fragment (Clark 2005)	*hERG*
Yoshida (2006) [22]	Reg	LR	MD	*hERG*
Du (2007) [23]	Reg	PLS	Docking scores	*hERG*
Leong (2007) [24]	Reg	SVM	Pharmacophore	*hERG*
Obrezanova (2007) [25]	Reg	GP	MD (in-house), custom SMARTS	*hERG*
Chekmarev (2008) [26]	BC	kNN, SVM, SOM	3D Shape-based	*hERG*
Gunturi (2008) [27]	Reg	kNN, LOESS	MD (BioSuite, SYBYL)	*hERG*

Table S1 – continued from previous page

Study (Year)	Study type	Models	Features	Channel
Jia (2008)[28]	BC	SVM	Atom Types	*hERG*
Kramer (2008)[29]	Reg	MLR, PLS, SVR	MD and QC	*hERG*
Li (2008)[30]	BC	SVM	Field-based	*hERG*
Thai$^{\diamond}$(2008)[31]	BC	NB	MD	*hERG*
Thai$^{\diamond}$(2008)[32]	BC, Reg	PLS, CP-NN	MD (MOE)	*hERG*
Wang (2008)[33]	BC	DT, MLP, RBFNetwork, SVM, LR	3D MD, QC	*hERG*
Coi (2009)[34]	Reg	MLR	MD (CODESSA), QC (MOPAC)	*hERG*
Ermondi (2009)[35]	Reg	PLS	Field-based	*hERG*
Hansen (2009)[36]	Reg	RF, SVR, GP, RidgeReg	MD (MOE), Field-based, Pharmacophore	*hERG*
Nisius$^{\diamond}$(2009)[37]	BC	SVM	Fragment	*hERG*
Nisius$^{\diamond}$(2009)[38]	BC	kNN	Circular, Fragment, 3D-shape based	*hERG*
Thai (2009)[39]	BC	PLS, NB, CP-NN	MD (MOE, VolSurf), SIBAR	*hERG*
Doddareddy (2010)[40]	BC	LDA, SVM	Circular	*hERG*
Obrezanova (2010)[41]	BC	GP, DT, SVM, RF, PLS	MD (in-house), custom SMARTS	*hERG*
Su (2010)[42]	BC, Reg	PLS	4D-FP (MDDM), MD (MOE)	*hERG*
Wiśniowska (2010)[43]	BC	RF	MD, Circular, Experimental	*hERG*
Cuny (2011)[44]	BC, Reg	kNN, PLS	MD (MOE), Pharmacophore	*hERG*
Kim (2011)[45]	BC	NB, RF	MD, Circular	*hERG*
Obiol-Pardo (2011)[46]	Reg	PLS	Field-based, Docking Scores	*hERG*, $K_v 7.1$
Robinson (2011)[47]	BC	SVM, RF	MD (MOE), Circular	*hERG*
Sinha (2011)[48]	Reg	MLR	MD	*hERG*
Shen (2011)[49]	BC	SVM	4D-FP (MDDM), MD (MOE, VolSurf)	*hERG*
Broccatelli (2012)[50]	BC	PLS, LDA, SVM, RF, kNN	MD (MOE, DRAGON, CDK), Field-based (VS+), Interaction (FLAP), Fragment	*hERG*
Kar (2012)[51]	BC, Reg	LDA, PLS	MD (PaDEL, Cerius2, DRAGON)	*hERG*
Polak (2012)[52]	Reg	MLP, NFS, SMO, RF	MD (ChemAxon)	$K_v 7.1$
Tan (2012)[53]	BC, Reg	PLS	MD (CODESSA), QC (MOPAC), Pharmacophore	*hERG*
Wang (2012)[54]	BC	NB	Circular, Path-based	*hERG*
Wiśniowska (2012)[55]	Reg	MLP, MON-MLP, NFS	MD (ChemAxon)	$Ca_v 1.2$
Czodrowski (2013)[56]	BC	RF	MD (RDKit)	*hERG*
Kireeva (2013)[57]	BC	GTM, SVM	Fragment (IPLF), Path-based (CDK)	*hERG*
Braga (2014)[58]	BC	SVM, RF, GBM, DT	Circular, Fragment (MACCS, PubChem), Pharmacophore	*hERG*

Table S1 – continued from previous page

Study (Year)	Study type	Models	Features	Channel
Kratz (2014)[59]	BC	Alignment-based	Pharmacophore (LigandScout, StudioCatalyst)	*hERG*
Liu (2014)[60]	BC	NB	MD, Circular	*hERG*
Braga (2015)[61]	BC, MC	SVM	Circular, Path-based (CDK)	*hERG*
Du (2015)[62]	BC	Winnow, SVM	MD, Circular	*hERG*
Ma (2015)[63]	Reg	RF, SVM, DNN, GBM, GP	MD, Circular, Fragment (MACCS), AtomPairs	*hERG*, $Na_v1.5$, $Ca_v1.2$
Chavan (2016)[64]	BC	kNN	Path-based (CDK), Topological (EState), Fragment (MACCS, PubChem, SubFP)	*hERG*
Didziapetris (2016)[65]	BC	GBM	Topological, Physicochemical	*hERG*
Gobbi (2016)[66]	Reg	LR	String-fragment	*hERG*
Sheridan (2016)[67]	Reg	XGB	Atom Pairs, Donor-Acceptor Pairs	*hERG*, $Na_v1.5$, $Ca_v1.2$
Wang (2016)[68]	BC	NB, SVM	Pharmacophore (DS2.5)	*hERG*
Zhang (2016)[69]	BC	SVM, NB, kNN, RF, DT	MD (PaDEL), Path-based (CDK), Fragment (MACCS, PubChem, SubFP)	*hERG*
Chemi (2017)[70]	PH, Reg	PLS	Pharmacophore	*hERG*
Li (2017)[71]	BC	kNN, SVM, MLR, PLS, RF, DT	MD (DRAGON, ChemAxon), Path-based (CDK), Topological (EState), Fragment (ISIDA, SA, GSFrag, SiRMS), 3D (Inductive, Mera and Mersy, Adriana, Spectrophores), String-based (QNPR)	*hERG*
Sun (2017)[72]	BC	SVC	Atom Types	*hERG*
Alves (2018)[73]	BC	kNN	MD (PaDEL, DRAGON), Circular, Fragment (SiRMS)	*hERG*
Munawar (2018)[74]	Reg	PLS	Field-based (GRIND)	*hERG*
Siramshetty (2018)[75]	BC	kNN, SVM, RF	Circular, Fragment (MACCS, PubChem)	*hERG*
Wacker (2018)[76]	BC, Reg	XGB, RF	MD (RDKit), Pharmacophore	*hERG*
Yang (2018)[77]	N/A	RF, SVM, kNN	Circular, Fragment (MACCS), Atom Pairs	*hERG*
Cai (2019)[78]	BC	DNN, NB, SVM, RF, GCN	MD (MOE), Embedding (Mol2Vec)	*hERG*
Hu (2019)[79]	BC	GCN, RF	Circular, Graphs	*hERG*
Konda (2019)[80]	BC	RF, MLP, SMO	MD (PaDEL)	*hERG*
Lee (2019)[81]	BC	LR, LogReg, Ridge, NN, NB, RF	MD (DRAGON), Circular	*hERG*
Munawar (2019)[82]	Reg	PLS	Interaction-based (PLIF)	*hERG*
Negami (2019)[83]	Reg	LR	FEP-based (MP-CAFEE)	*hERG*
Ogura (2019)[84]	BC	SVM	MD (MOE, PipelinePilot), Circular	*hERG*
Zhang (2019)[85]	BC	RF, DNN	MD (ChemoPy, MOE, PaDEL)	*hERG*
Choi (2020)[86]	BC	ANN, NB, SVM, RF	Circular, Path-based (CDK)	*hERG*

Table S1 – continued from previous page

Study (Year)	Study type	Models	Features	Channel
Khalifa (2020) [87]	BC, Reg	BayesNet, NB, MLP, K-Star, RF, SVM, SGD, SMO, GP, GLM, GBM, FFNN	Path-based (CDK), Topological (EState), Fragment (MACCS, PubChem, SubFP, Klek), Atom Pairs	$Na_v 1.5$
Kim (2020) [88]	BC	Self-attention DNN	Circular	*hERG*
Liu (2020) [89]	BC	SVM, RF, XGB	Atom Pairs, Topological (EState), Fragment (MACCS, Klek, PubChem), Path-based (FP4, FP4C), MD (PaDEL)	*hERG*
Ryu (2020) [90]	BC	Ensemble: DNN, GCN	MD (Mordred), Circular, Fragment (PubChem), Graphs	*hERG*
Siramshetty (2020) [91]	BC	RF, XGB, FFNN, LSTM	MD (RDKit), Circular, Embedding (AE)	*hERG*
Wang (2020) [92]	BC	Conv-CapsNet, RBM	MD, Fragment (MACCS)	*hERG*
Creanza (2021) [93]	BC	LASSO-SVM	Docking Scores (GLIDE, GOLD), Interactions (PLIF)	*hERG*
Karim (2021) [94]	BC	(DNN + GCN + Conv1D) > DNN	MD (Mordred), Circular, Embeddings (Smi2Vec, FP2Vec), Fragment (PubChem), Graph	*hERG*
Lee (2021) [95]	BC, Reg	XGB, RF	MD (RDKit), Pharmacophore	*hERG*
Meng (2021) [96]	Reg	SVR, RF, kNN, MLP, BRR	Interaction, Circular, Fragment (MACCS), Topological (EState)	*hERG*
Moorthy (2021) [97]	BC	DT, RF, SVM, NB	MD (MOE), Fragment (MACCS)	*hERG*
Sato (2021) [98]	Reg	SVM, RF, MLP	MD (RDKit, Mordred), Path-based (CDK), Circular	*hERG*
Stergiopoulos (2021) [99]	Reg	Stepwise Regression	MD, Experimental ($\alpha - 1 - acid - GP$ binding, IAM permeability)	*hERG*
Wu (2021) [100]	BC	MT-GAT	Graphs	*hERG*
Xiong (2021) [101]	BC	MT-GAT	Graphs	*hERG*
Arab (2022) [102]	MC	SVM, RF, MLP	MD (PaDEL)	*hERG*, $Na_v 1.5$
Delre (2022) [103]	BC	RF, KNN, GBM, XGB, SVM	MD (DRAGON)	*hERG*
Ding (2022) [104]	Reg	GBM, RF, SVM, kNN	MD, Circular, 3D Shape-based, 4D Dynamics-based	*hERG*
Ishihara (2022) [105]	BC	XGB, NB, SVM, RF, ANN	MD (RDKit, Mordred)	*hERG*
Kim (2022) [106]	BC	D-MPNN > Attn. Pool > DNN	Graphs	*hERG*
Kong (2022) [107]	BC	LogReg, SVM, NB, MLP, RF	Path-based (CDK), Topological (EState), Fragment (MACCS, PubChem)	$Na_v 1.5$
Krishna (2022) [108]	BC	DT, NN, SVM, RF, LDA, DNN	MD (RDKit, OPERA)	*hERG*
Lanevskij (2022) [109]	BC, Reg	XGB (with AFT)	MD	*hERG*
Melnikov (2022) [110]	Reg	XGB	MD (RDKit), Circular, Fragment (MACCS), Topological Torsion	*hERG*

Table S1 – continued from previous page

Study (Year)	Study type	Models	Features	Channel
Shan (2022) [111]	BC	D-MPNN	MD (RDKit, MOE), Circular, Fragment (MACCS, PubChem), Embedding (Mol2Vec)	*hERG*
Zhang (2022) [112]	BC	FFNN, RF, XGB	Circular, Fragment (MACCS)	*hERG*
Arab (2023) [113]	BC	(DNN + GCN) > DNN	MD (Mordred), Circular, Fragment (PubChem), Graphs	*hERG*, $Na_v1.5$, $Ca_v1.2$
Banerjee (2023) [114]	BC, Reg	PLS RASAR, Ridge, SVM, RF, GBM, ADA, MLP, kNN	MD, Similarity (RASAR)	*hERG*
Chen (2023) [115]	BC	Graph, kNN, SVM, RF, NN	MD (RDKit), Circular, Atom Pairs, Fragment (MACCS), Topological Torsion	*hERG*
Das (2023) [116]	BC, Reg	DT, RF, LogReg, AdaBoost, kNN, SVm, NB, NN, SGD	MD (PaDEL), Path-based (CDK)	*hERG*
Feng (2023) [117]	BC	DT, DNN	3D Shape-based, Embedding (CDDD, Transformer)	*hERG*
Wang$^{\diamond}$(2023) [118]	BC	(DNN + GAT) > DNN	Path-based (CDK), Circular, Fragment (PubChem), Atom Pairs, SMILES	*hERG*
Wang$^{\diamond}$(2023) [119]	BC	GCN, GSTN, SVM	Circular, Graphs	*hERG*
Vittorio (2023) [120]	BC	RF	MD, Circular	*hERG*
Ylipää (2023) [121]	BC	RF, XGB, SVM, DNN, GRU-DNN, MP-GNN	MD (Mordred), Graphs, SMILES	*hERG*
Arab (2024) [122]	BC	RF	MD (Mordred), Circular, Fragment (PubChem)	*hERG*, $Na_v1.5$, $Ca_v1.2$
Chen (2024) [123]	BC	DNN	Fragment (MACCS)	*hERG*, $Na_v1.5$, $Ca_v1.2$
He (2024) [124]	BC	RoBERTa emb. > MLP	SMILES	*hERG*
Liu (2024) [125]	BC	kNN, SVM, RF, MLP, LSTM, AE	MD (Mold2)	*hERG*
Sanches (2024) [126]	BC, MC, Reg	RF, kNN, SVM, LGBM, XGB	Circular	*hERG*
Wang (2024) [127]	BC	(GIN + DNN) > Attention > DNN	Circular, Fragment (MACCS, PubChem), Graphs, Embedding (Smi2Vec), Pharmacophore	*hERG*, $Na_v1.5$, $Ca_v1.2$
Yang (2024) [128]	BC	GNN + Attn	Graphs	*hERG*
Cano (2025) [129]	BC	XGB Ensemble	MD (MOE, alvaDesc), Topological (Kappa), Circular, Fragment (MACCS)	*hERG*
Feng (2025) [130]	BC	(GIN + BiGRU + MLP) > Attn	Circular, Fragment (PubChem, MACCS), Graphs, SMILES, Pharmacophore	*hERG*, $Na_v1.5$, $Ca_v1.2$
Gambacorta (2025) [131]	BC	RF, SVM, XGB, kNN, ADA	Fragment (CSFP)	ERG, $Na_v1.5$, $Ca_v1.2$
Han (2025) [132]	BC	40 models from HyperOPT-sklearn	Circular	*hERG*
Hossain (2025) [133]	BC	Logic Tensor Network	MD (Morgan), Path-based (CDK), Embeddings (MegaMolBART, LLaMA3.2, Gemini, QWEN1.5b)	*hERG*
Jin (2025) [134]	BC	((GNN > Transformer) + MLP) > Attn > MLP	MD, Circular, Fragment (MACCS), Graphs, Path-based (RDKit)	*hERG*

Table S1 – continued from previous page

Study (Year)	Study type	Models	Features	Channel
Jing (2025)[135]	BC	DNN, GNN, RF, GBM, SVM, NB, kNN, MLP	MD, Atom Types (AMBER), Circular, Fragment (MACCS), Atom Pairs, Topological Torsion	*hERG*
Kyro (2025)[136]	BC, Reg, Gen	(DNN + GAT) > DNN	MD (RDKit), Circular, Graphs, Embeddings (BDT)	*hERG*, $Na_v 1.5$, $Ca_v 1.2$
Lee (2025)[137]	BC	(GAT + MLP) > FCNN	MD, Circular, Graphs	*hERG*
Liu◊(2025)[138]	BC	NB, RF, SVM, kNN, XGB, Transformer	MD (RDKit), Circular, Fragment (MACCS), Atom Pairs, Path-based (FP2)	*hERG*
Liu◊(2025)[139]	BC, Reg	FCNN, Conv2D + DenseNet, EqGAT	Circular, Graphs, Images	*hERG*
Mohammad (2025)[140]	BC	RF, XGB, MLP, SVM, kNN, DT	MD (RDKit, Avalon), Path-based (CDK), Embeddings (Mol2Vec), Fragment (MACCS, Klek), Atom Pair	*hERG*
Tran-Nguyen (2025)[141]	BC	RF, XGB, DNN	Interaction (PLEC)	*hERG*
Xu (2025)[142]	BC	XGB	Embeddings (GROVER, kBERT, GIN, Chemprop), Various (RDKit, DeepChem, MolMap, Mordred)	*hERG*
Yang (2025)[143]	BC	RF, SVM, GBM, LogReg	Circular	*hERG*
Yu (2025)[144]	Reg	XGB, kNN, DNN, Ridge, Lasso	MD (AlvaDesc), Circular	*hERG*
Zhang (2025)[145]	Reg	Ridge, Lasso, RF, XGB, kNN, GNN	MD (Avalon), Circular, Fragment (MACCS), Graphs	*hERG*
Agarwal (2026)[146]	BC	GCN, GraphSAGE, GIN, GAT, MPNN, AttentiveFP	Graph	*hERG*, $Na_v 1.5$, $Ca_v 1.2$
Beidja (2026)[147]	BC	(Transformer + DNN + LSGAT) > DNN	SMILES, Graphs, Circular, Atom Pairs, Fragment (PubChem, Klek), Topological Torsion	*hERG*
Chu (2026)[148]	BC	(ViT + (GIN > GAT) + AE) > MoE Attention > DNN	Images, Graphs, Circular, Pharmacophore	*hERG*
Enokiya (2026)[149]	BC	GINE, GCN, GraphSAGE, GATv2	Graphs, MD (Mordred, RDKit), FAERS signals	*hERG*
Jovanocić (2026)[150]	BC, Reg	3xDNN > Attn > DNN	Embeddings (ChemBERTa, L1000), Circular, Atom Pair, Topological Torsion, MD (RDKit, MolGpKa)	*hERG*, $Na_v 1.5$, $Ca_v 1.2$, $K_v 7.1$
Liang (2026)[151]	BC	(GNN + BiGRU) > DNN	Graphs, Circular, Fragment (MACCS), MD (RDKit)	*hERG*, $Na_v 1.5$, $Ca_v 1.2$
Su (2026)[152]	BC	(MPNN + DNN) > DNN	Fragment (MACCS, PubChem, SubFP), Pharmacophore	*hERG*
Sun (2026)[153]	BC, Reg	SVM	Atom Types	*hERG*
Zhang (2026)[154]	BC	(GNN + DNN) > DNN, kNN, RF, XGB	Graphs, Circular, Pharmacophore	*hERG*
Zhao (2026)[155]	BC	RF, GBM, SVM, MPNN, GCN, CNN, Transformer	MD (Mordred), Circular, Fragment (MACCS, PubChem), Graphs, SMILES, Interaction (PLIF)	*hERG*

Annotations: ◊separate studies

Study type abbreviations: BC—Binary Classification, MC—Multi-Class Classification, Reg—Regression, PH—Pharmacophore, Gen—Generative modelling

Table S1 – continued from previous page

Study (Year)	Study type	Models	Features	Channel

Model abbreviations: ADA—ADaptive Boosting, AE—Auto Encoder, AFT—Accelerated Failure Time, BDT—BiDirectional Transformer, BRR—Bayesian Ridge Regression, CPNN—CounterPropagation Neural Network, DNN—Deep neural Network, DT—Decision Tree, FCNN—Fully Connected Neural Network, FFNN—FeedForward Neural Network, GAT—Graph Attention Transformer, GBM—Gradient Boosting Machine, GCN—Graph Convolution Network, GIN—Graph Isomorphism Network, GLM—Generalized Linear Model, GP—Gaussian Process, GRU—Gated Recurrent Unit, GSTN—Graph Substructure Transformer Network, GTM—Generative Topological Maps, hQSAR—hologram QSAR, kNN—k-Nearest Neighbors, LDA—Linear Discriminant Analysis, LGBM—Light Gradient Boosting Machine, LR—Linear Regression, LSTM—Long-Short Term Memory, MLP—Multi-Layer Perceptron, MLR—Multiple Linear Regression, MoE—Mixture of Experts, MON-MLP—Monotone Multi-Layer Perceptron, MT—Multi Task, NB—Naive Bayes, NFS—Neuro-Fuzzy Systems, NN—Neural Network, PCA—Principal Component Analysis, PLS—Partial Least Squares, RBM—Restricted Boltzmann Machines, SGD—Stochastic Gradient Descent, SMO—Sequential Minimal Optimization, SOM—Self Organizing Maps (also known as Kohonen Maps), SVM—Support Vector Machines, ViT—Vision Transformer, XGB—eXtreme Gradient Boosting

Features abbreviations: CDDD—Continuous and Data-Driven Descriptors, CSFP—Core Substituent Fingerprints, EVA—EigenVAlue, FEP—Free Energy Perturbation, FLAP—Fingerprints for Ligands and Proteins, FP4—Fingerprint 4, GCAT—Gose-Crippen Atom Types, GRIND—GRid INdependent Descriptors, IAM—Immobilized Artificial Membrane, IPLF—ISIDA Property-Labelled Fragment descriptors, KH—Kier-Hall Indices, MACCS—Molecular ACCes System, MD—Molecular Descriptors, MDDM—Molecular Distance-Dependent Matrix, PLIF—Protein-Ligand Interaction Fingerprints, QC—Quantum Chemical descriptors, QNPR—Quantitative Name-Property Relationship, RASAR—Read-Across Structure-Activity Relationship, SA—Structural Alerts, SIBAR—SImilarity BAsed descRiptors, SiRMS—Simplex Representation of Molecular Structure

Software: Avalon, BioSuite, CDK, Cerius2, ChemAxon, CODESSA, DRAGON, ISIDA, Ligand Scout, MOE, Mold2, MOPAC, PaDEL, PipelinePilot, Smart Mining, Studio Catalyst, Sybyl, VolSurf

1.2 Datasets

Table S2. Overview of publicly available datasets used for cardiac ion channel QSAR modelling. The Size column indicates the total number of compounds available as of February 2026. Links to currently accessible resources are listed in Table S3. In the Sources column, *Literature* denotes primary experimental publications, whereas *Study* denotes previously published cardiac ion channel QSAR studies included in this review.

Study (Year)	Size	No. Toxic	Data Type	Availability	Sources
			hERG		
Ponti (2001)[156]	137	N/A	IC_{50}	In paper§†	Literature
Cavalli (2002)[1]	37	N/A	IC_{50}	In paper	Study[156], FenichelDB
Roche (2002)[3]	472	N/A	IC_{50}	In paper†	In-house Roche, known drugs
Ekins (2002)[2]	22	N/A	IC_{50}	In paper	In-house ElliLilly
Keserü (2003)[5]	68	N/A	IC_{50}	In paper	Study[3], FenichelDB
Pearlstein (2003)[6]	23	N/A	IC_{50}	In paper	In-house Aventis Pharma
Aronov (2004)[8]	414	85@40μM	Binary	Online	Studies[1,2], FenichelDB, Literature
Aptula (2004)[7]	19	N/A	IC_{50}	In paper	Study[6], Literature
Bains (2004)[9]	124	N/A	IC_{50}	In paper	Literature
Fioravanzo (2004)[10]	70	N/A	IC_{50}	In paper	Literature
Cianchetta (2005)[11]	882	546@10μM	IC_{50}	SI†	Studies[1,6], In-house Sanofi
Tobita (2005)[13]	73	N/A	IC_{50}	In paper	Literature
Coi (2006)[15]	82	N/A	IC_{50}	In paper	Study[9], Literature
Ekins (2006)[17]	134	N/A	IC_{50}	In paper	Study[2], Literature
Seierstad (2006)[19]	439	N/A	IC_{50}	In paper†	In-house J&J
Song (2006)[21]	90	N/A	IC_{50}	SI	Studies[1,2], In-house Locus, Literature
Sun (2006)[20]	1979	N/A	IC_{50}	In paper†	Study[5], In-house Roche
Yoshida (2006)[22]	104	N/A	IC_{50}	In paper	Studies[2,6], FenichelDB, Literature
Du (2007)[23]	56	N/A	IC_{50}	In paper	Literature
Leong (2007)[24]	39	N/A	IC_{50}	In paper	Studies[1–3,6,9,20], Literature
Obrezanova (2007)[25]	137	N/A	IC_{50}	SI	Studies[2,6], FenichelDB, Literature
Gunturi (2008)[27]	165	N/A	IC_{50}	In paper	Study[22], Literature
Kramer (2008)[29]	113	N/A	IC_{50}	SI	Studies[2,6], FenichelDB, PubChem, Literature
Li (2008)[30]	495	N/A	Binary	SI	Studies[1,2,5,6,8,9,11,13,19,21], FenichelDB, Literature
Thai◇(2008)[31]	313	197@10μM	IC_{50}	SI	Studies[2,3,5–7,9,11,21], FenichelDB, Literature
Thai◇(2008)[32]	285	195<10μM	IC_{50}	SI	Studies[2,3,5–13,16–18,20,21,30,31], FenichelDB, Literature
Coi (2009)[34]	155	N/A	IC_{50}	In paper	Studies[2,6], Literature

Table S2 – continued from previous page

Study (Year)	Size	No. Toxic	Data Type	Availability	Sources
Nisius⋄(2009)[37]	242	138@10μM	IC_{50}	SI	Studies[1,5,9,17,21,22,31], Literature
Nisius⋄(2009)[38]	275	160<10μM	IC_{50}	SI	Studies[1,5,9,17,21,22,29,31], Literature
Polak (2009)[157]	201	N/A	IC_{50}	In paper	Study[3], FenichelDB, QTDrugs
Thai (2009)[39]	307	N/A	Mixed	SI	Studies[2,3,5–7,9,11,21], FenichelDB, Literature
Doddareddy (2010)[40]	2644	1112<10μM	Binary	SI	Studies[13,15,25,30], Literature
Obrezanova (2010)[41]	168	117@10μM	Binary	SI	Study[25], Literature
Su (2010)[42]	250	N/A	IC_{50}	SI	Studies[21,22,30,31]
Cuny (2011)[44]	529	178	IC_{50}	In paper†	Studies[5,6,17,20–22,156], DrugBank, Literature
Obiol-Pardo (2011)[46]	355	N/A	IC_{50}	SI	Literature
Robinson (2011)[47]	220	N/A	IC_{50}	SI	Literature
Sinha (2011)[48]	157	N/A	IC_{50}	In paper	Studies[2,6,10,11,15,19,21,30]
Broccatelli (2012)[50]	1051	185<1μM	IC_{50}	SI	ChEMBL, Tox-Portal
Polak (2012)[52]	98	N/A	IC_{50}	In paper	Literature
Tan (2012)[53]	113	N/A	IC_{50}	In paper	Literature
Wang (2012)[54]	806	N/A	IC_{50}	From[68]	Studies[3,13,21,23,25,30,31,37,41,157], WOMBAT, Literature
Wiśniowska (2012)[55]	123	N/A	IC_{50}	In paper	Literature
Czodrowski (2013)[56]	4415	3047@10μM	Binary‡	SI	ChEMBL
Braga (2015)[61]	5984	2565<10μM	Binary‡	GitHub¶	ChEMBL
Du (2015)[62]	306895	N/A	%Inh‡	SI, Online	MLSMR collection
Chavan (2016)[64]	172	93@10μM	IC_{50}	SI	FenichelDB, OCHEM
Didziapetris (2016)[65]	6690	3908@10μM	IC_{50}	SI	Studies[40,50,157], ChEMBL, Literature
Wang (2016)[68]	806	N/A	IC_{50}	SI	Study[54]
Zhang (2016)[69]	1570	N/A	IC_{50}	SI	Studies[40,54], ChEMBL, hERGCentral
Chemi (2017)[70]	421	22<50nM	IC_{50}	SI	Literature
Sun (2017)[72]	3024	1008@30μM	IC_{50}	SI	qHTS Thalium Flux assay
Munawar (2018)[74]	207	N/A	IC_{50}	SI	Studies[29,157], ChEMBL, FenichelDB
Sato (2018)[158]	291219	9890@10μM	Binary	From[111,117,129]†	Databases[159–162]
Siramshetty (2018)[75]	5804	4096@10μM	Binary‡	GitHub	Studies[30,40,47,72], ChEMBL
Cai (2019)[78]	7889	4355@10μM	Binary‡	SI	Studies[40,65,68,70], ChEMBL
Konda (2019)[80]	8705	5286@10μM	IC_{50}	SI	ChEMBL
Zhang(2019)[85]	697	211@5μM	Binary	SI	Studies[30,64], PubChem
Choi (2020)[86]	5299	3735<1μM	IC_{50}	SI	ChEMBL, Literature

Table S2 – continued from previous page

Study (Year)	Size	No. Toxic	Data Type	Availability	Sources
Ryu (2020)[90]	14440	6632@10μM	N/A	From[87†]	Studies[31,40,65,74,78], ChEMBL, BindingDB, In-house
Siramshetty (2020)[91]	8984	2230	Binary	GitHub	ChEMBL, PubChem, In-house NCATS
Creanza (2021)[93]	8337	1308<1μM	IC_{50}	SI	ChEMBL
Lee (2021)[95]	3448<br>678	N/A	IC_{50}<br>K_i	GitHub	ChEMBL
Meng(2021)[96]	9215	N/A	IC_{50}	SI	ChEMBL
Moorthy (2021)[97]	136*	N/A	IC_{50}	SI	Literature
Stergiopoulos (2021)[99]	90	N/A	IC_{50}	In paper	Study[158]
Wu (2021)[100]	1565	955@10μM	Binary[‡]	GitHub	Literature
Arab (2022)[102]	8930	N/A	IC_{50}	GitHub	Study[80], ChEMBL, PubChem
Delre (2022)[103]	8755	N/A	IC_{50}	GitHub	ChEMBL
Ding (2022)[104]	7836	N/A	IC_{50}	SI	Studies[65,82], ChEMBL
Kim (2022)[106]	304045<br>14322	N/A<br>8488@10μM	IC_{50}<br>Binary	GitHub	Study[62]<br>Study[78], Databases[159,161,163]
Krishna (2022)[108]	8311	1970@1μM	IC_{50}	SI	ChEMBL, Tox21[164]
Lanevskij (2022)[109]	9311	4862@10μM	IC_{50}	SI	Study[65], Literature
Zhang (2022)[112]	12850	6907@10μM	Binary	Online	Study[72], ChEMBL, Literature
Arab (2023)[113]	22246	N/A	IC_{50}	Zenodo	Studies[40,65,80,82], Databases[159,161–163], U.S. Patents
Banerjee (2023)[114]	261	N/A	IC_{50}	SI	Studies[17,51,99]
Chen (2023)[115]	4556	N/A	IC_{50}	SI	Studies[40,69], ChEMBL
Das (2023)[116]	6766	4000@10μM	IC_{50}	SI	Studies[1,13,28,31,35,48]
Wang (2023)[118]	10355	4479@10μM	Binary	GitHub	Studies[80,82,83,89], ChEMBL
Vittorio (2023)[120]	12789	N/A	pK	Zenodo	ChEMBL, GOSTAR
Sanches (2024)[126]	7307<br>6142<br>4315	3731@10μM<br>N/A	Binary<br>4-class<br>IC_{50}	SI	ChEMBL
Yang (2024)[128]	14322	8488@10μM	Binary	GitHub	Study[106], ChEMBL, PubChem
Gambacorta (2025)[131]	10035	5042@10μM	IC_{50}	GitHub	ChEMBL
Han (2025)[132]	6744	3465@10μM	Binary	GitHub	ChEMBL
Hossain (2025)[133]	20409	9826@10μM	Binary	GitHub	Studies[68,94], Databases[159,161,163,165]
Lee (2025)[137]	23381	14183@10μM	Binary	GitHub	Studies[30,68,85,106], ChEMBL, PubChem
Tran-Nguyen (2025)[141]	299927<br>2340	1937@20μM<br>N/A	Binary<br>IC_{50}	GitHub	ChEMBL, PubChem
Xu (2025)[142]	116	53@10μM	Mixed	SI	Literature

Table S2 – continued from previous page

Study (Year)	Size	No. Toxic	Data Type	Availability	Sources
Yu (2025)[144]	10798<br>796	N/A	IC_{50}	Platform	Studies[30,65,68,82,83,89], ChEMBL, BindingDB<br>Studies[90,94,109]
Zhang (2025)[145]	13890	N/A	IC_{50}	GitHub	Study[108], ChEMBL
Agarwal (2026)[146]	34124	14444@10μM	Binary	GitHub	Studies[122,131], PubChem
Jovanović (2026)[150]	331127	11881@10μM	IC_{50}	GitHub	ChEMBL
Sun (2026)[153]	1333	N/A	IC_{50}	SI	ChEMBL
Zhang (2026)[154]	16543	6333	Binary	SI	Studies[78,108], ChEMBL, Tox21, Literature
Zhao (2026)[155]	15806	5965@1μM	Binary	GitHub	ChEMBL
			$Na_v 1.5$		
Khalifa (2020)[87]	2596<br>714	N/A	IC_{50}<br>Inhibition	SI	ChEMBL, BindingDB, In-house Jubilant BioSys
Arab (2022)[102]	1767	N/A	IC_{50}	GitHub	ChEMBL, PubChem
Arab (2023)[113]	2069	N/A	IC_{50}	Zenodo	Databases[159,161,163], U.S. Patents
Gambacorta (2025)[131]	1436	679@10μM	IC_{50}	GitHub	ChEMBL
Agarwal (2026)[146]	3217	1894@10μM	Binary	GitHub	Studies[122,131], PubChem
Jovanović (2026)[150]	3160	1240@10μM	IC_{50}	GitHub	ChEMBL
			$Ca_v 1.2$		
Arab (2023)[113]	802	N/A	IC_{50}	Zenodo	Databases[159,161,163], U.S. Patents
Gambacorta (2025)[131]	734	405@10μM	IC_{50}	GitHub	ChEMBL
Agarwal (2026)[146]	1564	958@10μM	Binary	GitHub	Studies[122,131], PubChem
Jovanović (2026)[150]	1138	548@10μM	IC_{50}	GitHub	ChEMBL
			$K_v 7.1$		
Obiol-Pardo (2011)[46]	162	N/A	IC_{50}	SI	Literature
Jovanović (2026)[150]	115	30@10μM	IC_{50}	GitHub	ChEMBL

References to databases and datasets: BindingDB[163], ChEMBL[159], DrugBank[166], FenichelDB[167], GOSTAR[160], hERGCentral[162], OCHEM[168], PubChem[161], QTDrugs[169], Tox-Portal[170], Tox21[164], WOMBAT[171]

Annotations: §includes mixtures, †not all values available, ‡multiple thresholds available, ¶original link deprecated, a link to an updated version is given.

1.3 Models performance

Table S3. Summary of data splits and performance metrics obtained for the best trained models for classification. The best model was selected as designated by the authors of each study. The most relevant results were included. ACC—Accuracy, BA—Balanced Accuracy, SEN—Sensitivity, SPE—Specificity, ROC AUC—Receiver Operating Characteristic—Area Under Curve, MCC—Mathew's Correlation Coefficient.

Study (Year)	Splitting/Evaluation	Model	Threshold [μM]	ACC	BA	SEN	SPE	ROC AUC	MCC
			hERG						
Roche (2002)[3]	Random CV	DL (MLP)	<1,>10	-	-	0.70	0.89	-	0.61
	Ext			-	-	0.71	0.93	-	0.66
Aronov (2004)[8]	Random CV	PH	-	0.82	-	0.71	0.85	-	-
	Ext			-	-	0.63	0.71	-	-
Bains (2004)[9]	Random	ML (DT)	1	0.89§	-	1.00	0.83	-	-
Fioravanzo (2004)[10]	D-Optimal	ST (PLS)	5	-	-	0.96	0.67	-	-
O'Brien (2005)[12]	Random	ML (Ensemble)	-	0.91	-	0.87	-	-	-
Tobita (2005)[13]	Random CV	ML (SVM)	40	0.95	-	0.97	0.87	-	-
			1	0.90	-	0.86	0.93	-	-
Dubus (2006)[16]	Random CV	ML (DT)	<1,>10	0.96	-	0.98	0.89	-	-
Gepp (2006)[18]	-	ML (DT)	-	0.80	-	0.93	0.83	-	-
Sun (2006)[20]	Ext[5]	ML (NB)	30	0.88	-	-	-	-	-
Chekmarev (2008)[26]	Random CV	ML (SVM)	<1,>10	0.78	-	0.73	0.74	-	-
Jia (2008)[28]	Random CV	ML (SVM)	30	0.85	-	0.84	0.87	0.77	-
	Ext[5]			0.94	-	-	-	-	-
Li (2008)[30]	LOO CV	ML (SVM)	40	-	-	-	-	-	0.40
	Ext[171]			0.72	-	-	-	-	-
Thai◇(2008)[31]	Random CV	ML (NB)	<1,>10	0.93	-	0.90	0.95	-	-
Thai◇(2008)[32]	Random	DL (CP-NN)	<1,<10,>10	0.82	-	-	-	-	-
	Diverse train			0.85	-	-	-	-	-
Wang (2008)[33]	Random CV	ML (SVM)	<1,<10,>10	0.95	-	0.92	0.98	-	-
	Ext	ML (LogReg)		0.78	-	0.91	0.63	-	-
Nisius◇(2009)[37]	Random CV	ML (SVM)	10	0.85	-	0.87	0.84	-	-
Nisius◇(2009)[38]	Distance	ML (kNN)	<1,<10,>10	0.90	-	0.83	0.93	-	-
Thai (2009)[39]	Random CV	DL (CP-NN)	<1,<10,>10	0.68	-	0.70	0.57	-	-
	Diverse			0.72	-	0.70	0.64	-	-
Doddareddy (2010)[40]	Random CV	ML (SVM)	<10,>30	0.88	-	0.83	0.92	-	-
Obrezanova (2010)[41]	Distance	ML (GP)	10	0.86	-	0.97	0.65	-	-

Table S3 – continued from previous page

Study (Year)	Splitting/Evaluation	Model	Threshold [μM]	ACC	BA	SEN	SPE	ROC AUC	MCC
Su (2010)[42]	Random	ML (MLR)	40	0.84	-	0.94	0.37	-	-
	Ext[161]			0.83	-	0.85	0.84	-	-
Wiśniowska (2010)[43]	Random CV	ML (RF)	10	0.85	-	0.88	0.92	0.92	-
	Ext			0.73	-	0.62	0.81	-	-
Cuny (2011)[44]	Distance	ML (kNN)	MT	0.82	-	0.83	0.80	-	-
Kim (2011)[45]	Random	ML (NB)	10	0.95	-	0.82	1.00	>0.90	-
	In-house			0.86	-	0.80	0.92	-	-
Robinson (2011)[47]	Random CV	ML (Winnow)	1	-	-	-	-	-	0.87
	Ext			-	-	-	-	-	0.40
Shen (2011)[49]	Random CV	ML (SVM)	10	0.95	-	0.90	0.96	-	-
	Ext			0.87	-	-	-	-	-
Broccatelli (2012)[50]	Random	ML (Ensemble)	<1,>16.6	0.90	-	0.93	0.89	-	-
	In-house			0.85	-	0.64	1.00	-	-
Kar (2012)[51]	Random	ST (LDA)	5.62	0.80	-	0.89	0.71	0.85	-
Tan (2012)[53]	Diverse	ML (Ensemble)	1	0.83	-	0.86	0.76	-	-
Wang (2012)[54]	Random	ML (NB)	5MT	0.85	-	-	-	-	-
Czodrowski (2013)[56]	Random CV	ML (RF)	1	0.97	-	0.14	-	0.56	0.24
			10	0.69	-	0.53	-	0.67	0.35
Kireeva (2013)[57]	Random Nested CV	ML (SVM)	10	-	0.81	-	-	-	-
Braga (2014)[58]	Random CV	ML (Ensemble)	10MT	0.90	0.91	0.89	0.93	0.91	0.76
Kratz (2014)[59]	N/A	PH	N/A	-	0.85	-	-	0.91	-
Liu (2014)[60]	Random CV	ML (NB)	<10,>30	0.91	-	0.90	0.92	-	-
	Ext[40]			0.58	-	0.61	0.57	-	-
Braga (2015)[61]	Random CV	ML (SVM)	10	0.83†	-	0.89†	0.79†	-	-
			<1,<10,>10	0.83	-	0.77	-	-	-
Chavan (2016)[64]	Random CV	ML (Ensemble)	5	0.70	-	0.78	0.61	-	-
	Ext[161]			0.55	-	0.63	0.54	-	-
Didziapetris (2016)[65]	Random	ML (GBM)	10	0.74	-	0.84	0.62	0.72	0.46
	Ext (Temporal)			0.78	-	0.75	0.82	0.83	0.68
Wang (2016)[68]	Diverse train	ML (SVM)	40	0.82	-	0.91	0.65	0.84	0.59
Zhang (2016)[69]	Random CV	ML (SVM)	30MT	0.84	-	0.96	0.38	0.83	-
Li (2017)[71]	Random CV	ML (Ensemble)	1	0.84	-	0.92	0.63	0.89	0.57
	Ext[40]			0.90	-	0.96	0.66	0.91	0.67
Sun (2017)[72]	Random CV	ML (SVM)	30	-	-	0.86	0.89	0.93	-
	Ext[5]			-	-	-	-	0.86	-
Alves (2018)[73]	Random CV	ML (Ensemble)	10	0.80	0.80	0.87	0.72	-	-

Table S3 – continued from previous page

Study (Year)	Splitting/Evaluation	Model	Threshold [μM]	ACC	BA	SEN	SPE	ROC AUC	MCC
Siramshetty (2018)[75]	Stratified Random CV	ML (RF)	<1,>10	-	0.87†	0.82†	0.92†	0.94	-
	Ext[40]	ML (SVM)	10	-	-	-	-	0.89†	-
Wacker (2018)[76]	Ext[172]	ML (XGB)	10	-	-	-	-	0.93	-
Cai (2019)[78]	Diverse train	DL (DNN)	<10,>MT	0.93	-	-	-	0.97	-
Konda (2019)[80]	Random CV	ML (RF)	10	0.81	-	0.70	0.88	-	-
	External	ML (Ensemble)	30	0.92	-	0.96	0.79	-	0.79
Lee (2019)[81]	Random CV	DL (NN)	10	0.90	-	0.97	0.32	0.76	0.37
	Ext			0.80	-	0.60	1.00	-	0.66
Ogura (2019)[84]	Random	ML (SVM)	10	0.98	0.83	0.67	0.99	0.96	-
Zhang (2019)[85]	Random	DL (NN	5	0.78	-	0.89	0.71	-	-
	Ext[161]		20% Inh@10uM	0.82	-	0.46	0.77	-	-
Choi (2020)[86]	Random	ML (RF)	<1,>10	0.90	-	-	-	0.95	-
	Ext			0.81	-	-	-	-	-
Kim (2020)[88]	Ext[65]	DL (DNN)	10	-	-	-	-	0.93	-
Liu (2020)[89]	Random CV	ML (Ensemble)	30	0.85	-	0.95	0.52	0.89	-
	Ext[162]		1,10	0.76	-	0.76	0.64	0.79	-
Ryu (2020)[90]	Stratified Random	DL (GCN, DNN)	10	0.81	-	0.93	0.69	-	0.64
	Ext[31,74]			0.77	-	0.83	0.64	-	0.48
Siramshetty (2020)[91]	Random CV	ML (RF)	<1,>10	-	0.84	0.73	0.95	-	-
	Scaffold CV			-	0.80	0.66	0.94	0.90	-
Wang (2020)[92]	Random CV[40]	DL (CapsNet)	<10,>30	0.92	-	0.92	0.93	0.94	0.84
	Ext[40]			0.79	-	0.94	0.71	0.81	0.60
Creanza (2021)[93]	Random CV	ML (SVM)	<10,>80	0.79	-	-	-	0.86	-
Karim (2021)[94]	Random CV	DL (MM-DNN)	10	0.86	-	0.86	0.86	0.93	0.72
	Ext[90]			0.81	-	0.79	0.83	-	0.60
	Ext[91]			0.75	-	0.70	0.79	-	0.22
Lee (2021)[95]	Random CV: binding	ML (XGB)	<1,>10	0.89	-	0.89	0.88	-	-
	Random CV: clamp			0.87	-	0.71	0.96	-	-
Moorthy (2021)[97]	Random CV	ML (SVM)	10	0.78	-	0.65	0.90	0.71	0.57
Wu (2021)[100]	Random	DL (GNN)	1	-	-	-	-	0.69	-
			5	-	-	-	-	0.63	-
			10	-	-	-	-	0.67	-
			30	-	-	-	-	0.65	-
Xiong (2021)[101]	Stratified Random	DL (GNN)	10	0.89	-	0.87	0.91	0.94	0.78
Arab (2022)[102]	Ext	ML (RF)	30	0.93	-	0.99	0.75	-	0.80

Table S3 – continued from previous page

Study (Year)	Splitting/Evaluation	Model	Threshold [μM]	ACC	BA	SEN	SPE	ROC AUC	MCC
Delre (2022) [103]	Temporal	ML (Consensus)	1	-	0.72	0.66	0.77	0.73	0.34
			10	-	0.72	0.67	0.76	0.75	0.43
Ishihara (2022) [105]	Random	ML (Ensemble)	10	0.87	-	0.90	0.83	0.94	0.73
Kim (2022) [106]	Scaffold Distance	DL (GNN)	10	0.74	0.74	0.80	0.67	0.81	0.48
	Ext [90]			0.84	0.85	0.83	0.86	0.84	0.66
	Ext [91]			0.80	0.78	0.73	0.82	0.85	0.52
Krishna (2022) [108]	Random CV	ML (RF)	-	0.95	0.93	0.88	0.97	-	0.86
	Ext [161]			0.93	0.77	0.98	0.55	-	0.64
Lanevskij (2022) [109]	Random	ML (XGB)	10	0.73	-	0.79	0.66	0.80	0.46
	Temporal		5	0.79	-	0.54	0.86	0.81	0.40
			10	0.73	-	0.70	0.75	0.80	0.43
			30	0.72	-	0.86	0.52	0.77	0.41
Melnikov (2022) [110]	Temporal	ML (XGB)	5	0.73	0.70	0.51	0.88	-	-
			10	0.77	0.79	0.69	0.90	-	-
			30	0.82	0.75	0.51	0.88	-	-
Shan (2022) [111]	Random CV [78]	DL (GNN)	10	-	-	-	-	0.96	-
	Scaffold CV [78]			-	-	-	-	0.92	-
	Ext [40]		<10,>30	0.90	-	0.90	0.91	0.96	-
	Ext [91]		30	0.80	-	0.81	0.80	0.86	-
Zhang (2022) [112]	Random CV [78]	ML (Ensemble)	10	0.84	-	0.82	0.86	0.91	0.68
	Ext [61]			0.81	-	0.87	0.77	0.89	0.63
	Ext [78]			0.98	-	0.99	0.97	1.00	0.92
	Ext [40]		<10,>30	0.92	-	0.92	0.92	0.96	-
Arab (2023) [113]	Distance	DL (DNN)	10	0.81	-	0.87	0.76	-	0.62
Banerjee (2023) [114]	Sorted Y-based	ML (RF)	-	0.78	-	0.73	-	0.82	0.55
Chen (2023) [115]	Random	ML (RF)	1	0.89	-	0.51	0.96	0.88	-
			10	0.80	-	0.79	0.82	0.88	-
		DL (GCN)	30	0.88	-	0.95	0.61	0.90	-
			<1,>10	0.89	-	0.80	0.93	0.93	-
		ML (SVM)	<10,>30	0.89	-	0.97	0.65	0.92	-
Das (2023) [116]	Random	ML (Many Tied)	10	1.00	-	1.00	1.00	1.00	1.00
Feng (2023) [117]	Ext [40]	DL (GNN)	<10,>30	0.92	-	0.92	0.93	0.98	0.84
	Ext [61]		10	0.81	-	0.80	0.83	0.89	0.63
	Ext [78]		10	1.00	-	1.00	1.00	1.00	0.99
Wang$^{\diamond}$ (2023) [118]	Random CV	DL (GNN)	10	0.82	-	0.78	0.85	-	0.63
Wang$^{\diamond}$ (2023) [119]	Random CV [93]	DL (GAT)	<10,>30	0.89†	-	0.89†	-	0.89†	-
Vittorio (2023) [120]	Random CV	ML (RF)	10	0.79	-	0.84	0.72	0.87	0.57
	Ext [40]	ML (Ensemble)		0.69	-	0.79	0.43	0.67	0.22

Table S3 – continued from previous page

Study (Year)	Splitting/Evaluation	Model	Threshold [μM]	ACC	BA	SEN	SPE	ROC AUC	MCC
Ylipää (2023)[121]	Random CV[84]	DL (GNN)	10	-	0.90	0.89	0.94	-	0.48
	Random[84]			-	0.90	0.88	0.94	-	0.47
Arab (2024)[122]	Distance	ML (RF)	10	0.82	-	0.92	0.70	-	0.64
Chen (2024)[123]	Random	DL (DNN)	10	0.82	-	0.83	0.81	0.89	-
He (2024)[124]	-	DL (CLM)	-	0.80	-	0.80	0.80	0.88	0.61
Liu (2024)[125]	Random CV[94]	ML/DL (Ensemble)	10	0.87	-	0.87	0.86	0.94	0.73
Sanches (2024)[126]	Random	ML (LightGBM)	10	-	0.87	0.85	0.86	0.82	0.52
		ML (RF)	<1,<10,>10	-	0.83	0.82	0.83	0.80	0.56
Wang (2024)[127]	Distance	DL (Multimodal)	10	0.83	-	0.86	0.79	-	0.66
Yang (2024)[128]	Scaffold Distance	DL (GNN)	10	0.75	0.74	0.81	0.68	0.82	0.49
	Ext[90]		10	0.66	0.66	0.67	0.64	0.71	0.29
	Ext[91]		10	0.72	0.70	0.66	0.74	0.80	0.36
Cano (2025)[129]	Random[84]	ML (Ensemble)	10	0.90	-	0.83	0.90	-	0.41
Feng (2025)[130]	Distance[113]	DL (Multimodal)	10	0.85	-	-	-	-	0.83
Gambacorta (2025)[131]	Random Nested CV	ML (RF)	10	0.76	-	0.74	0.79	0.84	0.53
Han (2025)[132]	Stratified Random	ML (RF)	10	0.80	-	0.80	0.80	0.88	0.60
	Ext[173]		10	0.79	-	0.77	0.80	0.87	0.57
Hossain (2025)[133]	Scaffold	DL (LTN)	10	0.93	0.93	0.93	0.93	-	0.85
	Ext[113]			0.83	0.83	0.78	0.90	-	0.66
Jin (2025)[134]	Random CV[128]	DL (Multimodal)	10	0.85	0.83	0.85	0.81	0.91	0.68
	Ext[90]			0.80	0.77	0.83	0.71	0.85	0.54
	Ext[91]			0.73	0.74	0.83	0.66	0.82	0.43
Jing (2025)[135]	Random	DL (GNN)	10	0.75	-	0.80	-	-	0.52
	Ext[90]			0.84	-	0.93	-	-	0.66
Kyro (2025)[136]	Distance[113]	DL (Multimodal)	10	0.84	-	0.86	0.80	-	0.67
Lee (2025)[137]	Random	DL (GNN,GRU)	10	0.87	-	-	-	0.91	-
	Ext[78]			0.84	-	-	-	0.87	-
	Ext[94]			0.96	-	-	-	0.98	-
Liu$^{\diamond}$(2025)[138]	Random	DL (Transformer)	10	0.85	0.84	0.87	0.81	0.93	0.68
	Ext			0.86	0.86	0.90	0.82	0.93	0.72
Liu$^{\diamond}$(2025)[139]	Random CV	DL (Multimodal)	10	0.93	-	-	-	0.94	-
Mohammad (2025)[140]	Diverse[93]	ML (XGB)	<10,>60	0.88	-	0.91	0.81	-	-
	Ext[144]	ML (RF)	10	0.84	-	0.84	0.86	-	-
Tran-Nguyen (2025)[141]	Scaffold	DL (DNN)	20	-	0.76	0.86	0.65	-	-
Xu (2025)[142]	Scaffold[94]	ML (XGB)	10	0.75	-	-	-	0.83	0.23
	Ext			0.76	-	0.79	0.73	-	0.52

Table S3 – continued from previous page

Study (Year)	Splitting/Evaluation	Model	Threshold [μM]	ACC	BA	SEN	SPE	ROC AUC	MCC
Yu (2025)[144]	Random CV	ML (XGB)	10	0.81	-	0.85	0.76	-	0.61
	Distance			0.73	-	0.80	0.62	-	0.41
Yang (2025)[143]	Random	ML (RF)	30	0.77	-	0.90	0.44	0.81	0.40
Agarwal (2026)[146]	Random	DL (AttentiveFP)	10	-	-	-	-	0.89	-
Beidja (2026)[147]	Random CV[118]	DL (Multimodal)	10	0.82	-	0.85	0.79	0.91	0.64
	Ext[90]			0.81	-	0.78	0.87	0.88	0.60
	Ext[91]			0.76	-	0.83	0.73	0.87	0.50
Chu (2026)[148]	Diverse[68]	DL (Multimodal)	40	-	-	-	-	0.90	-
Enokiya (2026)[149]	Stratified CV[161]	DL (GATv2)	-	-	-	-	-	0.84	-
Jovanović (2026)[150]	Distance	DL (Multimodal)	1	0.99	-	0.56	0.99	0.96	0.38
			10	0.97	-	0.64	0.98	0.92	0.47
Liang (2026)[151]	Random CV	DL (Multimodal)	10	0.84	-	-	-	0.93	0.69
	Stratified Random			0.85†	-	0.82†	0.88†	0.93†	0.70†
Su (2026)[152]	Random CV	DL (Multimodal)	10	0.81	-	0.84	0.78	0.89	0.61
	Ext[94]			0.71	-	0.91	0.63	0.83	0.48
				0.89	-	0.93	0.79	0.92	0.73
	Ext[75]			0.76	-	0.62	0.97	0.88	0.60
Sun (2026)[153]	Random CV	ML (SVM)	10	0.80	-	0.78	0.83	0.88	-
Zhang (2026)[154]	Random CV	DL (Multimodal)	10	-	0.88	0.84	0.91	0.95	0.76
	Scaffold			-	0.84	0.81	0.86	0.92	0.67
Zhao (2026)[155]	Distance	DL (Multimodal)	<1,>10	-	-	0.75	-	0.83	-
	Ext[113]		10	0.83	-	0.79	0.89	-	0.67
			Na_v1.5						
Khalifa (2020)[87]	Distance	ML (GBM)	1	0.93	-	0.99	0.78	-	0.82
			10	0.93	-	0.94	0.92	-	0.85
			30	0.94	-	0.93	0.95	-	0.83
Arab (2022)[102]	Random CV	ML (SVM)	30	0.87	-	0.88	0.85	-	0.71
Kong (2022)[107]	Distance	ML (RF)	30	0.93	-	0.90	0.96	0.95	0.86
Arab (2023)[113]	Distance	DL (DNN)	10	0.82	-	0.86	0.73	-	0.58
Arab (2024)[122]	Distance	ML (RF)	10	0.86	-	0.95	0.67	-	0.66
Chen (2024)[123]	Random	DL (DNN)	10	0.86	-	0.90	0.78	0.89	-
Wang (2024)[127]	Distance	DL (Multimodal)	10	0.86	-	-	-	0.92	-
Feng (2025)[130]	Distance[113]	DL (Multimodal)	10	0.87	-	-	-	-	0.79
Gambacorta (2025)[131]	Random Nested CV	ML (RF)	10	0.80	-	0.77	0.83	0.89	0.61
Kyro (2025)[136]	Distance[113]	DL (Multimodal)	10	0.89	-	0.96	0.76	-	0.75

Table S3 – continued from previous page

Study (Year)	Splitting/Evaluation	Model	Threshold [μM]	ACC	BA	SEN	SPE	ROC AUC	MCC
Agarwal (2026) [146]	Random	DL (AttentiveFP)	10	-	-	-	-	0.95	-
Jovanović (2026) [150]	Distance	DL (Multimodal)	10	0.76	-	0.70	0.78	0.83	0.45
Liang (2026) [151]	Random CV<br>Stratified Random	DL (Multimodal)	10	0.86<br>0.86†	-<br>-	-<br>0.89†	-<br>0.82†	0.92<br>0.94†	0.67<br>0.70†
			Ca_v1.2						
Arab (2023) [113]	Distance	DL (DNN)	10	0.86	-	0.96	0.69	-	0.70
Arab (2024) [122]	Distance	ML (RF)	10	0.91	-	0.98	0.79	-	0.81
Chen (2024) [123]	Random	DL (DNN)	10	0.86	-	0.87	0.85	0.94	-
Wang (2024) [127]	Distance	DL (Multimodal)	10	0.86	-	-	-	0.93	-
Feng (2025) [130]	Distance [113]	DL (Multimodal)	10	0.86	-	-	-	-	0.78
Gambacorta (2025) [131]	Random Nested CV	ML (XGB)	10	0.79	-	0.81	0.76	0.88	0.57
Kyro (2025) [136]	Distance [113]	DL (Multimodal)	10	0.91	-	0.96	0.83	-	0.81
Agarwal (2026) [146]	Random	DL (GIN)	10	-	-	-	-	0.93	-
Jovanović (2026) [150]	Distance	DL (Multimodal)	10	0.78	-	0.70	0.81	0.84	0.49
Liang (2026) [151]	Random CV<br>Stratified Random	DL (Multimodal)	10	0.87<br>0.87†	-<br>-	-<br>0.95†	-<br>0.80†	0.94<br>0.95†	0.69<br>0.72†
			K_v7.1						
Jovanović (2026) [150]	Distance	DL (Multimodal)	10	0.86	-	0.00	0.92	0.38	-0.08

Annotations: §—approximate value, †—estimated from a figure, MT—Multiple Thresholds

Abbreviations: CLM—Chemical Language Model, LTN—Logic Tensor Network

Table S4. Summary of data splits and performance metrics obtained for the best trained models for regression on test sets. The best model was selected as designated by the authors of each study. The most relevant results were included. R^2—Coefficient of determination, RMSE—Root Mean Squared Error, MAE—Mean Absolute Error.

Study (Year)	Splitting/Evaluation	Model	R^2	RMSE	MAE
		hERG			
Cavalli (2002)[1]	LOO CV<br>Manual	PH/ST (PLS)	0.77<br>0.74	-<br>-	-<br>-
Keserü (2003)[5]	Stratified Random	ST (MLR)	0.81	-	-
Pearlstein (2003)[6]	LOO CV	PH/ST (PLS)	0.57	-	-
Aptula (2004)[7]	Sorted Uniform	ST (MLR)	0.95‡	0.43‡	0.41‡
Coi (2006)[15]	Random	ST (MLR)	0.82	-	0.41
Ekins (2006)[17]	-	ML (DT)	0.33	-	-
Seierstad (2006)[19]	Random CV	DL (Ensemble)	0.51‡	0.85‡	0.71‡
Song (2006)[21]	Random	ML (SVM)	0.85	0.60	-
Yoshida (2006)[22]	Random CV	ST (MLR)	0.66	-	-
Du (2007)[23]	Random CV	ST (LR)	0.56	-	-
Leong (2007)[24]	Manual	PH/ML (SVM)	0.94	-	-
Obrezanova (2007)[25]	Sorted Uniform	ML (GP)	0.63	0.46	-
Gunturi (2008)[27]	Random CV[22]	ST (LLR)	0.82	-	-
Kramer (2008)[29]	Random	ML (SVM)	0.70	0.74	0.55
Thai◇(2008)[32]	Random<br>Distance	DL (CPNN)	0.87<br>0.87	-<br>-	-<br>-
Coi (2009)[34]	DISE	ST (MLR)	0.86	-	-
Ermondi (2009)[35]	LOO CV<br>Ext[1]	PH/ST (PLS)	0.69‡<br>0.19‡	0.37‡<br>0.90‡	-<br>0.81‡
Hansen (2009)[36]	LCO CV	ML (Ensemble)	0.54	0.57	-
Cuny (2011)[44]	Distance	ST (PLS)	0.59	-	-
Obiol-Pardo (2011)[46]	LOO CV<br>Ext	PH/ST (PLS)	0.46<br>-	0.92<br>0.89	-<br>-
Sinha (2011)[48]	Stratified Random	ST (MLR)	0.64	0.78	0.68
Kar (2012)[51]	Random<br>Ext	ST (GFA)<br>ST (PLS)	0.60<br>0.60	-<br>-	-<br>-
Tan (2012)[53]	Distance	ML (Ensemble)	0.75	-	0.45
Gobbi (2016)[66]	Random CV	ST (MLR)	0.93	-	-
Sheridan (2016)[67]	Temporal	-	-	-	-
Chemi (2017)[70]	Random CV<br>Ext	PH/ST (PLS)	0.80<br>0.86	-<br>-	-<br>-

Table S4 – continued from previous page

Study (Year)	Splitting/Evaluation	Model	R^2	RMSE	MAE
Munawar (2018)[74]	LOO CV	PH/ST (PLS)	0.63	0.84	-
	Diverse		0.60	-	-
Wacker (2018)[76]	Random CV	ML (XGB)	0.6§	-	-
	Ext[172]		0.68	0.71	0.52
Munawar (2019)[82]	Diverse	PH/ST (PLS)	0.58	-	-
	Ext		0.51	-	-
Lee (2021)[95]	Random CV: binding	ML (XGB)	0.76	0.58	-
	Random CV: clamp		0.80	0.59	-
Meng (2021)[96]	Random CV	ML (SVM)	-	0.59†	-
	Ext[80]		-	0.88	-
	Ext[82]		-	1.11	-
Sato (2021)[98]	Random CV	ML (SVM)	0.59	0.60	-
Stergiopoulos (2021)[99]	Random	ST (MLR)	0.72	-	-
Arab (2022)[102]	Ext	ML (RF)	0.67	0.77	-
Ding (2022)[104]	Random Nested CV	ML (Ensemble)	0.65	0.53	0.36
	Ext[80]		0.49	0.76	0.46
	Ext[83]		0.62	0.77	0.50
Lanevskij (2022)[109]	Random: patch-clamp	ML (XGB)	0.41§	0.55§	0.44§
	Random: all assays		0.29	0.63	0.49
Melnikov (2022)[110]	Temporal	ML (XGB)	0.46	0.59	0.47
Banerjee (2023)[114]	Sorted Y-based	ST (PLS)	-	-	0.55
Das (2023)[116]	-	ST (MLR)	-	0.47	0.38
Sanches (2024)[126]	Random	ML (SVM)	0.61	0.44	0.35
Kyro (2025)[136]	Distance[113]	DL (Multimodal)	0.44	0.74	0.51
Liu◇(2025)[139]	Random CV	DL (Multimodal)	0.68	0.45	-
Yu (2025)[144]	Random CV	ML (XGB)	0.62	0.60	0.38
	Distance		0.36	0.53	0.40
Zhang (2025)[145]	Random	ML (XGB)	0.78	-	-
Jovanović (2026)[150]	Distance	DL (Multimodal)	-	0.72	-
Sun (2026)[153]	Random CV	ML (SVM)	0.63	0.55	0.38
	Temporal		-	-	0.50
		Na_v1.5			
Khalifa (2020)[87]	Distance	ML (RF)	0.71	0.73	-
Arab (2022)[102]	Ext	ML (RF)	0.71	0.51	-
Kyro (2025)[136]	Distance[113]	DL (Multimodal)	0.32	0.62	0.43

Table S4 – continued from previous page

Study (Year)	Splitting/Evaluation	Model	R^2	RMSE	MAE
Jovanović (2026)[150]	Distance	DL (Multimodal)	-	0.57	-
		Ca_v 1.2			
Wiśniowska (2012)[55]	Random CV	DN (Ensemble)	0.29	1.10	-
Kyro (2025)[136]	Distance[113]	DL (Multimodal)	0.54	1.01	0.82
Jovanović (2026)[150]	Distance	DL (Multimodal)	-	0.82	-
		K_v 7.1			
Obiol-Pardo (2011)[46]	LOO CV	PH/ST (PLS)	0.41	0.99	-
	Ext		-	0.82	-
Polak (2012)[52]	Random CV	DL (Ensemble)	-	0.86	-

Annotations: §—approximate value, †—estimated from a figure, ‡—derived manually

Abbreviations: LCO CV—Leave-Cluster-Out Cross-Validation, LLR—Local Lazy Regression, LOO CV—Leave-One-Out Cross-Validation

1.4 Statistical analysis

Table S5. Pairwise Mann-Whitney U comparisons of classification performance metrics across era, model class, evaluation strategy, and classification threshold. For each comparison, group size (N), median value ($\tilde{x}$), rank-biserial correlation (r_{rb}), effect size, p value (p), adjusted p value (p_{adj}), and conclusion are reported. Values were corrected using Benjamini-Hochberg FDR correction on a per-metric basis. Conclusions drawn at $p_{adj} < 0.05$. Dashes indicate comparisons where insufficient group size (N<3) precludes testing. ACC—Accuracy, BA—Balanced Accuracy, SEN—Sensitivity, SPE—Specificity, ROC AUC—Receiver Operating Characteristic Area Under Curve, MCC—Matthew's Correlation Coefficient.

Metric	$Group_A$	N_A	$\tilde{x}_A$	$Group_B$	N_B	$\tilde{x}_B$	r_{bc}	Effect size	p	p_{adj}	Conclusion
						Era					
ACC	Expansion	77	0.83	Foundation	6	0.90	-0.42	Medium	0.086	0.22	Expansion ∼ Foundation
	Foundation	6	0.90	Consolidation	17	0.85	0.32	Medium	0.26	0.52	Foundation ∼ Consolidation
	Expansion	77	0.83	Consolidation	17	0.85	-0.06	Negligible	0.72	0.78	Expansion ∼ Consolidation
BA	Expansion	18	-	Foundation	0	-	-	-	-	-	-
	Expansion	18	-	Consolidation	2	-	-	-	-	-	-
	Foundation	0	-	Consolidation	2	-	-	-	-	-	-
SEN	Expansion	75	0.83	Foundation	8	0.86	-0.15	Small	0.49	0.90	Expansion ∼ Foundation
	Foundation	8	0.86	Consolidation	16	0.85	0.13	Small	0.62	0.90	Foundation ∼ Consolidation
	Expansion	75	0.83	Consolidation	16	0.85	-0.05	Negligible	0.75	0.98	Expansion ∼ Consolidation
SPE	Foundation	8	0.89	Consolidation	14	0.80	0.39	Medium	0.14	0.46	Foundation ∼ Consolidation
	Expansion	71	0.83	Foundation	8	0.89	-0.28	Small	0.20	0.46	Expansion ∼ Foundation
	Expansion	71	0.83	Consolidation	14	0.80	0.15	Small	0.37	0.54	Expansion ∼ Consolidation
ROC AUC	Expansion	62	0.89	Consolidation	9	0.89	0.28	Small	0.18	0.67	Expansion ∼ Consolidation
	Expansion	62	-	Foundation	0	-	-	-	-	-	-
	Foundation	0	-	Consolidation	9	-	-	-	-	-	-
MCC	Expansion	61	0.60	Consolidation	8	0.52	0.19	Small	0.38	0.65	Expansion ∼ Consolidation
	Expansion	61	-	Foundation	2	-	-	-	-	-	-
	Foundation	2	-	Consolidation	8	-	-	-	-	-	-
						Model					
ACC	DL	50	0.83	ML	50	0.84	-0.10	Negligible	0.40	0.56	DL ∼ ML
BA	DL	15	0.83	ML	5	0.83	-0.16	Small	0.63	0.63	DL ∼ ML
SEN	DL	50	0.83	ML	49	0.84	-0.060	Negligible	0.62	0.90	DL ∼ ML
SPE	DL	46	0.82	ML	47	0.83	0.00	Negligible	0.99	0.99	DL ∼ ML
ROC AUC	DL	44	0.89	ML	27	0.89	0.08	Negligible	0.59	0.79	DL ∼ ML
MCC	DL	46	0.61	ML	25	0.57	0.13	Small	0.37	0.65	DL ∼ ML
						Evaluation strategy					
ACC	Random	50	0.85	External	39	0.81	0.34	Medium	0.0059	0.038	Random > External
	Random	50	0.85	Distance	11	0.83	0.21	Small	0.28	0.52	Random ∼ Distance
	Distance	11	0.83	External	39	0.81	0.16	Small	0.43	0.56	Distance ∼ External
BA	Random	10	0.84	External	8	0.78	0.62	Large	0.029	0.058	Random ∼ External
	Random	10	-	Distance	2	-	-	-	-	-	-

Table S5 – continued from previous page

Metric	$Group_A$	N_A	$\tilde{x}_A$	$Group_B$	N_B	$\tilde{x}_B$	r_{bc}	Effect size	p	p_{adj}	Conclusion
	Distance	2	-	External	8	-	-	-	-	-	-
SEN	Random	52	0.84	External	36	0.81	0.11	Small	0.38	0.90	Random ∼ External
	Random	52	0.84	Distance	11	0.81	0.11	Small	0.59	0.90	Random ∼ Distance
	Distance	11	0.81	External	36	0.81	0.020	Negligible	0.93	1.0	Distance ∼ External
SPE	Random	48	0.86	Distance	10	0.73	0.40	Medium	0.048	0.31	Random ∼ Distance
	Random	48	0.86	External	35	0.82	0.23	Small	0.079	0.34	Random ∼ External
	Distance	10	0.73	External	35	0.82	-0.23	Small	0.29	0.53	Distance ∼ External
ROC AUC	Distance	5	0.83	External	29	0.88	-0.21	Small	0.48	0.79	Distance ∼ External
	Random	37	0.89	Distance	5	0.83	0.090	Negligible	0.77	0.85	Random ∼ Distance
	Random	37	0.89	External	29	0.88	0.020	Negligible	0.89	0.89	Random ∼ External
MCC	Distance	10	0.56	External	32	0.60	-0.12	Small	0.59	0.68	Distance ∼ External
	Random	29	0.61	Distance	10	0.56	0.12	Small	0.60	0.68	Random ∼ Distance
	Random	29	0.61	External	32	0.60	0.030	Negligible	0.84	0.84	Random ∼ External
Threshold											
ACC	1	7	0.90	10	75	0.82	0.71	Large	0.0020	0.026	1 > 10
	<10,>30	10	0.90	10	75	0.82	0.44	Medium	0.024	0.092	<10,>30 ∼ 10
	10	75	0.82	<1,>10	8	0.89	-0.47	Medium	0.028	0.092	10 ∼ <1,>10
	1	7	0.90	<1,>10	8	0.89	0.32	Medium	0.32	0.52	1 ∼ <1,>10
	<10,>30	10	0.90	1	7	0.90	-0.17	Small	0.59	0.69	<10,>30 ∼ 1
	<10,>30	10	0.90	<1,>10	8	0.89	0.04	Negligible	0.93	0.93	<10,>30 ∼ <1,>10
BA	<10,>30	0	-	1	0	-	-	-	-	-	-
	<10,>30	0	-	10	19	-	-	-	-	-	-
	<10,>30	0	-	<1,>10	1	-	-	-	-	-	-
	1	0	-	10	19	-	-	-	-	-	-
	1	0	-	<1,>10	1	-	-	-	-	-	-
	10	19	-	<1,>10	1	-	-	-	-	-	-
SEN	<10,>30	10	0.91	10	72	0.83	0.52	Large	0.0078	0.10	<10,>30 ∼ 10
	<10,>30	10	0.91	<1,>10	10	0.74	0.53	Large	0.048	0.31	<10,>30 ∼ <1,>10
	10	72	0.83	<1,>10	10	0.74	0.21	Small	0.29	0.90	10 ∼ <1,>10
	<10,>30	10	0.91	1	7	0.86	0.21	Small	0.49	0.90	<10,>30 ∼ 1
	1	7	0.86	10	72	0.83	0.00	Negligible	0.99	1.0	1 ∼ 10
	1	7	0.86	<1,>10	10	0.74	0.00	Negligible	1.0	1.0	1 ∼ <1,>10
SPE	10	69	0.81	<1,>10	9	0.93	-0.52	Large	0.012	0.16	10 ∼ <1,>10
	<10,>30	9	0.92	<1,>10	9	0.93	-0.36	Medium	0.21	0.46	<10,>30 ∼ <1,>10
	<10,>30	9	0.92	10	69	0.81	0.19	Small	0.37	0.54	<10,>30 ∼ 10
	1	6	0.88	10	69	0.81	0.15	Small	0.56	0.70	1 ∼ 10
	<10,>30	9	0.92	1	6	0.88	-0.19	Small	0.59	0.70	<10,>30 ∼ 1
	1	6	0.88	<1,>10	9	0.93	-0.13	Small	0.72	0.78	1 ∼ <1,>10
ROC AUC	<10,>30	7	0.94	10	55	0.89	0.44	Medium	0.063	0.62	<10,>30 ∼ 10
	<10,>30	7	0.94	1	6	0.89	0.55	Large	0.11	0.62	<10,>30 ∼ 1
	10	55	0.89	<1,>10	3	0.93	-0.28	Small	0.43	0.79	10 ∼ <1,>10
	1	6	0.89	<1,>10	3	0.93	-0.33	Medium	0.55	0.79	1 ∼ <1,>10

Table S5 – continued from previous page

Metric	$Group_A$	N_A	$\tilde{x}_A$	$Group_B$	N_B	$\tilde{x}_B$	r_{bc}	Effect size	p	p_{adj}	Conclusion
	1	6	0.89	10	55	0.89	-0.13	Small	0.60	0.79	1 ∼ 10
	<10,>30	7	0.94	<1,>10	3	0.93	0.24	Small	0.65	0.79	<10,>30 ∼ <1,>10
MCC	<10,>30	3	0.84	10	60	0.60	0.59	Large	0.087	0.65	<10,>30 ∼ 10
	<10,>30	3	0.84	1	6	0.48	0.56	Large	0.24	0.65	<10,>30 ∼ 1
	1	6	0.48	10	60	0.60	-0.21	Small	0.41	0.65	1 ∼ 10
	<10,>30	3	-	<1,>10	2	-	-	-	-	-	-
	1	6	-	<1,>10	2	-	-	-	-	-	-
	10	60	-	<1,>10	2	-	-	-	-	-	-

Table S6. Pairwise Mann-Whitney U comparisons of regression performance metrics across era, model class, and evaluation strategy. For each comparison, group size (N), median value ($\tilde{x}$), rank-biserial correlation (r_{rb}), effect size, p value (p), adjusted p value (p_{adj}), and conclusion are reported. Values were corrected using Benjamini-Hochberg FDR correction on a per-metric basis. Conclusions drawn at $p_{adj} < 0.05$. Dashes indicate comparisons where insufficient group size (N<3) precludes testing. R^2—Coefficient of Determination, RMSE—Root Mean Squared Error, MAE—Mean Absolute Error.

Metric	$Group_A$	N_A	$\tilde{x}_A$	$Group_B$	N_B	$\tilde{x}_B$	r_{rb}	Effect size	p	p_{adj}	Conclusion
						Era					
R2	Expansion	16	0.62	Foundation	5	0.85	-0.65	Large	0.035	0.25	Expansion $\sim$ Foundation
	Consolidation	3	0.68	Foundation	5	0.85	-0.47	Medium	0.37	0.87	Consolidation $\sim$ Foundation
	Expansion	16	0.62	Consolidation	3	0.68	-0.31	Medium	0.43	0.87	Expansion $\sim$ Consolidation
RMSE	Expansion	19	0.60	Foundation	3	0.74	-0.42	Medium	0.27	0.45	Expansion $\sim$ Foundation
	Expansion	19	-	Consolidation	1	-	-	-	-	-	-
	Consolidation	1	-	Foundation	3	-	-	-	-	-	-
MAE	Expansion	10	-	Consolidation	2	-	-	-	-	-	-
	Expansion	10	-	Foundation	2	-	-	-	-	-	-
	Consolidation	2	-	Foundation	2	-	-	-	-	-	-
						Model					
R2	ML	19	0.63	DL	5	0.68	-0.21	Small	0.50	0.87	ML $\sim$ DL
RMSE	ML	19	0.60	DL	4	0.73	-0.17	Small	0.63	0.63	ML $\sim$ DL
MAE	ML	12	-	DL	2	-	-	-	-	-	-
						Evaluation strategy					
R2	Random	16	0.64	External	4	0.65	0.16	Small	0.67	0.94	Random $\sim$ External
	Random	16	0.64	Distance	4	0.59	0.080	Negligible	0.85	0.99	Random $\sim$ Distance
	Distance	4	0.59	External	4	0.65	0.00	Negligible	1.0	1.0	Distance $\sim$ External
RMSE	Random	14	0.59	External	6	0.77	-0.88	Large	0.0025	0.013	Random $<$ External
	Distance	3	0.72	External	6	0.77	-0.78	Large	0.092	0.23	Distance $\sim$ External
	Random	14	0.59	Distance	3	0.72	-0.29	Small	0.49	0.61	Random $\sim$ Distance
MAE	Random	8	0.41	External	3	0.50	-0.42	Medium	0.36	0.60	Random $\sim$ External
	Distance	3	0.45	External	3	0.50	-0.56	Large	0.40	0.60	Distance $\sim$ External
	Random	8	0.41	Distance	3	0.45	-0.25	Small	0.61	0.61	Random $\sim$ Distance

1.5 Data availability for the secondary channels

Table S7. Data availability for the secondary channels in ChEMBL, PubChem, and BindingDB. Presented values correspond to unique compound counts using PubChem CIDs. Counts given in parentheses. %Control denotes assays in which compound activity was measured at a single concentration relative to a reference. Binary denotes assays in which outcomes were encoded as "Active"/"Inactive". Potency denotes assays reporting quantities such as IC_{50}, K_i, or K_d, i.e. values derived from a dose–response curve. For more details on data curation, see Supporting Notes 1–3.

Channel	Organism	Source	%Control	Binary	Potency
K_v7.1	Cavia porcellus	BindingDB (47)	0	0	47
		ChEMBL (47)	0	0	47
		PubChem (47)	0	0	47
		Unique (69)	0	0	69
	Homo sapiens	BindingDB (78)	0	0	78
		ChEMBL (173)	41	0	132
		PubChem (305787)	305653	36	112
		Unique (305854)	305653	36	197
	Mus musculus	PubChem (2)	0	0	2
	Rattus norvegicus	PubChem (7)	7	0	0
	Total unique (305926)		305660	36	263
K_v4.3	Homo sapiens	BindingDB (44)	0	0	44
		ChEMBL (57)	9	0	48
		PubChem (66)	10	21	36
		Unique (85)	10	21	60
	Mus musculus	PubChem (2)	0	0	2
	Rattus norvegicus	PubChem (3)	0	1	2
	Total unique (90)		10	22	64
Kir2.1	Homo sapiens	BindingDB (22)	0	0	22
		ChEMBL (31)	9	0	22
		PubChem (17)	15	2	0
		Unique (33)	24	2	22
	Mus musculus	BindingDB (22)	0	0	22
		ChEMBL (11)	0	0	11
		PubChem (305661)	305611	5	74
		Unique (305666)	305611	5	79
	Total unique (305698)		305634	7	101

1.6 Applicability Domain and Uncertainty Quantification

Table S8. Overview of applicability domain and uncertainty quantification methods used in the surveyed studies.

Study (Year)	Applicability Domain	Uncertainty Quantification
Coi (2009)[34]	Descriptor range	-
Nisius (2009)[38]	Distance to training set	-
Obrezanova (2010)[41]	-	Gaussian process posterior uncertainty
Sinha (2011)[48]	Leverage	-
Kar (2012)[51]	Distance to training set	-
Kirevaa (2013)[57]	Prediction-confidence-based AD	Prediction probability/confidence
Braga (2014)[58]	Distance to training set	-
Gobbi (2016)[66]	Fragment-based defect	-
Alves (2018)[73]	Leverage	-
Munawar (2018)[74]	Distance to training set	-
Siramshetty (2018)[75]	Distance to training set	-
Wacker (2018)[76]	Distance to training set	-
Yang (2018)[77]	Descriptor range	-
Ogura (2019)[84]	Distance to training set	-
Lee (2021)[95]	Distance to training set	-
Delre (2022)[103]	Leverage	-
Kim (2022)[106]	-	Monte Carlo dropout
Krishna (2022)[108]	Distance to training set	-
Banerjee (2023)[114]	Response-discordance AD	-
Chen (2023)[115]	Distance to training set	-
Wang (2023)[119]	Leverage	-
Vittorio (2023)[120]	Consensus AD	Ensemble variance
Arab (2024)[122]	-	Ensemble prediction confidence
Liu (2024)[125]	Descriptor range; distance to training set	-
Sanches (2024)[126]	Leverage	-
Cano (2025)[129]	Distance to training set	-
Gambacorta (2025)[131]	Prediction-score reliability AD	-
Yu (2025)[144]	Distance to training set	-
Zhang (2025)[145]	Similarity-activity landscape	-
Enokiya (2026)[149]	Distance to training set	Monte Carlo dropout

Table S8 – continued from previous page

Study (Year)	Applicability Domain	Uncertainty Quantification
Jovanović (2026)[150]	Distance to training set	-
Zhang (2026)[154]	Similarity-activity landscape	-

1.7 Availability

Table S9. Summary of availability of data, code, model, and prediction platforms.

Study (Year)	Data	Code	Models	Platform	In-paper	Ref
Ponti (2001)[156]	Yes	No	No	No	Data	No
Cavalli (2002)[1]	Yes	No	Yes	No	Data, Pharmacophore	No
Ekins (2002)[2]	Yes	No	Yes	No	Data, Pharmacophore	No
Roche (2002)[3]	Partially	No	No	No	Data†	No
Ekins (2003)[4]	No	No	No	No	No	No
Keserü (2003)[5]	Yes	No	Yes	No	Data, Equation	No
Pearlstein (2003)[6]	Yes	No	Partially	No	Data, Interactions	No
Aronov (2004)[8]	No	No	Yes	No	Pharmacophore	No
Aptula (2004)[7]	Yes	No	Yes	No	Data, Equation	No
Bains (2004)[9]	Yes	No	Yes	No	Data, Descriptors, Pharmacophore	No
Fioravanzo (2004)[10]	Yes (SI)	No	No	No	Data	No
Cianchetta (2005)[11]	Yes (SI)	No	Partially	No	Data†, Pharmacophore	No
O'Brien (2005)[12]	No	No	No	No	No	No
Tobita (2005)[13]	Yes	No	No	No	Data, Descriptors	No
Aronov (2006)[14]	No	No	Yes	No	Pharmacophore	No
Coi (2006)[15]	Yes	No	Yes	No	Data, Equation	No
Dubus (2006)[16]	No	No	Yes	No	Descriptors, Decision tree	No
Ekins (2006)[17]	Yes	No	No	No	Data, Descriptors	No
Gepp (2006)[18]	No	No	Yes	No	Decision tree, Descriptors	No
Seierstad (2006)[19]	Partially	No	Partially	No	Data†, Descriptors	No
Sun (2006)[20]	Partially	No	Partially	No	Data†, Descriptors	No
Song (2006)[21]	Partially	No	Partially	No	Data†, Fragments	No
Yoshida (2006)[22]	Yes	No	Yes	No	Data, Equation	No
Du (2007)[23]	Yes	No	Yes	No	Data, Equation	No
Leong (2007)[24]	Yes	No	Partially	No	Data, Pharmacophore	No
Obrezanova (2007)[25]	Yes (SI)	No	No	No	No	No
Chekmarev (2008)[26]	Yes (SI)	No	No	No	No	No
Gunturi (2008)[27]	Yes	No	Partially	No	Data, Descriptors	No
Jia (2008)[28]	Partially	No	No	No	Data†	No
Kramer (2008)[29]	Yes (SI)	No	Partially	No	Pharmacophore	No

Table S9 – continued from previous page

Study (Year)	Data	Code	Models	Platform	In-paper	Ref
Li (2008)[30]	Yes (SI)	No	No	No	No	No
Thai◇(2008)[31]	Yes (SI)	No	No	No	Descriptors	No
Thai◇(2008)[32]	Yes (SI)	No	No	No	Descriptors	No
Wang (2008)[33]	No	No	No	No	Descriptors	No
Coi (2009)[34]	Yes	No	Yes	No	Data, Equation	No
Ermondi (2009)[35]	Yes	No	No	No	Data	No
Hansen (2009)[36]	No	No	No	No	No	No
Nisius◇(2009)[37]	Yes (SI)	No	Partially	No	No	No
Nisius◇(2009)[38]	Yes (SI)	No	No	No	No	No
Thai (2009)[39]	Yes (SI)	No	No	No	No	No
Doddareddy (2010)[40]	Yes (SI)	No	Partially	No	Fragments, Decision tree	No
Obrezanova (2010)[41]	Yes (SI)	No	No	No	No	No
Su (2010)[42]	Yes (SI)	No	Partially	No	Equation	No
Wiśniowska (2010)[43]	No	No	No	No	Descriptors	No
Cuny (2011)[44]	No	No	Partially	No	Descriptors, Pharmacophore, Equation	No
Kim (2011)[45]	No	No	No	No	Fragments	No
Obiol-Pardo (2011)[46]	Yes (SI)	No	Partially	No	No	No
Robinson (2011)[47]	Yes (SI)	No	No	No	Descriptors	No
Sinha (2011)[48]	Yes	No	No	No	Data	No
Shen (2011)[49]	No	No	Partially	No	Descriptors	No
Broccatelli (2012)[50]	Yes (SI)	No	Partially	No	No	No
Kar (2012)[51]	Yes (SI)	No	Yes	No	Equation	No
Polak (2012)[52]	Yes	No	No	No	Data, Hyperparameters	No
Tan (2012)[53]	Yes	No	Partially	No	Data, Equation	No
Wang (2012)[54]	Yes	No	Yes	No	Decision tree, Fragments	Data¶
Wiśniowska (2012)[55]	Yes	No	No	No	Data, Hyperparameters	No
Czodrowski (2013)[56]	Yes (SI)	Yes	No	No	Fragments	Code
Kireeva (2013)[57]	No	No	No	No	No	No
Braga (2014)[58]	Yes	No	No	Yes	No	Data¶; Platform¶
Kratz (2014)[59]	Partially	No	Yes	No	Data, Pharmacophore	No
Liu (2014)[60]	No	No	No	No	Fragments	No
Braga (2015)[61]	Yes (Online)	No	No	Yes	No	Platform¶

Table S9 – continued from previous page

Study (Year)	Data	Code	Models	Platform	In-paper	Ref
Du (2015)[62]	Yes (SI)	No	No	No	No	No
Ma (2015)[63]	No	No	Partially	No	Hyperparameters	No
Chavan (2016)[64]	Yes (SI)	No	No	No	No	No
Didziapetris (2016)[65]	Yes (SI)	No	No	No	No	No
Gobbi (2016)[66]	Yes	No	Partially	No	Equation	No
Sheridan (2016)[67]	No	No	No	No	No	No
Wang (2016)[68]	Yes (SI)	No	Yes	No	Decision tree, Pharmacophore	No
Zhang (2016)[69]	Yes (SI)	No	No	No	Fragments	No
Chemi (2017)[70]	Yes (SI)	No	No	No	Pharmacophore	No
Li (2017)[71]	Yes	No	No	Yes	No	Data, Model
Sun (2017)[72]	Yes (SI)	No	No	No	Pharmacophore	No
Alves (2018)[73]	Yes	No	No	Yes	No	Code*
Munawar (2018)[74]	Yes (SI)	No	No	No	Interactions, Pharmacophore	No
Siramshetty (2018)[75]	Yes	Yes	No	No	No	Data, Code
Wacker (2018)[76]	No	No	No	No	No	No
Yang (2018)[77]	No	No	No	Yes	No	Platform
Cai (2019)[78]	Yes (SI)	Yes	No	No	No	Code
Hu (2019)[79]	No	No	No	No	No	No
Konda (2019)[80]	Yes (SI)	No	No	No	No	No
Lee (2019)[81]	On Request	No	No	Yes	Features	Platform
Munawar (2019)[82]	No	No	No	No	Interactions	No
Negami (2019)[83]	Yes (SI)	No	No	No	No	No
Ogura (2019)[84]	Yes	No	No	Yes	No	Platform
Zhang (2019)[85]	Yes (SI)	No	No	No	No	No
Choi (2020)[86]	Yes (SI)	No	No	No	No	No
Khalifa (2020)[87]	Yes (SI)	No	No	No	No	No
Kim (2020)[88]	No	No	No	No	No	No
Liu (2020)[89]	No	No	No	No	Fragments	No
Ryu (2020)[90]	Partially	No	Yes	No	No	Platform¶
Siramshetty (2020)[91]	Yes	Yes	Yes	No	No	Data, Code, Model
Wang (2020)[92]	On Request	No	No	No	Algorithm, Parameters	No
Creanza (2021)[93]	Yes (SI)	No	No	No	Interactions, Protein	No

Table S9 – continued from previous page

Study (Year)	Data	Code	Models	Platform	In-paper	Ref
Karim (2021)[94]	Yes	Yes	No	No	No	Data, Code
Lee (2021)[95]	Yes	Yes	No	No	No	Data, Code
Meng (2021)[96]	Yes (SI)	No	No	No	No	No
Moorthy (2021)[97]	Yes (SI)	No	Yes	No	Fragments, Equation	No
Sato (2021)[98]	No	No	No	No	No	Platform¶
Stergiopoulos (2021)[99]	Yes	No	Yes	No	Data, Experimental, Equation	No
Wu (2021)[100]	Yes	Yes	Yes	No	No	Data, Code, Model
Xiong (2021)[101]	No	No	No	Yes	No	Platform
Arab (2022)[102]	Yes	Yes	Yes	No	No	Data, Code, Model
Delre (2022)[103]	Yes	Yes	Yes	No	No	Data, Code, Workflow
Ding (2022)[104]	Yes (SI)	Yes	Yes	No	No	Data, Code, Model
Ishihara (2022)[105]	No	No	No	No	No	No
Kim (2022)[106]	Yes	Yes	Yes	No	No	Data, Code, Model
Kong (2022)[107]	No	No	No	No	No	No
Krishna (2022)[108]	Yes (SI)	Yes	No	No	No	Code
Lanevskij (2022)[109]	Yes (SI)	No	No	No	No	No
Melnikov (2022)[110]	No	No	No	No	No	No
Shan (2022)[111]	Yes	Yes	No	No	No	Data, Code
Zhang (2022)[112]	Yes (Online)	No	No	Yes	Fragments	Platform¶
Arab (2023)[113]	Yes	Yes	Yes	No	No	Data, Code, Model
Banerjee (2023)[114]	Yes (SI)	No	Yes	No	No	Model
Chen (2023)[115]	Yes (SI)	No	No	No	Fragments	No
Das (2023)[116]	Yes (SI)	No	Yes	No	Equation	No
Feng (2023)[117]	Yes	Yes	No	No	No	Data, Code
Wang◇(2023)[118]	Yes	Yes	No	No	No	Data, Code
Wang◇(2023)[119]	Yes	Yes	No	No	No	Data, Code
Vittorio (2023)[120]	Yes	No	Yes	No	No	Data, Model
Ylipää (2023)[121]	No	No	No	No	No	No
Arab (2024)[122]	Yes	Yes	Yes	No	No	Data, Code, Model
Chen (2024)[123]	No	No	No	Yes	Fragments	Platform
He (2024)[124]	Partially	Yes	Yes	No	No	Data, Code; Model
Liu (2024)[125]	No	No	No	No	No	No

Table S9 – continued from previous page

Study (Year)	Data	Code	Models	Platform	In-paper	Ref
Sanches (2024)[126]	Yes (SI)	Yes	No	Yes	No	Data, Code; Platform
Wang (2024)[127]	Yes	Yes	No	Yes	No	Data, Code; Platform
Yang (2024)[128]	Yes	Yes	Yes	No	No	Data, Code, Model
Cano (2025)[129]	No	No	No	Yes	No	Platform
Feng (2025)[130]	Yes	Yes	Yes	No	No	Data, Code; Model
Gambacorta (2025)[131]	Yes	Yes	No	Yes	No	Data, Code; Platform
Han (2025)[132]	Yes	Yes	Yes	No	No	Data, Code, Model
Hossain (2025)[133]	Yes	Yes	Yes	No	No	Data, Code, Model
Jin (2025)[134]	No	Yes	No	No	No	Code
Jing (2025)[135]	No	No	No	No	No	No
Kyro (2025)[136]	Yes	Yes	Yes	No	No	Data, Code, Model
Lee (2025)[137]	Yes	Yes	No	No	No	Data, Code
Liu (2025)[139]	No	No	No	No	No	No
Liu (2025)[138]	No	Yes	No	No	No	Code
Mohammad (2025)[140]	Yes	Yes	No	No	No	Data, Code
Tran-Nguyen (2025)[141]	Yes	Yes	No	No	No	Data, Code
Xu (2025)[142]	Yes (SI)	No	No	No	No	No
Yang (2025)[143]	No	Yes	No	No	No	Code
Yu (2025)[144]	Yes	No	No	Yes	No	Data,Platform
Zhang (2025)[145]	Yes	Yes	Yes	No	No	Data, Code, Model
Agarwal (2026)[146]	Yes	Yes	Yes	No	No	Data, Code, Model
Beidja (2026)[147]	Yes	Yes	No	No	No	Data, Code
Chu (2026)[148]	Yes	Yes	No	No	No	Data, Code
Enokiya (2026)[149]	No	No	No	No	No	Data, Code‡
Jovanović (2026)[150]	Yes	Yes	Yes	Yes	No	Data, Code, Model; Platform
Liang (2026)[151]	No	No	No	No	No	Platform¶
Su (2026)[152]	No (request)	No	No	No	No	No
Sun (2026)[153]	Yes (SI)	No	No	No	No	No
Zhang (2026)[154]	Yes (SI)	Yes	Yes	No	No	Data, Code, Model
Zhao (2026)[155]	Yes	Yes	No	No	No	Data, Code

Annotations: †not all values available, ¶original link deprecated/not working, *unable to verify, ◇separate studies, ‡availability claimed in paper but link not provided

2 Notes

Note S1: ChEMBL data extraction and curation. Bioactivity data were retrieved from ChEMBL[159] (version 36) as part of a broader curation workflow covering voltage-gated potassium, sodium, calcium, inward-rectifier, and HCN ion channels; the present review uses the subset corresponding to the six CiPA-relevant cardiac ion channels. At the assay level, target annotations, organisms, expression systems, and assay descriptions were reviewed and supplemented using information extracted from assay descriptions, titles, and abstracts. Assays were flagged and manually checked for issues such as mutant proteins, ambiguous target assignments (e.g., protein families), unsuitable expression systems, low confidence scores, and non-relevant endpoints (e.g., channel activation or opening rather than block). Assays were additionally classified along two axes—mechanism and readout—to distinguish direct electrophysiological measurements from proxy assays such as ion-flux, membrane-potential, intracellular ion, and ligand-binding assays.

At the measurement level, entries with invalid or unusable activity comments were removed, binary outcomes were converted to censored values where possible, and endpoint labels were standardised. Ambiguous endpoint types were resolved to IC_{50}, K_i, K_d, or single-concentration inhibition where inferrable from assay descriptions and associated metadata; non-convertible endpoint types were excluded. Quantitative values were harmonised to common units and converted to a common logarithmic scale for potency data. Extreme outliers were first removed using broad quantile-based bounds, after which values outside fixed ranges (4–11 pX units for IC_{50}, K_i, K_d, and −20% to 100% for inhibition) were censored or excluded as appropriate.

At the compound level, chemical structures were retrieved from ChEMBL using *molregno* and merged with the filtered measurements; entries lacking structural information were excluded. Gene combinations were then mapped to canonical channel labels, and the CiPA-relevant subset was extracted for the present analysis. Compound counts reflect unique ChEMBL molecule IDs per channel and should be interpreted as upper bounds, as downstream study-specific curation, structure standardisation, and deduplication will further reduce the number of compounds available for model training.

Note S2. PubChem data extraction and curation. For K_v*7.1*, K_v*4.3*, and *Kir2.1*, PubChem BioAssay records[161] were retrieved by searching PubChem with the channel name and downloading all returned BioAssay entries. Assay descriptions were manually reviewed, and only assays reporting inhibition or block of the respective channel were retained; assays reporting unrelated outcomes, such as opening, activation, or agonist activity, were excluded. For the retained assays, gene identity, channel name, and target organism were manually annotated from the assay descriptions and other available fields. Record-level measurements were then downloaded for all retrieved assays, joined with the filtered assay list, and curated at the measurement level. Retained records were further separated into broad assay categories according to the reported endpoint type ("%control", "Binary", "Potency").

Note S3. BindingDB data extraction and curation. For K_v*7.1*, K_v*4.3*, and *Kir2.1*, relevant target proteins and genes were first identified in UniProt[174], and the corresponding linked entries in BindingDB[163] were then collected. As assay descriptions were not directly available in BindingDB, the associated patents or publications linked to each entry were checked to confirm relevance to the respective channel. As previously, only entries corresponding to inhibition or block were retained for further analysis. Gene identity, channel name, and target organism were annotated from the retrieved records. As entries from BindignDB contain only measurements like IC_{50}, K_i, and K_d, all were assigned to the "Potency" category.

3 References

[1] A. Cavalli, E. Poluzzi, F. De Ponti, and M. Recanatini, “Toward a pharmacophore for drugs inducing the long qt syndrome: Insights from a comfa study of herg k+ channel blockers,” *Journal of Medicinal Chemistry*, vol. 45, p. 3844–3853, July 2002.

[2] S. Ekins, W. J. Crumb, R. D. Sarazan, J. H. Wikel, and S. A. Wrighton, “Three-dimensional quantitative structure-activity relationship for inhibition of human ether-a-go-go-related gene potassium channel,” *The Journal of Pharmacology and Experimental Therapeutics*, vol. 301, p. 427–434, May 2002.

[3] O. Roche, G. Trube, J. Zuegge, P. Pflimlin, A. Alanine, and G. Schneider, “A virtual screening method for prediction of the herg potassium channel liability of compound libraries,” *ChemBioChem*, vol. 3, p. 455, May 2002.

[4] S. Ekins, “In silico approaches to predicting drug metabolism, toxicology and beyond,” *Biochemical Society Transactions*, vol. 31, p. 611–614, June 2003.

[5] G. M. Keserü, “Prediction of herg potassium channel affinity by traditional and hologram qsar methods,” *Bioorganic & Medicinal Chemistry Letters*, vol. 13, p. 2773–2775, Aug. 2003.

[6] R. A. Pearlstein, R. J. Vaz, J. Kang, X.-L. Chen, M. Preobrazhenskaya, A. E. Shchekotikhin, A. M. Korolev, L. N. Lysenkova, O. V. Miroshnikova, J. Hendrix, and D. Rampe, “Characterization of herg potassium channel inhibition using comsia 3d qsar and homology modeling approaches,” *Bioorganic & Medicinal Chemistry Letters*, vol. 13, p. 1829–1835, May 2003.

[7] A. Aptula and M. Cronin, “Prediction of herg k+ blocking potency: Application of structural knowledge,” *SAR and QSAR in Environmental Research*, vol. 15, p. 399–411, Oct. 2004.

[8] A. M. Aronov and B. B. Goldman, “A model for identifying herg k+ channel blockers,” *Bioorganic & Medicinal Chemistry*, vol. 12, p. 2307–2315, May 2004.

[9] W. Bains, A. Basman, and C. White, "Herg binding specificity and binding site structure: evidence from a fragment-based evolutionary computing sar study," *Progress in Biophysics and Molecular Biology*, vol. 86, p. 205–233, Oct. 2004.

[10] E. Fioravanzo, N. Cazzolla, L. Durando, C. Ferrari, M. Mabilia, R. Ombrato, and M. D. Parenti, "General and independent approaches to predict herg affinity values," *Internet Electronic Journal of Molecular Design*, vol. 2, 2004.

[11] G. Cianchetta, Y. Li, J. Kang, D. Rampe, A. Fravolini, G. Cruciani, and R. J. Vaz, "Predictive models for herg potassium channel blockers," *Bioorganic & Medicinal Chemistry Letters*, vol. 15, p. 3637–3642, Aug. 2005.

[12] S. E. O'Brien and M. J. de Groot, "Greater than the sum of its parts: Combining models for useful admet prediction," *Journal of Medicinal Chemistry*, vol. 48, p. 1287–1291, Feb. 2005.

[13] M. Tobita, T. Nishikawa, and R. Nagashima, "A discriminant model constructed by the support vector machine method for herg potassium channel inhibitors," *Bioorganic & Medicinal Chemistry Letters*, vol. 15, p. 2886–2890, June 2005.

[14] A. M. Aronov, "Common pharmacophores for uncharged human ether-a-go-go-related gene (herg) blockers," *Journal of Medicinal Chemistry*, vol. 49, p. 6917–6921, Oct. 2006.

[15] A. Coi, I. Massarelli, L. Murgia, M. Saraceno, V. Calderone, and A. M. Bianucci, "Prediction of herg potassium channel affinity by the codessa approach," *Bioorganic & Medicinal Chemistry*, vol. 14, p. 3153–3159, May 2006.

[16] E. Dubus, I. Ijjaali, F. Petitet, and A. Michel, "In silico classification of herg channel blockers: a knowledge-based strategy," *ChemMedChem*, vol. 1, p. 622–630, May 2006.

[17] S. Ekins, K. V. Balakin, N. Savchuk, and Y. Ivanenkov, "Insights for human ether-a-go-go-related gene potassium channel inhibition using recursive partitioning and kohonen and sammon mapping techniques," *Journal of Medicinal Chemistry*, vol. 49, p. 5059–5071, Aug. 2006.

[18] M. M. Gepp and M. C. Hutter, "Determination of herg channel blockers using a decision tree," *Bioorganic & Medicinal Chemistry*, vol. 14, p. 5325–5332, Aug. 2006.

[19] M. Seierstad and D. K. Agrafiotis, "A qsar model of herg binding using a large, diverse, and internally consistent training set," *Chemical Biology & Drug Design*, vol. 67, p. 284–296, Apr. 2006.

[20] H. Sun, "An accurate and interpretable bayesian classification model for prediction of herg liability," *ChemMedChem*, vol. 1, p. 315–322, Mar. 2006.

[21] M. Song and M. Clark, "Development and evaluation of an in silico model for herg binding," *Journal of Chemical Information and Modeling*, vol. 46, p. 392–400, Dec. 2006.

[22] K. Yoshida and T. Niwa, "Quantitative structure-activity relationship studies on inhibition of herg potassium channels," *Journal of Chemical Information and Modeling*, vol. 46, p. 1371–1378, Mar. 2006.

[23] L. Du, M. Li, Q. You, and L. Xia, "A novel structure-based virtual screening model for the herg channel blockers," *Biochemical and Biophysical Research Communications*, vol. 355, p. 889–894, Apr. 2007.

[24] M. K. Leong, "A novel approach using pharmacophore ensemble/support vector machine (phe/svm) for prediction of herg liability," *Chemical Research in Toxicology*, vol. 20, p. 217–226, Jan. 2007.

[25] O. Obrezanova, G. Csányi, J. M. R. Gola, and M. D. Segall, "Gaussian processes: A method for automatic qsar modeling of adme properties," *Journal of Chemical Information and Modeling*, vol. 47, p. 1847–1857, June 2007.

[26] D. S. Chekmarev, V. Kholodovych, K. V. Balakin, Y. Ivanenkov, S. Ekins, and W. J. Welsh, "Shape signatures: New descriptors for predicting cardiotoxicity in silico," *Chemical Research in Toxicology*, vol. 21, p. 1304–1314, May 2008.

[27] S. Gunturi, K. Archana, A. Khandelwal, and R. Narayanan, "Prediction of herg potassium channel blockade using knn-qsar and local lazy regression methods," *QSAR & Combinatorial Science*, vol. 27, p. 1305–1317, Dec. 2008.

[28] L. Jia and H. Sun, "Support vector machines classification of herg liabilities based on atom types," *Bioorganic & Medicinal Chemistry*, vol. 16, p. 6252–6260, June 2008.

[29] C. Kramer, B. Beck, J. Kriegl, and T. Clark, "A composite model for herg blockade," *ChemMedChem*, vol. 3, p. 254–265, Feb. 2008.

[30] Q. Li, F. S. Jørgensen, T. Oprea, S. Brunak, and O. Taboureau, "herg classification model based on a combination of support vector machine method and grind descriptors," *Molecular Pharmaceutics*, vol. 5, p. 117–127, Jan. 2008.

[31] K.-M. Thai and G. F. Ecker, "A binary qsar model for classification of herg potassium channel blockers," *Bioorganic and Medicinal Chemistry*, vol. 16, p. 4107–4119, Apr. 2008.

[32] K. Thai and G. F. Ecker, "Classification models for herg inhibitors by counter-propagation neural networks," *Chemical Biology & Drug Design*, vol. 72, p. 279–289, Sept. 2008.

[33] M. Wang, X. Yang, and Y. Xue, "Identifying herg potassium channel inhibitors by machine learning methods," *QSAR & Combinatorial Science*, vol. 27, p. 1028–1035, Aug. 2008.

[34] A. Coi, I. Massarelli, M. Saraceno, N. Carli, L. Testai, V. Calderone, and A. M. Bianucci, "Quantitative structure–activity relationship models for predicting biological properties, developed by combining structure- and ligand-based approaches: An application to the human ether-a-go-go-related gene potassium channel inhibition," *Chemical Biology & Drug Design*, vol. 74, p. 416–433, Sept. 2009.

[35] G. Ermondi, S. Visentin, and G. Caron, "Grind-based 3d-qsar and comfa to investigate topics dominated by hydrophobic interactions: The case of herg k+ channel blockers," *European Journal of Medicinal Chemistry*, vol. 44, p. 1926–1932, May 2009.

[36] K. Hansen, F. Rathke, T. Schroeter, G. Rast, T. Fox, J. M. Kriegl, and S. Mika, "Bias-correction of regression models: A case study on herg inhibition," *Journal of Chemical Information and Modeling*, vol. 49, p. 1486–1496, May 2009.

[37] B. Nisius, A. H. Göller, and J. Bajorath, "Combining cluster analysis, feature selection and multiple support vector machine models for the identification of human ether-a-go-go related gene channel blocking compounds," *Chemical Biology & Drug Design*, vol. 73, p. 17–25, Jan. 2009.

[38] B. Nisius and A. H. Göller, "Similarity-based classifier using topomers to provide a knowledge base for herg channel inhibition," *Journal of Chemical Information and Modeling*, vol. 49, p. 247–256, Jan. 2009.

[39] K.-M. Thai and G. F. Ecker, "Similarity-based sibar descriptors for classification of chemically diverse herg blockers," *Molecular Diversity*, vol. 13, p. 321–336, Feb. 2009.

[40] M. Doddareddy, E. Klaasse, Shagufta, A. IJzerman, and A. Bender, "Prospective validation of a comprehensive in silico herg model and its applications to commercial compound and drug databases," *ChemMedChem*, vol. 5, p. 716–729, Apr. 2010.

[41] O. Obrezanova and M. D. Segall, "Gaussian processes for classification: Qsar modeling of admet and target activity," *Journal of Chemical Information and Modeling*, vol. 50, p. 1053–1061, Apr. 2010.

[42] B.-H. Su, M.-y. Shen, E. X. Esposito, A. J. Hopfinger, and Y. J. Tseng, "In silico binary classification qsar models based on 4d-fingerprints and moe descriptors for prediction of herg blockage," *Journal of Chemical Information and Modeling*, vol. 50, p. 1304–1318, June 2010.

[43] B. Wiśniowska *et al.*, "Randomforest based assessment of the herg channel inhibition potential for the early drug cardiotoxicity testing," *Bio-Algorithms and Med-Systems*, vol. 6, no. 12-Suplement, pp. 211–212, 2010.

[44] L. Du-Cuny, L. Chen, and S. Zhang, "A critical assessment of combined ligand- and structure-based approaches to herg channel blocker modeling," *Journal of Chemical Information and Modeling*, vol. 51, p. 2948–2960, Oct. 2011.

[45] J.-H. Kim, C.-H. Chae, S.-M. Kang, J.-Y. Lee, G.-N. Lee, S.-H. Hwang, and N.-S. Kang, "The predictive qsar model for herg inhibitors using bayesian and random forest classification method," *Bulletin of the Korean Chemical Society*, vol. 32, no. 4, pp. 1237–1240, 2011.

[46] C. Obiol-Pardo, J. Gomis-Tena, F. Sanz, J. Saiz, and M. Pastor, "A multiscale simulation system for the prediction of drug-induced cardiotoxicity," *Journal of Chemical Information and Modeling*, vol. 51, p. 483–492, Jan. 2011.

[47] R. L. Marchese Robinson, R. C. Glen, and J. B. O. Mitchell, "Development and comparison of herg blocker classifiers: Assessment on different datasets yields markedly different results," *Molecular Informatics*, vol. 30, p. 443–458, May 2011.

[48] N. Sinha and S. Sen, "Predicting herg activities of compounds from their 3d structures: Development and evaluation of a global descriptors based qsar model," *European Journal of Medicinal Chemistry*, vol. 46, p. 618–630, Feb. 2011.

[49] M.-Y. Shen, B.-H. Su, E. X. Esposito, A. J. Hopfinger, and Y. J. Tseng, "A comprehensive support vector machine binary herg classification model based on extensive but biased end point herg data sets," *Chemical Research in Toxicology*, vol. 24, p. 934–949, May 2011.

[50] F. Broccatelli, R. Mannhold, A. Moriconi, S. Giuli, and E. Carosati, "Qsar modeling and data mining link torsades de pointes risk to the interplay of extent of metabolism, active transport, and herg liability," *Molecular Pharmaceutics*, vol. 9, p. 2290–2301, July 2012.

[51] S. Kar and K. Roy, "Prediction of herg potassium channel blocking actions using combination of classification and regression based models: A mixed descriptors approach," *Molecular Informatics*, vol. 31, p. 879–894, Nov. 2012.

[52] S. Polak, B. Wiśniowska, A. Glinka, K. Fijorek, and A. Mendyk, "Slow delayed rectifying potassium current (iks) – analysis of the in vitro inhibition data and predictive model development," *Journal of Applied Toxicology*, vol. 33, p. 723–739, Feb. 2012.

[53] Y. Tan, Y. Chen, Q. You, H. Sun, and M. Li, "Predicting the potency of herg k+ channel inhibition by combining 3d-qsar pharmacophore and 2d-qsar models," *Journal of Molecular Modeling*, vol. 18, p. 1023–1036, June 2012.

[54] S. Wang, Y. Li, J. Wang, L. Chen, L. Zhang, H. Yu, and T. Hou, "Admet evaluation in drug discovery. 12. development of binary classification models for prediction of herg potassium channel blockage," *Molecular Pharmaceutics*, vol. 9, p. 996–1010, Mar. 2012.

[55] B. Wiśniowska, A. Mendyk, K. Fijorek, A. Glinka, and S. Polak, "Predictive model for l-type channel inhibition: multichannel block in qt prolongation risk assessment," *Journal of Applied Toxicology*, vol. 32, p. 858–866, July 2012.

[56] P. Czodrowski, "herg me out," *Journal of Chemical Information and Modeling*, vol. 53, p. 2240–2251, Aug. 2013.

[57] N. Kireeva, S. Kuznetsov, A. Bykov, and A. Yu. Tsivadze, "Towardsin silicoidentification of the human ether-a-go-go-related gene channel blockers: discriminative vs. generative classification models," *SAR and QSAR in Environmental Research*, vol. 24, p. 103–117, Feb. 2013.

[58] R. Braga, V. Alves, M. Silva, E. Muratov, D. Fourches, A. Tropsha, and C. Andrade, "Tuning herg out: Antitarget qsar models for drug development," *Current Topics in Medicinal Chemistry*, vol. 14, p. 1399–1415, June 2014.

[59] J. M. Kratz, D. Schuster, M. Edtbauer, P. Saxena, C. E. Mair, J. Kirchebner, B. Matuszczak, I. Baburin, S. Hering, and J. M. Rollinger, "Experimentally validated herg pharmacophore models as cardiotoxicity prediction tools," *Journal of Chemical Information and Modeling*, vol. 54, p. 2887–2901, Sept. 2014.

[60] L.-l. Liu, J. Lu, Y. Lu, M.-y. Zheng, X.-m. Luo, W.-l. Zhu, H.-l. Jiang, and K.-x. Chen, "Novel bayesian classification models for predicting compounds blocking herg potassium channels," *Acta Pharmacologica Sinica*, vol. 35, p. 1093–1102, June 2014.

[61] R. C. Braga, V. M. Alves, M. F. B. Silva, E. Muratov, D. Fourches, L. M. Lião, A. Tropsha, and C. H. Andrade, "Pred-herg: A novel web-accessible computational tool for predicting cardiac toxicity," *Molecular Informatics*, vol. 34, p. 698–701, July 2015.

[62] F. Du, J. J. Babcock, H. Yu, B. Zou, and M. Li, "Global analysis reveals families of chemical motifs enriched for herg inhibitors," *PLOS ONE*, vol. 10, p. e0118324, Feb. 2015.

[63] J. Ma, R. P. Sheridan, A. Liaw, G. E. Dahl, and V. Svetnik, "Deep neural nets as a method for quantitative structure-activity relationships," *Journal of Chemical Information and Modeling*, vol. 55, p. 263–274, Feb. 2015.

[64] S. Chavan, A. Abdelaziz, J. G. Wiklander, and I. A. Nicholls, "A k-nearest neighbor classification of herg k+ channel blockers," *Journal of Computer-Aided Molecular Design*, vol. 30, p. 229–236, Feb. 2016.

[65] R. Didziapetris and K. Lanevskij, "Compilation and physicochemical classification analysis of a diverse herg inhibition database," *Journal of Computer-Aided Molecular Design*, vol. 30, p. 1175–1188, Oct. 2016.

[66] M. Gobbi, M. Beeg, M. A. Toropova, A. A. Toropov, and M. Salmona, "Monte carlo method for predicting of cardiac toxicity: herg blocker compounds," *Toxicology Letters*, vol. 250–251, p. 42–46, May 2016.

[67] R. P. Sheridan, W. M. Wang, A. Liaw, J. Ma, and E. M. Gifford, "Extreme gradient boosting as a method for quantitative structure-activity relationships," *Journal of Chemical Information and Modeling*, vol. 56, p. 2353–2360, Dec. 2016.

[68] S. Wang, H. Sun, H. Liu, D. Li, Y. Li, and T. Hou, "Admet evaluation in drug discovery. 16. predicting herg blockers by combining multiple pharmacophores and machine learning approaches," *Molecular Pharmaceutics*, vol. 13, p. 2855–2866, July 2016.

[69] C. Zhang, Y. Zhou, S. Gu, Z. Wu, W. Wu, C. Liu, K. Wang, G. Liu, W. Li, P. W. Lee, and Y. Tang, "In silico prediction of herg potassium channel blockage by chemical category approaches," *Toxicology Research*, vol. 5, no. 2, p. 570–582, 2016.

[70] G. Chemi, S. Gemma, G. Campiani, S. Brogi, S. Butini, and M. Brindisi, "Computational tool for fast in silico evaluation of herg k+ channel affinity," *Frontiers in Chemistry*, vol. 5, Feb. 2017.

[71] X. Li, Y. Zhang, H. Li, and Y. Zhao, "Modeling of the herg k+ channel blockage using online chemical database and modeling environment (ochem)," *Molecular Informatics*, vol. 36, Aug. 2017.

[72] H. Sun, R. Huang, M. Xia, S. Shahane, N. Southall, and Y. Wang, "Prediction of herg liability – using svm classification, bootstrapping and jackknifing," *Molecular Informatics*, vol. 36, Dec. 2017.

[73] V. M. Alves, A. Golbraikh, S. J. Capuzzi, K. Liu, W. I. Lam, D. R. Korn, D. Pozefsky, C. H. Andrade, E. N. Muratov, and A. Tropsha, "Multi-descriptor read across (mudra): A simple and transparent approach for developing accurate quantitative structure–activity relationship models," *Journal of Chemical Information and Modeling*, vol. 58, p. 1214–1223, May 2018.

[74] S. Munawar, M. J. Windley, E. G. Tse, M. H. Todd, A. P. Hill, J. I. Vandenberg, and I. Jabeen, "Experimentally validated pharmacoinformatics approach to predict herg inhibition potential of new chemical entities," *Frontiers in Pharmacology*, vol. 9, Sept. 2018.

[75] V. B. Siramshetty, Q. Chen, P. Devarakonda, and R. Preissner, "The catch-22 of predicting herg blockade using publicly accessible bioactivity data," *Journal of Chemical Information and Modeling*, vol. 58, p. 1224–1233, May 2018.

[76] S. Wacker and S. Y. Noskov, "Performance of machine learning algorithms for qualitative and quantitative prediction drug blockade of herg1 channel," *Computational Toxicology*, vol. 6, p. 55–63, May 2018.

[77] H. Yang, C. Lou, L. Sun, J. Li, Y. Cai, Z. Wang, W. Li, G. Liu, and Y. Tang, "admetsar 2.0: web-service for prediction and optimization of chemical admet properties," *Bioinformatics*, vol. 35, p. 1067–1069, Aug. 2018.

[78] C. Cai, P. Guo, Y. Zhou, J. Zhou, Q. Wang, F. Zhang, J. Fang, and F. Cheng, "Deep learning-based prediction of drug-induced cardiotoxicity," *Journal of Chemical Information and Modeling*, vol. 59, p. 1073–1084, Feb. 2019.

[79] J. Hu, M. Huang, N. Ono, Y. Chen-Izu, L. T. Izu, and S. Kanaya, "Cardiotoxicity prediction based on integreted herg database with molecular convolution model," in *2019 IEEE International Conference on Bioinformatics and Biomedicine (BIBM)*, p. 1500–1503, IEEE, Nov. 2019.

[80] L. S. K. Konda, S. Keerthi Praba, and R. Kristam, "herg liability classification models using machine learning techniques," *Computational Toxicology*, vol. 12, p. 100089, Nov. 2019.

[81] H.-M. Lee, M.-S. Yu, S. R. Kazmi, S. Y. Oh, K.-H. Rhee, M.-A. Bae, B. H. Lee, D.-S. Shin, K.-S. Oh, H. Ceong, D. Lee, and D. Na, "Computational determination of herg-related cardiotoxicity of drug candidates," *BMC Bioinformatics*, vol. 20, May 2019.

[82] S. Munawar, J. I. Vandenberg, and I. Jabeen, "Molecular docking guided grid-independent descriptor analysis to probe the impact of water molecules on conformational changes of herg inhibitors in drug trapping phenomenon," *International Journal of Molecular Sciences*, vol. 20, p. 3385, July 2019.

[83] T. Negami, M. Araki, Y. Okuno, and T. Terada, "Calculation of absolute binding free energies between the herg channel and structurally diverse drugs," *Scientific Reports*, vol. 9, Nov. 2019.

[84] K. Ogura, T. Sato, H. Yuki, and T. Honma, "Support vector machine model for herg inhibitory activities based on the integrated herg database using descriptor selection by nsga-ii," *Scientific Reports*, vol. 9, Aug. 2019.

[85] Y. Zhang, J. Zhao, Y. Wang, Y. Fan, L. Zhu, Y. Yang, X. Chen, T. Lu, Y. Chen, and H. Liu, "Prediction of herg k+ channel blockage using deep neural networks," *Chemical Biology & Drug Design*, vol. 94, p. 1973–1985, Sept. 2019.

[86] K.-E. Choi, A. Balupuri, and N. S. Kang, "The study on the herg blocker prediction using chemical fingerprint analysis," *Molecules*, vol. 25, p. 2615, June 2020.

[87] N. Khalifa, L. S. Kumar Konda, and R. Kristam, "Machine learning-based qsar models to predict sodium ion channel (nav1.5) blockers," *Future Medicinal Chemistry*, vol. 12, p. 1829–1843, Oct. 2020.

[88] H. Kim and H. Nam, "herg-att: Self-attention-based deep neural network for predicting herg blockers," *Computational Biology and Chemistry*, vol. 87, p. 107286, Aug. 2020.

[89] M. Liu, L. Zhang, S. Li, T. Yang, L. Liu, J. Zhao, and H. Liu, "Prediction of herg potassium channel blockage using ensemble learning methods and molecular fingerprints," *Toxicology Letters*, vol. 332, p. 88–96, Oct. 2020.

[90] J. Y. Ryu, M. Y. Lee, J. H. Lee, B. H. Lee, and K.-S. Oh, "Deephit: a deep learning framework for prediction of herg-induced cardiotoxicity," *Bioinformatics*, vol. 36, p. 3049–3055, Feb. 2020.

[91] V. B. Siramshetty, D.-T. Nguyen, N. J. Martinez, N. T. Southall, A. Simeonov, and A. V. Zakharov, "Critical assessment of artificial intelligence methods for prediction of herg channel inhibition in the "big data" era," *Journal of Chemical Information and Modeling*, vol. 60, p. 6007–6019, Dec. 2020.

[92] Y. Wang, L. Huang, S. Jiang, Y. Wang, J. Zou, H. Fu, and S. Yang, "Capsule networks showed excellent performance in the classification of herg blockers/nonblockers," *Frontiers in Pharmacology*, vol. 10, Jan. 2020.

[93] T. M. Creanza, P. Delre, N. Ancona, G. Lentini, M. Saviano, and G. F. Mangiatordi, "Structure-based prediction of herg-related cardiotoxicity: A benchmark study," *Journal of Chemical Information and Modeling*, vol. 61, p. 4758–4770, Sept. 2021.

[94] A. Karim, M. Lee, T. Balle, and A. Sattar, "Cardiotox net: a robust predictor for herg channel blockade based on deep learning meta-feature ensembles," *Journal of Cheminformatics*, vol. 13, Aug. 2021.

[95] K. H. Lee, A. D. Fant, J. Guo, A. Guan, J. Jung, M. Kudaibergenova, W. E. Miranda, T. Ku, J. Cao, S. Wacker, H. J. Duff, A. H. Newman, S. Y. Noskov, and L. Shi, "Toward reducing herg affinities for dat inhibitors with a combined machine learning and molecular modeling approach," *Journal of Chemical Information and Modeling*, vol. 61, p. 4266–4279, Aug. 2021.

[96] J. Meng, L. Zhang, L. Wang, S. Li, D. Xie, Y. Zhang, and H. Liu, "Tssf-herg: A machine-learning-based herg potassium channel-specific scoring function for chemical cardiotoxicity prediction," *Toxicology*, vol. 464, p. 153018, Dec. 2021.

[97] N. Hari Narayana Moorthy, C. Karthikeyan, and E. Manivannan, "Multi-algorithm based machine learning and structural pattern studies for herg ion channel blockers mediated cardiotoxicity prediction," *Chemometrics and Intelligent Laboratory Systems*, vol. 208, p. 104213, Jan. 2021.

[98] Sato, "Quantitative prediction of herg inhibitory activities using support vector regression and the integrated herg dataset in amed cardiotoxicity database," *Chem-Bio Informatics Journal*, vol. 21, pp. 70–80, 2021.

[99] C. Stergiopoulos, F. Tsopelas, and K. Valko, "Prediction of herg inhibition of drug discovery compounds using biomimetic hplc measurements," *ADMET and DMPK*, June 2021.

[100] Z. Wu, D. Jiang, J. Wang, C.-Y. Hsieh, D. Cao, and T. Hou, "Mining toxicity information from large amounts of toxicity data," *Journal of Medicinal Chemistry*, vol. 64, p. 6924–6936, May 2021.

[101] G. Xiong, Z. Wu, J. Yi, L. Fu, Z. Yang, C. Hsieh, M. Yin, X. Zeng, C. Wu, A. Lu, X. Chen, T. Hou, and D. Cao, "Admetlab 2.0: an integrated online platform for accurate and comprehensive predictions of admet properties," *Nucleic Acids Research*, vol. 49, p. W5–W14, Apr. 2021.

[102] I. Arab and K. Barakat, "Toxtree: descriptor-based machine learning models for both herg and nav1.5 cardiotoxicity liability predictions," 2021.

[103] P. Delre, G. J. Lavado, G. Lamanna, M. Saviano, A. Roncaglioni, E. Benfenati, G. F. Mangiatordi, and D. Gadaleta, "Ligand-based prediction of herg-mediated cardiotoxicity based on the integration of different machine learning techniques," *Frontiers in Pharmacology*, vol. 13, Sept. 2022.

[104] W. Ding, Y. Nan, J. Wu, C. Han, X. Xin, S. Li, H. Liu, and L. Zhang, "Combining multi-dimensional molecular fingerprints to predict the herg cardiotoxicity of compounds," *Computers in Biology and Medicine*, vol. 144, p. 105390, May 2022.

[105] I. Kazuaki, S. Hideko, H. Kohsuke, and H. Kohsuke, "Gradient boosting machine-based model for predicting herg k+ channel inhibitory activities," *Journal of Computer Science Applications and Information Technology*, vol. 5, no. 1, pp. 1–9, 2022.

[106] H. Kim, M. Park, I. Lee, and H. Nam, "Bayesherg: a robust, reliable and interpretable deep learning model for predicting herg channel blockers," *Briefings in Bioinformatics*, vol. 23, June 2022.

[107] W. Kong, W. Huang, C. Peng, B. Zhang, G. Duan, W. Ma, and Z. Huang, "Multiple machine learning methods aided virtual screening of nav1.5 inhibitors," *Journal of Cellular and Molecular Medicine*, vol. 27, p. 266–276, Dec. 2022.

[108] S. Krishna, A. Borrel, R. Huang, J. Zhao, M. Xia, and N. Kleinstreuer, "High-throughput chemical screening and structure-based models to predict herg inhibition," *Biology*, vol. 11, p. 209, Jan. 2022.

[109] K. Lanevskij, R. Didziapetris, and A. Sazonovas, "Physicochemical qsar analysis of herg inhibition revisited: towards a quantitative potency prediction," *Journal of Computer-Aided Molecular Design*, vol. 36, p. 837–849, Oct. 2022.

[110] F. Melnikov, L. T. Anger, and C. Hasselgren, "Toward quantitative models in safety assessment: A case study to show impact of dose–response inference on herg inhibition models," *International Journal of Molecular Sciences*, vol. 24, p. 635, Dec. 2022.

[111] M. Shan, C. Jiang, J. Chen, L.-P. Qin, J.-J. Qin, and G. Cheng, "Predicting herg channel blockers with directed message passing neural networks," *RSC Advances*, vol. 12, no. 6, p. 3423–3430, 2022.

[112] X. Zhang, J. Mao, M. Wei, Y. Qi, and J. Z. H. Zhang, "Hergspred: Accurate classification of herg blockers/nonblockers with machine-learning models," *Journal of Chemical Information and Modeling*, vol. 62, p. 1830–1839, Apr. 2022.

[113] I. Arab, K. Egghe, K. Laukens, K. Chen, K. Barakat, and W. Bittremieux, "Benchmarking of small molecule feature representations for herg, nav1.5, and cav1.2 cardiotoxicity prediction," *Journal of Chemical Information and Modeling*, vol. 64, p. 2515–2527, Oct. 2023.

[114] A. Banerjee and K. Roy, "Machine-learning-based similarity meets traditional qsar: "q-rasar" for the enhancement of the external predictivity and detection of prediction confidence outliers in an herg toxicity dataset," *Chemometrics and Intelligent Laboratory Systems*, vol. 237, p. 104829, June 2023.

[115] Y. Chen, X. Yu, W. Li, Y. Tang, and G. Liu, "In silico prediction of herg blockers using machine learning and deep learning approaches," *Journal of Applied Toxicology*, vol. 43, p. 1462–1475, May 2023.

[116] N. R. Das, T. Sharma, A. A. Toropov, A. P. Toropova, M. K. Tripathi, and P. G. R. Achary, "Machine-learning technique, qsar and molecular dynamics for herg–drug interactions," *Journal of Biomolecular Structure and Dynamics*, vol. 41, p. 13766–13791, Apr. 2023.

[117] H. Feng and G.-W. Wei, "Virtual screening of drugbank database for herg blockers using topological laplacian-assisted ai models," *Computers in Biology and Medicine*, vol. 153, p. 106491, Feb. 2023.

[118] T. Wang, J. Sun, and Q. Zhao, "Investigating cardiotoxicity related with herg channel blockers using molecular fingerprints and graph attention mechanism," *Computers in Biology and Medicine*, vol. 153, p. 106464, Feb. 2023.

[119] H. Wang, G. Zhu, L. T. Izu, Y. Chen-Izu, N. Ono, M. Altaf-Ul-Amin, S. Kanaya, and M. Huang, "On qsar-based cardiotoxicity modeling with the expressiveness-enhanced graph learning model and dual-threshold scheme," *Frontiers in Physiology*, vol. 14, May 2023.

[120] S. Vittorio, F. Lunghini, A. Pedretti, G. Vistoli, and A. R. Beccari, "Ensemble of structure and ligand-based classification models for herg liability profiling," *Frontiers in Pharmacology*, vol. 14, Mar. 2023.

[121] E. Ylipää, S. Chavan, M. Bånkestad, J. Broberg, B. Glinghammar, U. Norinder, and I. Cotgreave, "herg-toxicity prediction using traditional machine learning and advanced deep learning techniques," *Current Research in Toxicology*, vol. 5, p. 100121, 2023.

[122] I. Arab, K. Laukens, and W. Bittremieux, "Semisupervised learning to boost herg, nav1.5, and cav1.2 cardiac ion channel toxicity prediction by mining a large unlabeled small molecule data set," *Journal of Chemical Information and Modeling*, vol. 64, p. 6410–6420, Aug. 2024.

[123] Z. Chen, N. Li, P. Zhang, Y. Li, and X. Li, "Cardiodpi: An explainable deep-learning model for identifying cardiotoxic chemicals targeting herg, cav1.2, and nav1.5 channels," *Journal of Hazardous Materials*, vol. 474, p. 134724, Aug. 2024.

[124] S. He, X. Ye, and T. Sakurai, "Clop-herg: The contrastive learning optimized pre-trained model for representation learning in predicting drug-induced herg channel blockers," *Medinformatics*, vol. 1, p. 103–111, Jan. 2024.

[125] J. Liu, M. K. H. Khan, W. Guo, F. Dong, W. Ge, C. Zhang, P. Gong, T. A. Patterson, and H. Hong, "Machine learning and deep learning approaches for enhanced prediction of herg blockade: a comprehensive qsar modeling study," *Expert Opinion on Drug Metabolism & Toxicology*, vol. 20, p. 665–684, July 2024.

[126] I. H. Sanches, R. C. Braga, V. M. Alves, and C. H. Andrade, "Enhancing herg risk assessment with interpretable classificatory and regression models," *Chemical Research in Toxicology*, vol. 37, p. 910–922, May 2024.

[127] T. Wang, Z. Du, L. Zhuo, X. Fu, Q. Zou, and X. Yao, "Multicblo: Enhancing predictions of compound-induced inhibition of cardiac ion channels with advanced multimodal learning," *International Journal of Biological Macromolecules*, vol. 276, p. 133825, Sept. 2024.

[128] T. Yang, X. Ding, E. McMichael, F. W. Pun, A. Aliper, F. Ren, A. Zhavoronkov, and X. Ding, "Attenherg: a reliable and interpretable graph neural network framework for predicting herg channel blockers," *Journal of Cheminformatics*, vol. 16, Dec. 2024.

[129] G. Falcón-Cano, A. Morales-Helguera, H. Lambert, M.-A. Cabrera-Pérez, and C. Molina, "herg toxicity prediction in early drug discovery using extreme gradient boosting and isometric stratified ensemble mapping," *Scientific Reports*, vol. 15, May 2025.

[130] L. Feng, X. Fu, Z. Du, Y. Guo, L. Zhuo, Y. Yang, D. Cao, and X. Yao, "Multictox: Empowering accurate cardiotoxicity prediction through adaptive multimodal learning," *Journal of Chemical Information and Modeling*, vol. 65, p. 3517–3528, Mar. 2025.

[131] N. Gambacorta, F. Mastrolorito, M. V. Togo, V. Amenduni, M. Mele, A. Liantonio, A. Mele, A. De Luca, C. D. Altomare, V. Belgiovine, A. R. Tondo, F. Cutropia, L. Siragusa, N. Amoroso, F. Ciriaco, P. Imbrici, D. Trisciuzzi, and O. Nicolotti, "Cupid: A free drug discovery platform for the explainable multi-ion channel assessment of cardiotoxicity," *European Journal of Medicinal Chemistry*, vol. 290, p. 117575, June 2025.

[132] H. Han, B. Shaker, J. H. Lee, S. Choi, S. Yoon, M. Singh, S. Basith, M. Cui, S. Ahn, J. An, S. Kang, M. S. Yeom, and S. Choi, "Employing automated machine learning (automl) methods to facilitate the in silico admet properties prediction," *Journal of Chemical Information and Modeling*, vol. 65, p. 3215–3225, Mar. 2025.

[133] D. Hossain, F. Al Abir, and J. Y. Chen, "herg-ltn: A new paradigm in herg cardiotoxicity assessment using neuro-symbolic and generative ai embedding (megamolbart, llama3.2, gemini, deepseek) approach," *openRxiv*, Feb. 2025.

[134] B. Jin, J. Wang, X. Yang, L. Na, and Q. Zhao, "herg-mffgnn: An explainable deep learning model for predicting cardiotoxicity using multi-feature fusion and graph neural networks," *Interdisciplinary Sciences: Computational Life Sciences*, Sept. 2025.

[135] Y. Jing, Y. Zhang, G. Zhao, T. McGuire, J. Zhao, B. Gibbs, G. Hou, Z. Feng, Y. Xue, and X.-Q. Xie, "Graphdeep-herg: Graph neural network pharmacoanalytics for assessing herg-related cardiotoxicity," *Pharmaceutical Research*, vol. 42, p. 579–591, Mar. 2025.

[136] G. W. Kyro, M. T. Martin, E. D. Watt, and V. S. Batista, "Cardiogenai: a machine learning-based framework for re-engineering drugs for reduced herg liability," *Journal of Cheminformatics*, vol. 17, Mar. 2025.

[137] D. Lee and S. Yoo, "hergat: predicting herg blockers using graph attention mechanism through atom- and molecule-level interaction analyses," *Journal of Cheminformatics*, vol. 17, Jan. 2025.

[138] K. Liu, H. Cui, X. Yu, W. Li, and W. Han, "Predicting cardiotoxicity in drug development: A deep learning approach," *Journal of Pharmaceutical Analysis*, vol. 15, p. 101263, Aug. 2025.

[139] L. Liu, Q. Zhang, and Y. Wei, "Mtf-herg: A multi-type features fusion-based framework for predicting herg cardiotoxicity of compounds," *IEEE Transactions on Computational Biology and Bioinformatics*, vol. 22, p. 3202–3212, Nov. 2025.

[140] S. Mohammad, V. Chandrasekar, O. Aboumarzouk, A. Vikram Singh, and S. Prasad Dakua, "Leveraging machine and deep learning algorithms for herg blocker prediction," *IEEE Access*, vol. 13, p. 81018–81028, 2025.

[141] V.-K. Tran-Nguyen, U. F. Randriharimanamizara, and O. Taboureau, "Hergai: an artificial intelligence tool for structure-based prediction of herg inhibitors," *Journal of Cheminformatics*, vol. 17, July 2025.

[142] C. Xu, Y. Xu, Z. Hu, X. Zhao, W. Xie, W. Chen, and J. Pei, "Unveiling optimal molecular features for herg insights with automatic machine learning," *Journal of Pharmaceutical Analysis*, p. 101411, July 2025.

[143] H. Yang, J. Xiu, W. yan, K. Liu, H. Cui, Z. Wang, Q. He, Y. Gao, and W. Han, "Large language models as tools for molecular toxicity prediction: Ai insights into cardiotoxicity," *Journal of Chemical Information and Modeling*, vol. 65, p. 2268–2282, Feb. 2025.

[144] M.-S. Yu, J. Lee, Y. Lee, D. Cho, K.-S. Oh, J. Jang, N. T. Nong, H.-M. Lee, and D. Na, "hergboost: A gradient boosting model for quantitative ic50 prediction of herg channel blockers," *Computers in Biology and Medicine*, vol. 184, p. 109416, Jan. 2025.

[145] Y. Zhang, Y. Liu, W. Liu, and J. Chen, "Enlarged data sets and innovative applicability domain characterization empower ml models to reliably bridge herg binding data gaps in diverse chemicals," *Chemical Research in Toxicology*, vol. 38, p. 1460–1471, Aug. 2025.

[146] D. Agarwal, A. Sharma, and P. Garg, "Graph-based classification with gnn-explainer for predicting cardiac toxicity associated with multi-ion channel blockers," *Chemical Research in Toxicology*, vol. 39, p. 518–529, Mar. 2026.

[147] C. Beidja, A. Bousselham, Z. En-Naimani, and M. Mestari, "Improving herg cardiotoxicity prediction via smiles transformer, molecular fingerprints, and layer-wise self-adaptive graph attention network," *ACS Omega*, vol. 11, p. 17484–17497, Mar. 2026.

[148] J.-W. Chu, J.-H. Park, and Y.-R. Cho, "Memol: Mixture of experts for multimodal learning through multi-head attention to predict drug toxicity," *Computer Methods and Programs in Biomedicine*, vol. 273, p. 109088, Jan. 2026.

[149] T. Enokiya, R. Kunitomo, and T. Yamaguchi, "Structure-based prediction of qt prolongation risk using graph neural networks: An integrative approach combining in vitro herg assays and pharmacovigilance data," *Clinical Pharmacology & Therapeutics*, vol. 119, p. 1662–1670, Feb. 2026.

[150] M. Jovanović, L. Weidener, M. Brkić, E. Ulgac, and A. Meduri, "Cardiosafe: Multi-task prediction of cardiac ion channel activity with reverse-leak audited benchmarking," *openRxiv*, May 2026.

[151] K. Liang, R. Wang, and D. Qin, "Cardiotoxicity prediction of compounds based on multi-graph and multi-dimensional representations," *Chemometrics and Intelligent Laboratory Systems*, vol. 273, p. 105704, June 2026.

[152] Y. Su, J. Wu, A. Yang, Y. Yuan, W. Du, Y. Xiang, and W. Shen, "Mmrcl: An interpretable multi-modal deep learning framework for predicting herg blockers," *Computational Biology and Chemistry*, vol. 122, p. 108926, June 2026.

[153] H. Sun, Y. Wang, and M. Shen, "Modeling herg channel liability: From structural insight to highly accurate qualitative and quantitative models," *Journal of Chemical Information and Modeling*, vol. 66, p. 7515–7523, June 2026.

[154] Y. Zhang, Y. Liu, Z. Xiao, and J. Chen, "Counted fingerprint-enhanced graph neural network models enable accurate screening of herg blockers from diverse categories of chemicals," *Environmental Science & Technology*, vol. 60, p. 11542–11552, Mar. 2026.

[155] B. Zhao, Z. Chang, and M. Huo, "Feaof: A transferable framework applied to prediction of herg-related cardiotoxicity," *Computational Biology and Chemistry*, vol. 120, p. 108622, Feb. 2026.

[156] F. De Ponti, E. Poluzzi, and N. Montanaro, "Organising evidence on qt prolongation and occurrence of torsades de pointes with non-antiarrhythmic drugs: a call for consensus," *European Journal of Clinical Pharmacology*, vol. 57, p. 185–209, June 2001.

[157] S. Polak, B. Wiśniowska, and J. Brandys, "Collation, assessment and analysis of literature in vitro data on herg receptor blocking potency for subsequent modeling of drugs' cardiotoxic properties," *Journal of Applied Toxicology*, vol. 29, p. 183–206, Apr. 2009.

[158] T. Sato, H. Yuki, K. Ogura, and T. Honma, "Construction of an integrated database for herg blocking small molecules," *PLOS ONE*, vol. 13, p. e0199348, July 2018.

[159] B. Zdrazil, E. Felix, F. Hunter, E. J. Manners, J. Blackshaw, S. Corbett, M. de Veij, H. Ioannidis, D. M. Lopez, J. F. Mosquera, M. P. Magarinos, N. Bosc, R. Arcila, T. Kizilören, A. Gaulton, A. P. Bento, M. F. Adasme, P. Monecke, G. A. Landrum, and A. R. Leach, "The ChEMBL Database in 2023: a drug discovery platform spanning multiple bioactivity data types and time periods," *Nucleic Acids Research*, vol. 52, pp. D1180–D1192, 11 2023.

[160] Excelra, "GOSTARDB." https://www.gostardb.com, n.d. Commercial database; Accessed: 2026-01-05.

[161] S. Kim, J. Chen, T. Cheng, A. Gindulyte, J. He, S. He, Q. Li, B. Shoemaker, P. Thiessen, B. Yu, L. Zaslavsky, J. Zhang, and E. Bolton, "Pubchem 2025 update," *Nucleic Acids Research*, vol. 53, p. D1516–D1525, Nov. 2024.

[162] F. Du, H. Yu, B. Zou, J. Babcock, S. Long, and M. Li, "hergcentral: A large database to store, retrieve, and analyze compound-human ether-à-go-go related gene channel interactions to facilitate cardiotoxicity assessment in drug development," *ASSAY and Drug Development Technologies*, vol. 9, p. 580–588, Dec. 2011.

[163] M. K. Gilson, T. Liu, M. Baitaluk, G. Nicola, L. Hwang, and J. Chong, "Bindingdb in 2015: A public database for medicinal chemistry, computational chemistry and systems pharmacology," *Nucleic Acids Research*, vol. 44, p. D1045–D1053, Oct. 2015.

[164] R. Thomas, "The us federal tox21 program: A strategic and operational plan for continued leadership," *ALTEX*, p. 163–168, 2018.

[165] S. D. Harding, J. F. Armstrong, E. Faccenda, C. Southan, S. H. Alexander, A. P. Davenport, M. Spedding, and J. A. Davies, "The iuphar/bps guide to pharmacology in 2026," *Nucleic Acids Research*, Oct. 2025.

[166] C. Knox, M. Wilson, C. M. Klinger, M. Franklin, E. Oler, A. Wilson, A. Pon, J. Cox, N. E. Chin, S. A. Strawbridge, M. Garcia-Patino, R. Kruger, A. Sivakumaran, S. Sanford, R. Doshi, N. Khetarpal, O. Fatokun, D. Doucet, A. Zubkowski, D. Yahya Rayat, H. Jackson, K. Harford, A. Anjum, M. Zakir, F. Wang, S. Tian, B. Lee, J. Liigand, H. Peters, R. Q. Wang, T. Nguyen, D. So, M. Sharp, R. da Silva, C. Gabriel, J. Scantlebury, M. Jasinski, D. Ackerman, T. Jewison, T. Sajed, V. Gautam, and D. S. Wishart, "Drugbank 6.0: the drugbank knowledgebase for 2024," *Nucleic Acids Research*, vol. 52, pp. D1265–D1275, 1 2024.

[167] R. R. Fenichel, "Receptor binding." https://www.fenichel.net/pages/Professional/subpages/QT/Tables/pbydrug.htm, 2010. Last updated: 2010; Accessed: 2026-01-05.

[168] I. Sushko, S. Novotarskyi, R. Körner, A. K. Pandey, M. Rupp, W. Teetz, S. Brandmaier, A. Abdelaziz, V. V. Prokopenko, V. Y. Tanchuk, R. Todeschini, A. Varnek, G. Marcou, P. Ertl, V. Potemkin, M. Grishina, J. Gasteiger, C. Schwab, I. I. Baskin, V. A. Palyulin, E. V. Radchenko, W. J. Welsh, V. Kholodovych, D. Chekmarev, A. Cherkasov, J. Aires-de Sousa, Q.-Y. Zhang, A. Bender, F. Nigsch, L. Patiny, A. Williams, V. Tkachenko, and I. V. Tetko, "Online chemical modeling environment (ochem): web platform for data storage, model development and publishing of chemical information," *Journal of Computer-Aided Molecular Design*, vol. 25, p. 533–554, June 2011.

[169] R. L. Woosley, C. W. Heise, T. Gallo, R. D. Woosley, J. Lambson, and K. A. Romero, "Qtdrugs list." www.CredibleMeds.org. Accessed: 2026-01-05.

[170] Computational Toxicology Group, "Tox-portal: Toxicological databases and on-line toxicity prediction algorithms." https://tox-portal.com/#/, n.d. Free access toxicological database focusing on cardiovascular toxicity and neurotoxicity; Accessed: 2026-01-05.

[171] M. Olah, R. Rad, L. Ostopovici, A. Bora, N. Hadaruga, D. Hadaruga, R. Moldovan, A. Fulias, M. Mractc, and T. I. Oprea, "Wombat and wombat-pk: Bioactivity databases for lead and drug discovery," Feb. 2007.

[172] W. J. Crumb, J. Vicente, L. Johannesen, and D. G. Strauss, "An evaluation of 30 clinical drugs against the comprehensive in vitro proarrhythmia assay (cipa) proposed ion channel panel," *Journal of Pharmacological and Toxicological Methods*, vol. 81, p. 251–262, Sept. 2016.

[173] K. Huang, T. Fu, W. Gao, Y. Zhao, Y. Roohani, J. Leskovec, C. W. Coley, C. Xiao, J. Sun, and M. Zitnik, "Therapeutics data commons: Machine learning datasets and tasks for drug discovery and development," *arXiv*, 2021.

[174] A. and Bateman, M.-J. Martin, S. Orchard, M. Magrane, A. Adesina, S. Ahmad, E. H. Bowler-Barnett, H. Bye-A-Jee, D. Carpentier, P. Denny, J. Fan, P. Garmiri, L. J. d. C. Gonzales, A. Hussein, A. Ignatchenko, G. Insana, R. Ishtiaq, V. Joshi, D. Jyothi, S. Kandasaamy, A. Lock, A. Luciani, J. Luo, Y. Lussi, J. S. M. Marin, P. Raposo, D. L. Rice, R. Santos, E. Speretta, J. Stephenson, P. Totoo, N. Tyagi, N. Urakova, P. Vasudev, K. Warner, S. Wijerathne, C. W.-H. Yu, R. Zaru, A. J. Bridge, L. Aimo, G. Argoud-Puy, A. H. Auchincloss, K. B. Axelsen, P. Bansal, D. Baratin, T. M. Batista Neto, M.-C. Blatter, J. T. Bolleman, E. Boutet, L. Breuza, B. C. Gil, C. Casals-Casas, K. C. Echioukh, E. Coudert, B. Cuche, E. de Castro, A. Estreicher, M. L. Famiglietti, M. Feuermann, E. Gasteiger, P. Gaudet, S. Gehant, V. Gerritsen, A. Gos, N. Gruaz, C. Hulo, N. Hyka-Nouspikel, F. Jungo, A. Kerhornou, P. L. Mercier, D. Lieberherr, P. Masson, A. Morgat, S. Paesano, I. Pedruzzi, S. Pilbout, L. Pourcel, S. Poux, M. Pozzato, M. Pruess, N. Redaschi, C. Rivoire, C. J. A. Sigrist, K. Sonesson, S. Sundaram, A. Sveshnikova, C. H. Wu, C. N. Arighi, C. Chen, Y. Chen, H. Huang, K. Laiho, M. Lehvaslaiho, P. McGarvey, D. A. Natale, K. Ross, C. R. Vinayaka, Y. Wang, and J. Zhang, "Uniprot: the universal protein knowledgebase in 2025," *Nucleic Acids Research*, vol. 53, p. D609–D617, Nov. 2024.
